# A Syd and RUFY dynein adaptor complex mediates axonal circulation of dense core vesicles

**DOI:** 10.1083/jcb.202507071

**Published:** 2026-01-06

**Authors:** Viktor Karlovich Lund, Antony Chirco, Michela Caliari, Andreas Haahr Larsen, Kristoffer Tollestrup Tang, Ulrik Gether, Kenneth Lindegaard Madsen, Michael Wierer, Ole Kjaerulff

**Affiliations:** 1 Department of Neuroscience, University of Copenhagen, Copenhagen N, Denmark; 2 Proteomics Research Infrastructure, University of Copenhagen, Copenhagen N, Denmark; 3 Niels Bohr Institute, University of Copenhagen, Copenhagen N, Denmark

## Abstract

Neuropeptide-containing dense core vesicles (DCVs) generated in neuronal somata are circulated in axons to supply distal release sites, depending on kinesin-1, kinesin-3, and dynein, but how the motors are recruited remains unclear. Here we use proximity proteomics in the living *Drosophila* nervous system to identify the protein complex responsible for recruitment of kinesin-1 and dynein on DCVs. We find that the dynein and kinesin-1 adaptor Sunday driver (Syd/dJIP3/4) interact with the DCV-located GTPase Rab2 and also bind the Arl8 effector RUFY. Disrupting Rab2, Syd, RUFY, the Arl8 activator BORC, or dynein impedes retrograde DCV flux and induces axonal accumulation of immobile DCVs. Our data suggest that dynein is recruited and activated by a Syd/RUFY complex anchored to DCVs by Rab2 and Arl8. Rab2 loss but not disruption of Syd, RUFY, or dynein causes missorting of DCV membrane proteins into vesicle aggregates in motor neuron somata, suggesting that Rab2 employs separate effectors in DCV biogenesis and motility.

## Teaser

Lund et al. reveal that coupling of neuropeptide-containing dense core vesicles undergoing axonal transport to kinesin-1 and dynein is mediated by an adaptor complex composed of Syd/dJIP3/4 and RUFY, controlled by Rab2, Arl8, and the Arl8 activator BORC. Rab2-dependent sorting of membrane cargo to the vesicles is independent of the Syd–RUFY adaptor complex.

## Introduction

In neurons, long-distance axonal transport along microtubules (MTs) mediated by molecular motors is critical for enabling axonal outgrowth, maintaining synaptic function, and ensuring structural and functional synaptic plasticity. Neuropeptide/neurohormone-containing dense core vesicles (DCVs) constitute a particular logistical challenge in this context since, unlike small synaptic vesicles (SVs) that are generated locally at the presynapse through endocytosis, DCVs are produced at the trans-Golgi network (TGN) in the soma and completely rely on axonal transport for delivery to distal synaptic release sites. Work in *Drosophila* (Wong et al., 2012) and hippocampal neurons (Bharat et al., 2017) indicates that DCVs circulate throughout the axonal arbor, only reversing direction at distal axonal termini and the proximal axon, and with only a relatively small probability of being deposited in any given synaptic bouton as they traverse it. This arrangement has been suggested to ensure an even distribution of DCVs between synaptic boutons within the arbor (Moughamian and Holzbaur, 2012; Wong et al., 2012). Moreover, the circulating DCVs represent a large reserve pool that can be quickly drawn upon by increasing synaptic capture when synaptic release sites are depleted (Cavolo et al., 2016; Shakiryanova et al., 2006; Wong et al., 2009). This means that anterograde transport (toward synaptic termini) and retrograde transport (toward the soma) are equally important for supplying synaptic boutons.

Axonal MTs are universally oriented with plus ends out toward distal axonal termini and minus ends toward the soma. Anterograde transport is mediated by plus end–directed kinesin family motors, while retrograde transport is mediated by the minus end–directed cytoplasmic dynein motor (Guedes-Dias and Holzbaur, 2019). These motors are usually autoinhibited in their native, non-cargo–coupled state, and motor activation and cargo attachment are regulated by a large set of cargo- and motor-specific accessory and adaptor proteins and often involve small GTPases of the Rab and Arf/Arl families (Cason and Holzbaur, 2022; Maday et al., 2014). Rab and Arf/Arl proteins behave like molecular switches, cycling between an inactive GDP-loaded soluble state and an active GTP-loaded state where they are inserted into specific organellar membranes and recruit specific effector proteins such as tethers, vesicle coats, and motor adaptors (Arrazola Sastre et al., 2021).

In both invertebrates and mammals, axonal DCV transport depends on the fast kinesin-3 and the slower kinesin-1 anterograde motors, as well as dynein (Barkus et al., 2008; Gavrilova et al., 2024; Gumy et al., 2017; Lim et al., 2017; Lo et al., 2011). Kinesin-3 is required to traverse a pre-axonal filtering region for cell body exit (Barkus et al., 2008; Bharat et al., 2017; Gumy et al., 2017) and is regulated by the DCV-resident (Lund et al., 2021) small GTPase Arl8, which binds and activates it directly (Niwa et al., 2016; Vukoja et al., 2018). However, it is less clear how kinesin-1 and dynein are recruited to DCVs to maintain DCV circulation (Cason and Holzbaur, 2022).

A potential clue to the mechanism governing retrograde DCV transport comes from our finding that the small GTPase Rab2 is required for axonal transport of DCVs and lysosomes in *Drosophila* (Lund et al., 2021). Notably, although we formerly speculated that Rab2 may control kinesin-3, the Rab2 loss-of-function phenotype was characterized by a strikingly selective reduction in retrograde DCV transport, implying that Rab2 may play a role in dynein regulation (Lund et al., 2021). Moreover, Rab2 overexpression caused the redistribution of DCVs from the axono-synaptic compartment to the soma in pupal neurons releasing the bursicon neuropeptide hormone (Lund et al., 2021). Rab2 is a highly conserved member of the Rab protein family, which, apart from its involvement in organelle motility, is indispensable for lysosomal function (Lorincz et al., 2017; Lund et al., 2018), autophagy (Ding et al., 2019; Fujita et al., 2017), synaptic protein sorting (Götz et al., 2021), and DCV biogenesis (Ailion et al., 2014; Buffa et al., 2008; Edwards et al., 2009; Sumakovic et al., 2009). Work mostly done in *Caenorhabditis elegans* indicates that Rab2 and certain Rab2 effectors prevent the loss of a subset of DCV cargos to late endosomes/lysosomes during DCV maturation (Ailion et al., 2014; Edwards et al., 2009; Sumakovic et al., 2009). Despite the identification of many molecular components, the details of the Rab2-dependent DCV cargo sorting pathway remain mysterious, although an endosomal recycling mechanism seems to be involved (Laurent et al., 2018; Topalidou et al., 2016).

Here we use proximity proteomics to reveal that active Rab2 associates with the dynein/kinesin-1 adaptor Sunday driver (Syd/dJIP3/4) and the dynein adaptor RUFY1 (RUFY) in living fly neurons. Biochemical experiments using *Drosophila* proteins expressed in HEK293 (HEK) cells indicate that Rab2 physically interacts with Syd via the Syd RH2 cargo-binding domain, while RUFY binds Syd through the Syd C-terminal WD40 domain. Furthermore, defects in Rab2, Syd, RUFY, dynein, and partially kinesin-1 produce qualitatively similar effects on axonal transport of DCVs in fly motor neurons, characterized by a selective loss of retrograde transport and an increase in the proportion of static DCVs in the axons. In contrast, mutation of kinesin-3 primarily results in a strong symmetric reduction of anterograde and retrograde DCV fluxes due to a block in cell body exit, coupled with severe slowing of anterograde transport. We also show that, like the mammalian RUFY1-4 proteins, fly RUFY also binds Arl8, suggesting that the dynein–dynactin activating retrograde transport complex composed of Syd and RUFY is stabilized on DCVs in a Rab2- and Arl8-dependent manner. Consistent with this, knockout of the Arl8-activating BORC complex produces a phenotype closely resembling dynein/kinesin-1 loss-of-function phenotypes. Lastly, we find that in *Rab2* nulls, DCV membrane cargo is lost from DCVs and ectopically accumulates in phase-separated vesicle aggregates in neuronal somata. However, Syd, RUFY, and dynein are not responsible for the Rab2-dependent DCV membrane cargo sorting but may, together with Rab2, control DCV abundance.

## Results

### Proximity-dependent biotinylation identifies Syd/dJIP3/4 as a DCV-resident Rab2-interacting protein

**Figure 1.**
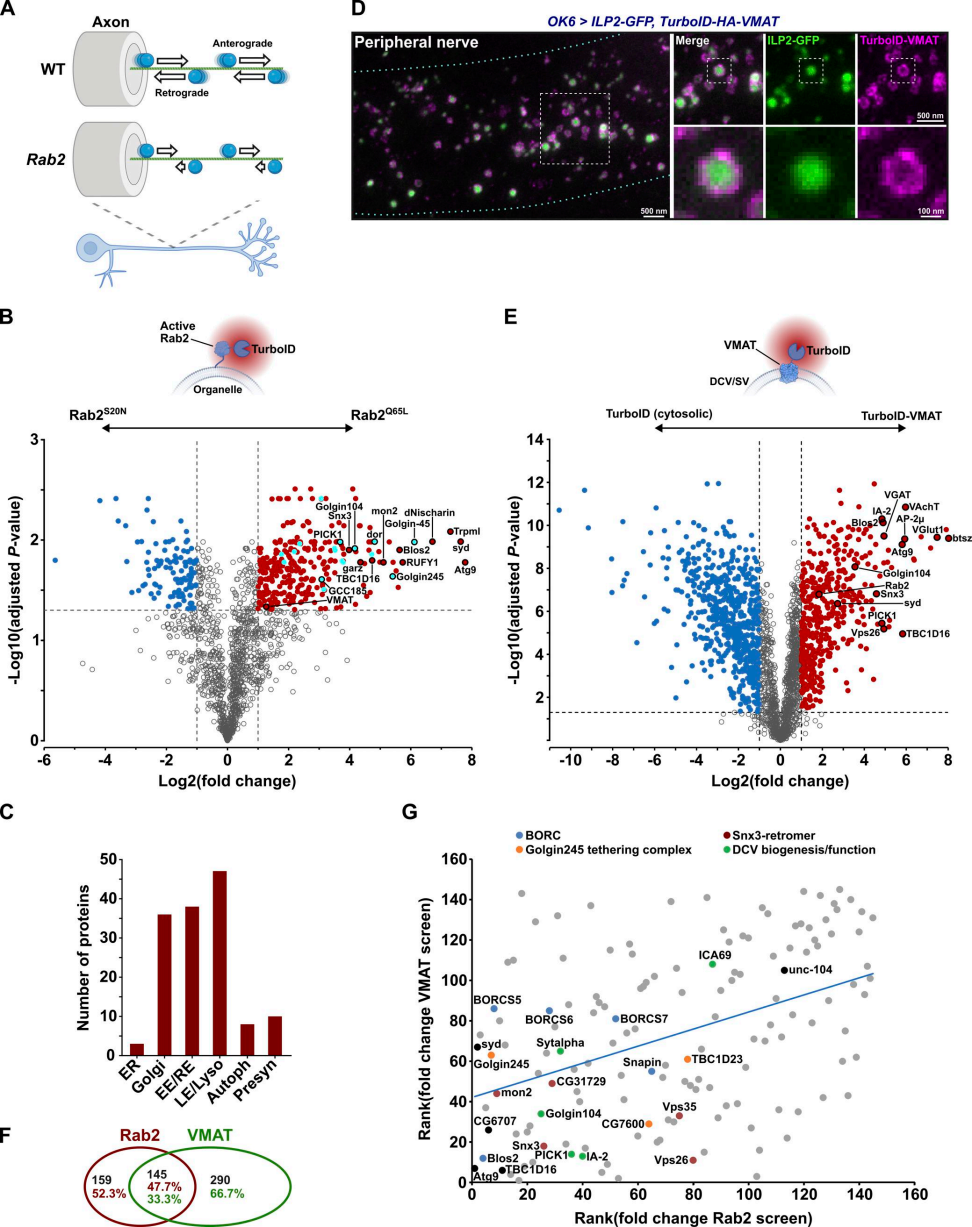
**In vivo neuron-specific PB/MS in *Drosophila* for detection of proteins interacting with active Rab2 and the DCV membrane protein VMAT. (A)** Schematic illustrating the effect of Rab2 loss on axonal transport of DCVs in flies (Lund et al., 2021). In WT motor axons, DCVs (blue spheres) are transported bidirectionally with similar anterograde and retrograde flux. In *Rab2* null mutants, retrograde flux is strongly reduced, while anterograde flux is partially reduced. **(B)** Volcano plot showing the fold change and Student’s *t* test statistics of biotinylated protein label free quantification (LFQ) intensities from flies with pan-neuronal expression of TurboID-Rab2^Q65L^ (*elav > 2xHA-TurboID-Rab2*^*Q65L*^) relative to TurboID-Rab2^S20N^ (*elav > 2xHA-TurboID-Rab2*^*S20N*^). Proteins significantly upregulated in the active Rab2 condition (fold change > 2, FDR-adjusted P value < 0.05) are highlighted in red*,* and known Rab2 effectors in light blue (see also Fig. S1). **(C)** Distribution in subcellular neuronal compartments of proteins specifically enriched in flies expressing TurboID-Rab2^Q65L^ compared with TurboID-Rab2^S20N^. EE, early endosomes; RE, recycling endosomes; LE, late endosomes; Lyso, lysosomes; Autoph, autophagosomes; Presyn, presynapse. **(D)** Representative STED image showing the distribution of ILP2-GFP and HA-tagged TurboID-VMAT fusion protein in motor axons in peripheral nerve A7 of third instar larva. Scale bars: left, 500 nm; right, 500 nm, 100 nm (inset). **(E)** Student’s *t* test of biotinylated protein LFQ intensities from flies with pan-neuronal expression of TurboID-VMAT (*elav > ILP2-GFP, TurboID-HA-VMAT*) relative to free cytosolic TurboID (*elav > ILP2-GFP, TurboID*). Proteins significantly enriched in the TurboID-HA-VMAT condition (fold change > 2, FDR-adjusted P value < 0.05) are highlighted in red. **(F)** Venn diagram of the overlap between proteins enriched both in flies expressing TurboID-Rab2^Q65L^ and TurboID-VMAT. **(G)** Relationship between proteins significantly enriched in TurboID-Rab2^Q65L^ and TurboID-VMAT flies, when ranked by enrichment level. Spearman’s rank correlation is 0.422, P < 10^−6^.

**Figure S1.**
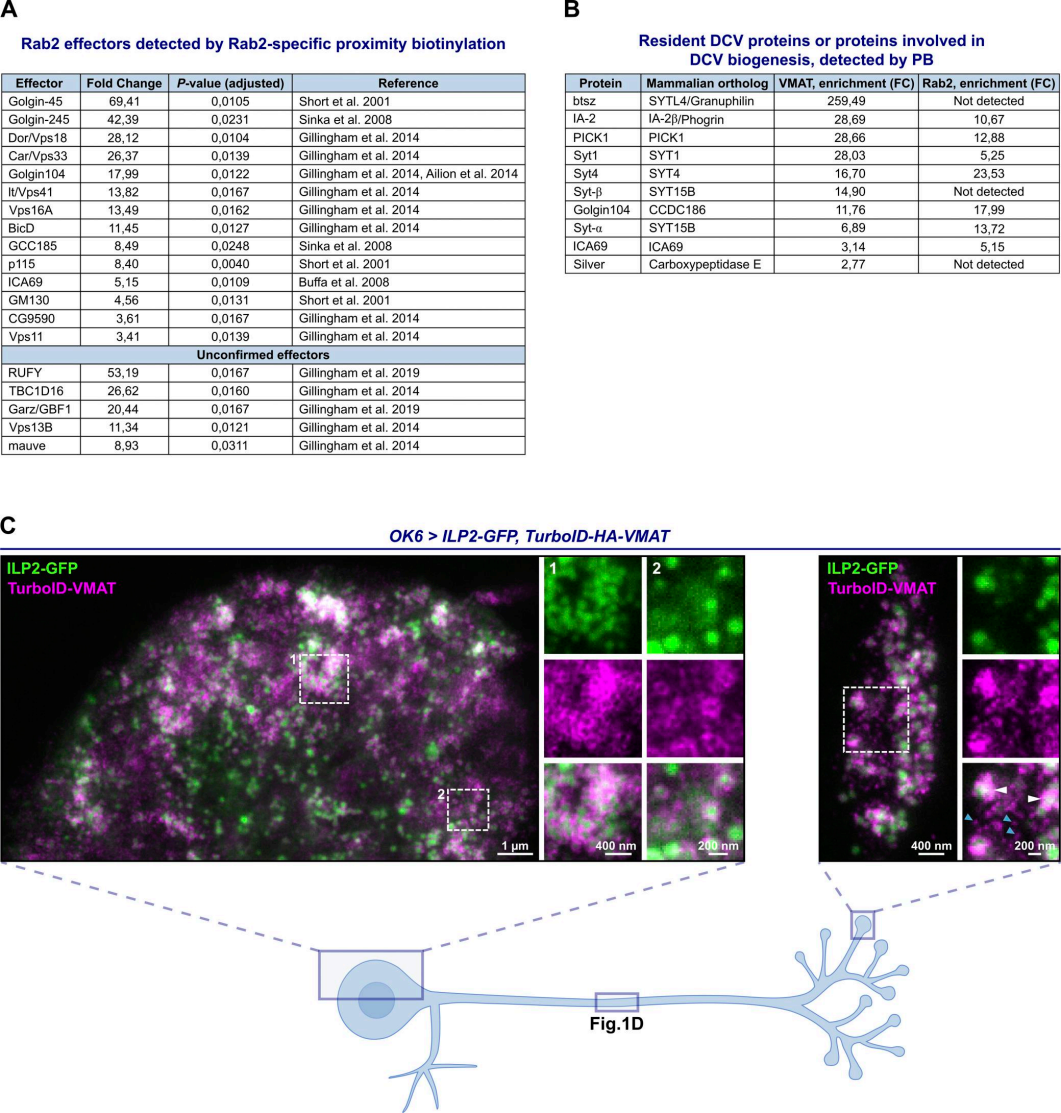
**Rab2- and VMAT-specific PB-MS results, and colocalization of ILP2-GFP and TurboID-VMAT in motor neuron soma and synaptic bouton. (A)** Previously identified Rab2 effectors (both *Drosophila* proteins and mammalian orthologs) found in our screen to be significantly enriched in TurboID-Rab2^Q65L^ samples relative to TurboID-Rab2^S20N^. First 14 entries, confirmed effectors (also labelled in Fig. 1 B). All subunits of the HOPS complex were counted as effectors. Last 5 entries, potential effectors detected by affinity proteomics in *Drosophila* S2 cells (Gillingham et al., 2014) and MitoID relocalization proximity proteomics in HEK cells (Gillingham et al., 2019) but not confirmed using other methods. The list of unconfirmed effectors is not exhaustive. **(B)** Proteins known to reside on DCVs or to be involved in DCV biogenesis that were significantly enriched in TurboID-VMAT samples relative to free TurboID. **(C)** Representative STED images showing the distribution of ILP2-GFP and TurboID-HA-VMAT in motor neuron cell body located in the dorsomedial aspect of the VNC (left) and in peripheral synaptic bouton (right) in a third instar larva. White arrowheads, TurboID-VMAT associated with ILP-GFP–positive DCVs. Blue arrowheads, small TurboID-VMAT–positive vesicles not associated with ILP-GFP. The distribution of ILP2-GFP and TurboID-HA-VMAT in the mid-axon region in the same type of preparation is shown in Fig. 1 D. Scale bars (left to right): 1µm, 400 nm (inset 1), 200 nm (inset 2), 400 nm, 200 nm (inset).

Rab2 is present on neuronal DCVs, and retrograde axonal transport of DCVs is severely and selectively disrupted in motor neurons of *Rab2* null third instar (L3) *Drosophila* larvae (Fig. 1 A) (Lund et al., 2021). We previously hypothesized that this reflects a function of Rab2 in the recruitment of molecular motors to the DCV surface through adaptor proteins. However, none of the known Rab2 effectors that could reasonably be expected to fill this role, such as the BicD dynein adaptor (Gillingham et al., 2014), were required for normal axonal DCV transport in flies (Lund et al., 2021). To identify potential novel effector proteins that could link activated Rab2 to motors responsible for DCV motility, we employed in vivo proximity-dependent biotinylation (PB) combined with quantitative mass spectrometry (MS), using a pan-neuronally expressed constitutively active GTP-locked TurboID-Rab2^Q65L^ chimera as bait (Fig. 1 B). TurboID is a promiscuous biotin ligase derived from BirA* (Branon et al., 2018), which, when fused to a protein of interest and expressed in a desired tissue, biotinylates proteins in its immediate vicinity (within ∼10 nm [Kim et al., 2014]) that can then be isolated using streptavidin. To filter out proteins not specifically interacting with the active form of Rab2, the MS signal of purified biotinylated neuronal proteins from TurboID-Rab2^Q65L^–expressing adult flies was compared with that of control flies expressing the inactive GDP-locked TurboID-Rab2^S20N^ variant. This approach identified ∼300 proteins significantly enriched more than twofold in the nano-environment of active neuronal Rab2 (Fig. 1 B, Data S1, and Data S2), including many known Rab2 effectors (Fig. S1 A). The remaining proteins in this group likely represent a mix of unknown effectors, constituents of effector complexes, and resident proteins of Rab2-associated vesicular compartments. The highest levels of enrichment were seen for proteins involved in lysosomal function and autophagy (with the most enriched protein being the transmembrane autophagy factor Atg9) (Fig. 1, B and C; and Data S2). This is in agreement with a critical role of Rab2 in lysosomal biogenesis and macroautophagy (Ding et al., 2019; Lorincz et al., 2017; Lund et al., 2018). Golgi apparatus–associated tethering proteins, many of them Rab2 effectors, were also well represented, consistent with the involvement of Rab2 in Golgi function (Götz et al., 2021; Short et al., 2001; Sinka et al., 2008). In addition, there was a strong representation of early and recycling endosomal proteins (Fig. 1 C and Data S2). Although this latter finding may in part reflect the difficulty of clearly differentiating between components belonging to the early and late stages of the endocytic pathway, it also fits with observations of Rab2 presence at a lower level throughout the endosomal system (Ding et al., 2019; Lund et al., 2018).

Strikingly, the second-most enriched protein for active Rab2 (∼200-fold enrichment over inactive Rab2, Fig. 1 B) was Syd, the fly ortholog of mammalian JIP3 and JIP4, which function as activating adaptors for dynein (Cason and Holzbaur, 2023; Singh et al., 2024) and also bind kinesin-1 (Bowman et al., 2000; Sun et al., 2011). Other notable highly enriched hits were RUFY1/CG31064 (∼50-fold enrichment; from hereon called RUFY), the fly ortholog of the mammalian RUFY1-4 family of coiled-coil proteins, recently shown to link Arl8 and Rab14 to dynein and possibly to function as dynein-activating adaptors (Keren-Kaplan et al., 2022; Kumar et al., 2022; Rawat et al., 2023); and the ortholog of the mammalian Rab14 effector Nischarin (CG11807, ∼100-fold enrichment; from hereon called dNischarin) that shows distant homology to the SKIP motor adaptor (Rosa-Ferreira and Munro, 2011). Interestingly, RUFY1 and RUFY2 were previously detected as unconfirmed potential effectors of human Rab2A, using the MitoID PB protocol (Gillingham et al., 2019).

As a separate strategy to identify motor adaptors responsible for DCV motility, we also sought to determine the in vivo surface proteome of neuronal DCVs using the DCV-resident membrane protein vesicular monoamine transporter (VMAT) as PB bait. To this end, we generated a transgene encoding *Drosophila* VMAT fused through its cytosolic N-terminal tail to TurboID. Nanoscopic examination using stimulated emission depletion (STED) microscopy showed that when expressed in larval motor neurons together with the lumenal DCV cargo marker ILP2-GFP (Wong et al., 2012), most TurboID-VMAT decorates the limiting membrane of ILP2-positive DCVs, which were ∼130 nm in diameter (135 ± 2.0 nm, mean ± SEM, *n* = 1,026 vesicles). The association of VMAT with DCVs was most clearly seen in axons (Fig. 1 D), where the density of organelles is relatively low, but was also observed in somata and in synaptic boutons (Fig. S1 C). In boutons, TurboID-VMAT was also present in ∼50 nm wide (52 ± 1.1 nm, mean ± SEM, *n* = 25) punctate structures, possibly corresponding to small SVs (Fig. S1 C). Comparative quantitative MS of biotinylated proteins from flies pan-neuronally expressing either TurboID-VMAT or a free cytosolic TurboID control transgene yielded ∼450 proteins significantly enriched in the TurboID-VMAT line (Fig. 1 E and Data S3). Among the PB hits enriched for TurboID-VMAT were well-known DCV membrane and peripheral membrane proteins such as IA-2 (Solimena et al., 1996) and bitesize/granuphilin/SYTL4 (Li et al., 2018; Torii et al., 2002; Yi et al., 2002) (with the latter showing the highest enrichment of all proteins), as well as proteins involved in DCV biogenesis (Fig. 1 E and Fig. S1 B). We also observed a strong enrichment for SV and endocytic proteins, consistent with VMAT also being targeted to SVs (Grygoruk et al., 2014). Both Syd and Rab2 (though not RUFY or dNischarin) were also significantly enriched in the TurboID-VMAT dataset (Fig. 1 E and Data S3).

Overall, the Rab2^Q65L^- and VMAT-enriched protein sets overlapped quite substantially (Fig. 1 F), consistent with Rab2 and VMAT functioning within the same compartment(s). Moreover, we observed a significant correlation between enrichment levels across the two PB datasets (Fig. 1 G). Interestingly, among the highly enriched proteins in both the Rab2^Q65L^ and VMAT screens were many components of the Snx3–retromer endosomal recycling complex (Harterink et al., 2011; McGough et al., 2018) and of a TGN vesicle tethering/fusion complex composed of TBC1D23, FAM91A1 (CG7600), and the Rab2-effector Golgin245 (Shin et al., 2017) (Fig. 1 G). This suggests that Rab2 may be involved in recycling of VMAT from endosomes to TGN. Other hits ranking high in both data sets included proteins related to DCV biogenesis and subunits of the BORC Arl8 activator complex (Fig. 1 G), consistent with the critical role of Arl8 in DCV motility (Lund et al., 2021).

Together, these data indicate that the dynein adaptors Syd and RUFY are spatially closely associated with active Rab2 in fly neurons in vivo and that Syd and Rab2 may be present together at the surface of DCVs.

### Syd interacts with Rab2 via its RH2 domain and behaves as a Rab2 effector

**Figure 2.**
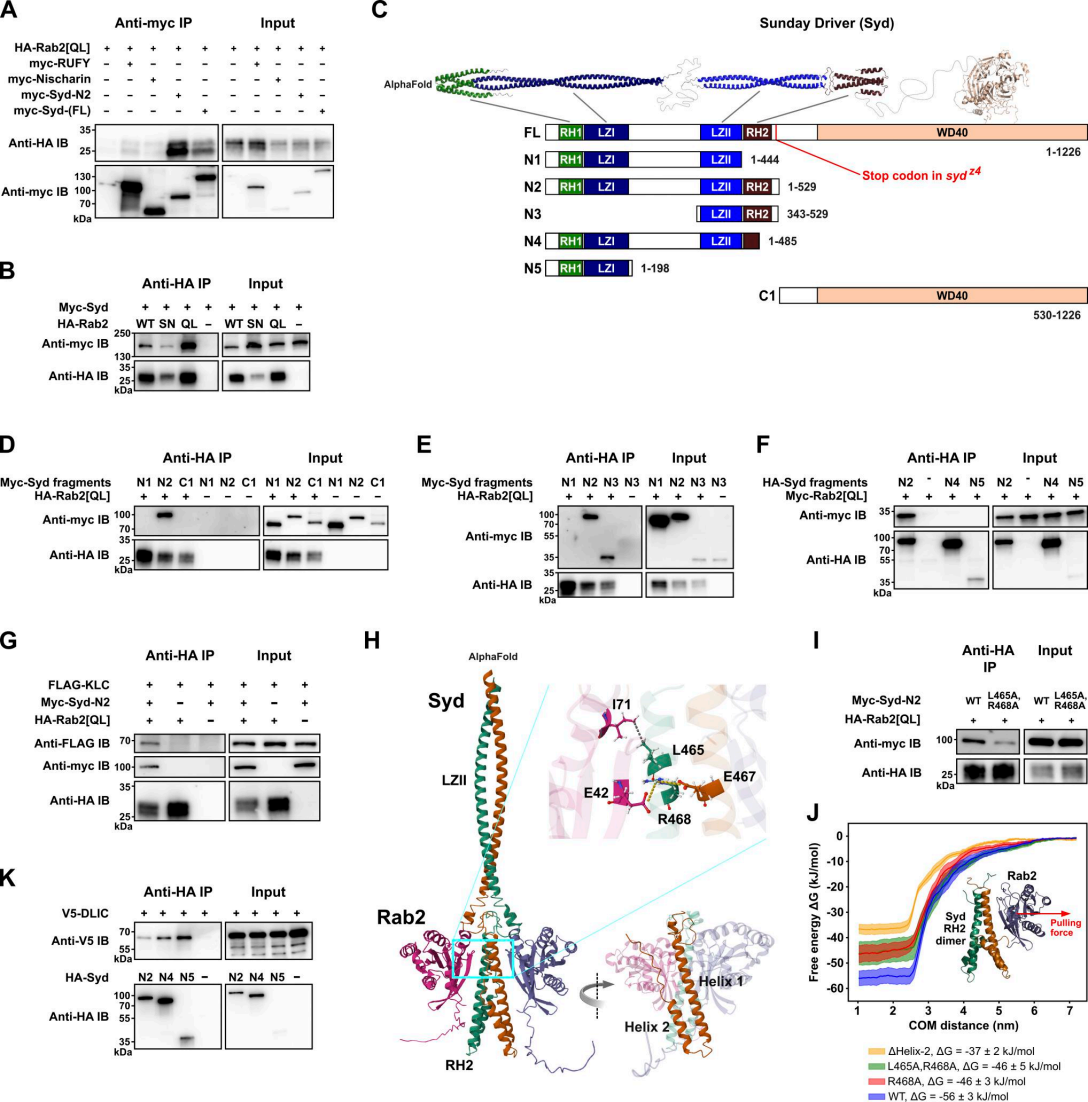
**Syd binds active Rab2 via the RH2 domain and also binds kinesin-1 and dynein motors.** Co-IP experiments performed on lysates from HEK cells transfected with constructs encoding epitope-tagged *Drosophila* proteins and MD simulation of the Syd:Rab2 interaction. **(A)** Myc-tagged full-length Syd and truncated Syd-N2 (Syd^1-529^) co-immunoprecipitate HA-tagged GTP-locked, constitutively active Rab2^Q65L^. In comparison, coprecipitation of HA-Rab2^Q65L^ by myc-tagged dNischarin and RUFY is near background levels. **(B)** WT HA-Rab2 and HA-Rab2^Q65L^ co-immunoprecipitate myc-Syd more efficiently than GDP-locked, inactive HA-Rab2^S20N^. **(C)** Structure of Syd. Top, expected structure of Syd homodimer assembled from three separate AlphaFold predictions. The WD40 domain of only one Syd monomer is shown. Bottom, schematic representation of the domain architecture of full-length Syd (isoform A, UniProt Q9GQF1) and of truncated Syd variants. **(D and E)** Co-IP of myc-tagged Syd fragments C1 and N1–N3 (shown in C) by HA-Rab2^Q65L^. Only myc-Syd-N2 and myc-Syd-N3, which contain the RH2 domain, coprecipitate with Rab2. **(F)** Co-IP of myc-Rab2^Q65L^ by HA-tagged Syd fragments N2, N4, and N5. **(G)** FLAG-tagged Klc co-immunoprecipitates in complex with myc-Syd-N2 and HA-Rab2^Q65L^ but not with HA-Rab2^Q65L^ alone. **(H)** AlphaFold prediction of Syd LZII-RH2 (Syd^359–526^) dimer in complex with two copies of Rab2. Inset, R468 in Syd RH2 helix 1 is predicted to engage in ionic interactions with E42 in the Rab2 switch I region and E467 in the other RH2 monomer helix 1. L465 is predicted to engage in a hydrophobic interaction with Rab2 switch II I71. **(I)** Comparison of Co-IP of WT myc-Syd-N2 and mutant myc-Syd-N2^L465A,R468A^ with HA-Rab2^Q65L^. **(J)** MD simulation. The Syd-RH2 dimer and one Rab2 moiety from the AlphaFold prediction in H were isolated in silico and pulled apart (the red arrow represents the pulling force direction) to estimate the free energy of the Rab2:Syd-RH2 binding. Free energy curves (mean and standard error, *n* = 10 for each curve) as a function of Rab2:RH2 center-of-mass distance were calculated for WT LZII-RH2 (blue), RH2^L465A,R468A^ (green), RH2^R468A^ (red), and RH2^ΔHelix-2^ (yellow), which was truncated after V504, removing the entire helix 2. **(K)** Co-IP of V5-tagged DLIC with HA-tagged Syd fragments N2, N4, and N5. Note the higher Co-IP efficiency for Syd-N4, where the C-terminal half of the RH2 domain (see Fig. S2 C) is absent, compared with Syd-N2, which has an intact RH2 domain. Source data are available for this figure: SourceData F2.

**Figure S2.**
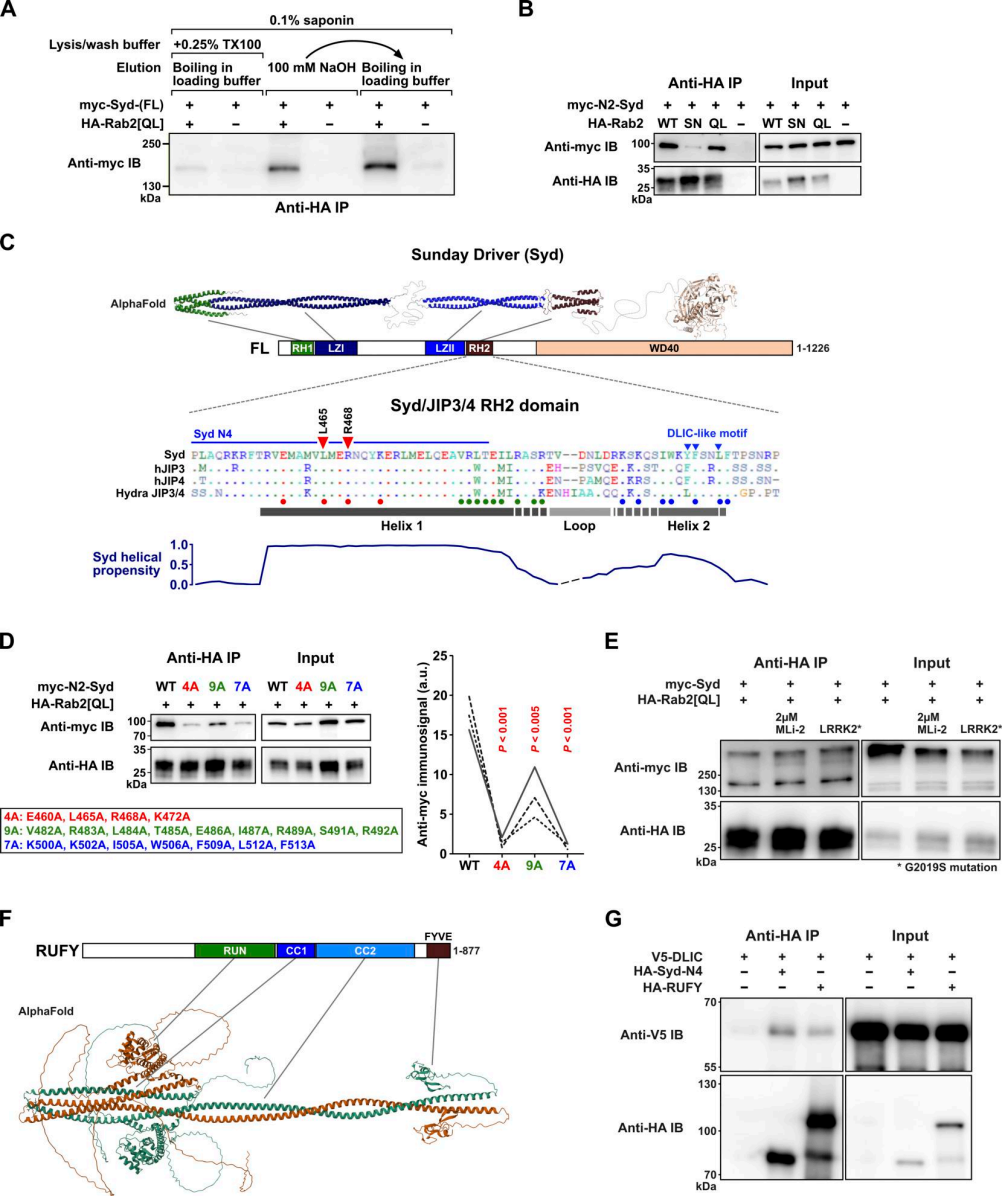
**Additional details of the Rab2-Syd Co-IP interaction, the predicted structure of RUFY, and RUFY-DLIC Co-IP results. (A)** Co-IP experiment performed on lysates from HEK cells transfected with constructs encoding epitope-tagged *Drosophila* proteins, illustrating the detergent sensitivity of the Rab2^Q65L^:Syd interaction. Western blot of eluates probed against myc showing coprecipitation of myc-Syd in the presence (lanes 1, 3, and 5) or absence (lanes 2, 4, and 6) of HA-Rab2^Q65L^ when immunoprecipitating against HA. In lanes 1–2, the experiment was performed in the presence of 0.1% saponin and 0.25% Triton X-100, and proteins were eluted from the anti-HA beads by boiling in SDS-PAGE loading buffer. In lanes 3–4, the experiment was performed only in the presence of 0.1% saponin, and proteins were eluted with 100 mM NaOH. In lanes 5–6, the same anti-HA beads that were eluted with 100 mM NaOH were boiled in SDS-PAGE loading buffer to elute the remaining protein. **(B)** Co-IP of myc-Syd-N2 by HA-Rab2, HA-Rab2^S20N^, and HA-Rab2^Q65L^. Compared with the experiment using full-length myc-Syd shown in Fig. 2 B, the amount of transfecting DNA-encoding HA-Rab2^S20N^ was increased to match the higher expression levels of HA-Rab2 and HA-Rab2^Q65L^. **(C)** Top, expected structure of Syd homodimer assembled from three separate AlphaFold predictions mapped onto the domain architecture of Syd. Middle, alignment of the RH2 domain from *Drosophila* Syd, human JIP3 and JIP4, and the cnidarian (*Hydra vulgaris*) JIP3/4 ortholog. Small dots in alignment indicate residue identity to Syd-RH2. The predicted locations of Helix 1, Helix 2, and the intervening loop from the AlphaFold model in Fig. 2 H are shown together with a helical propensity estimation (bottom). Also indicated are the location of the residues mutated to alanines in D (large colored dots below alignment) and Fig. 2 I (red triangles), the C-terminal extent of the Syd-N4 (Syd^1-485^) truncation, and the partially conserved DLIC-like motif involved in autoinhibition (Singh et al., 2024). **(D)** Left, Co-IP of WT myc-Syd-N2 and three different sets of myc-Syd-N2 alanine substitution mutants by HA-Rab2^Q65L^. The position of the mutations is indicated in C. Right, Quantification of the anti-myc immunosignal from eluted WT and mutated myc-Syd-N2 (*n* = three independent experiments). ANOVA, followed by Tukey’s test. **(E)** The effect of different levels of LRRK2 activity on Co-IP of full-length myc-Syd by HA-Rab2^Q65L^. Endogenous HEK cell LRRK2 activity was inhibited by treatment of cells with 2µM MLi-2 for 2 h before lysis, or increased by co-transfection with a constitutively active LRRK2^G2019S^ mutant. **(F)** Structure of an RUFY dimer predicted using AlphaFold 3, and the RUFY domain architecture. **(G)** Co-IP of V5-tagged DLIC with HA-tagged Syd-N4 (Syd^1-485^) and RUFY. Source data are available for this figure: SourceData FS2.

To test if the high levels of PB enrichment reflected physical interactions between Rab2 and Syd, RUFY and dNischarin, we performed co-immunoprecipitation (Co-IP) experiments using epitope-tagged versions of these proteins expressed in HEK cells. Only myc-tagged full-length Syd or truncated Syd^1-529^ (Syd-N2) coprecipitated Rab2^Q65L^ in appreciable amounts, with RUFY and dNischarin producing yields barely above background (Fig. 2 A). The Rab2:Syd interaction was relatively fragile, requiring a saponin-based lysis/binding buffer to achieve noticeable Co-IP yields (Fig. S2 A). This is likely why this interaction was not found in earlier Rab:effector affinity-proteomic screening (Gillingham et al., 2014) and may indicate that it requires additional protein or lipid components. However, in Co-IP experiments, Syd bound much stronger to active compared with inactive forms of Rab2, thus behaving as a classical Rab GTPase effector (Fig. 2 B and Fig. S2 B). We therefore continued the investigation of the Rab2:Syd interaction.

Structurally, Syd-family proteins (Syd/JIP3/JIP4) are large homodimers composed of an N-terminal region dominated by stretches of coiled-coil, followed by a C-terminal WD40 domain of unknown function (Fig. 2 C). The N-terminal half of Syd/JIP3/4 contains the RILP homology domains 1 and 2 (RH1 and RH2; also found in the distantly related RILP/RILPL family of adaptors), flanking two short leucine zipper domains (LZI and LZII) separated by a lengthy unstructured region (Fig. 2 C). The N-terminal RH1-LZI region of JIP3 binds in the cleft formed between dynein and dynactin and is sufficient to activate dynein motility (Singh et al., 2024). The more downstream LZII-RH2 region binds the kinesin-1 light chain (Klc) and Arf6 via the LZII domain (Bowman et al., 2000; Cockburn et al., 2018; Montagnac et al., 2009; Nguyen et al., 2005) and Rab8, 10, and 36 via the RH2 domain (Matsui et al., 2012; Waschbusch et al., 2020) and is thought to be responsible for cargo binding.

Consistent with this pattern, truncation mapping showed that an intact RH2 domain is required for Rab2 binding to Syd in Co-IP experiments (Fig. 2, C–F). Furthermore, while the isolated RH2 domain failed to express in HEK cells, a fragment consisting of only the LZII and RH2 domains (Syd-N3) was sufficient to be precipitated by active Rab2^Q65L^ (Fig. 2 E). Also, while Rab2^Q65L^ alone did not precipitate *Drosophila* Klc, it did so when co-overexpressed with Syd-N2 (Fig. 2 G), which contains all N-terminal coiled-coil regions but lacks the C-terminal region containing the WD40 domain (Fig. 2 C). This shows that Syd can bridge active Rab2 and molecular motors and that Rab2 binds Syd in a way that does not interfere with Klc binding at the LZII domain.

AlphaFold multimer modelling of the Syd LZII-RH2 dimer (Syd^359-526^) together with two Rab2 chains templated on the crystal structure of active GppNHp-bound Rab2 (PDB: 4rke) (Lardong et al., 2015) yielded a predicted structure or the Rab2:Syd^LZII-RH2^ complex (Fig. 2 H). It broadly resembles the crystal structures of Rab7 bound to the RILP RH2-domain (PDB: 1yhn) (Wu et al., 2005) and phospho-Rab8a bound to the RILPL2 RH2-domain (PDB: 6rir) (Waschbusch et al., 2020). In the AlphaFold prediction, the two Syd-RH2 monomers, each composed of two roughly antiparallel alpha helixes (Helix 1 and Helix 2), together form a four-helix bundle, with the N-terminal part of each Helix 1 constituting the main interaction surface with Rab2 (Fig. 2 H). Alanine substitution of the highly conserved residues L465 and R468 (Fig. S2 C) in Helix α1 that were predicted to form contacts with the Rab2 switch regions (Fig. 2 H) substantially reduced Syd-N2 precipitation by Rab2^Q65L^ (Fig. 2 I). These results were recapitulated by molecular dynamics (MD) simulations of the Rab2:Syd^LZII-RH2^ AlphaFold structure, which predict that the R468 residue significantly contributes to Rab2:Syd-RH2–binding energy (Fig. 2 J). In addition, mutation of a cluster of nine conserved residues in Helix 2 (Syd^7A^-N2) (Fig. S2, C and D) or deletion of the entire Helix 2 together with the seven most C-terminal residues of Helix α1 (Syd^1-485^, Syd-N4) (Fig. 2 F) weakened and entirely abolished Syd-N2 precipitation by Rab2^Q65L^, respectively. Together with MD modelling predicting that removal of Helix 2 would result in a substantial decrease in binding energy (Fig. 2 J), these data indicate that Helix 2, like Helix 1, plays an important role in Rab2 binding, perhaps by stabilizing the Helix 1 dimer conformation.

A critical early step during the JIP3-assisted assembly and activation of the dynein–dynactin complex is binding of the dynein light intermediate chain (DLIC) to the JIP3 RH1 domain (Singh et al., 2024). We confirmed that the Syd–dynein interaction is conserved by showing that Syd-N2 can coprecipitate *Drosophila* DLIC (Fig. 2 K). Moreover, further truncated Syd variants lacking the RH2 Helix 2 (Syd-N4) or containing only the RH1-LZI region (Syd^1-198^, Syd-N5) precipitated DLIC noticeably better than Syd-N2 (Fig. 2 K). These data mirror recent findings showing that the JIP3 RH1 domain is autoinhibited by a conserved motif in the RH2 domain (Singh et al., 2024).

Collectively, these data suggest that active Rab2 interacts with Syd via the cargo-binding Syd RH2 domain and that this interaction is compatible with kinesin-1 and dynein recruitment by Syd.

### Loss of Syd and Rab2 produce similar effects on axonal transport of DCVs and lysosomes

**Figure 3.**
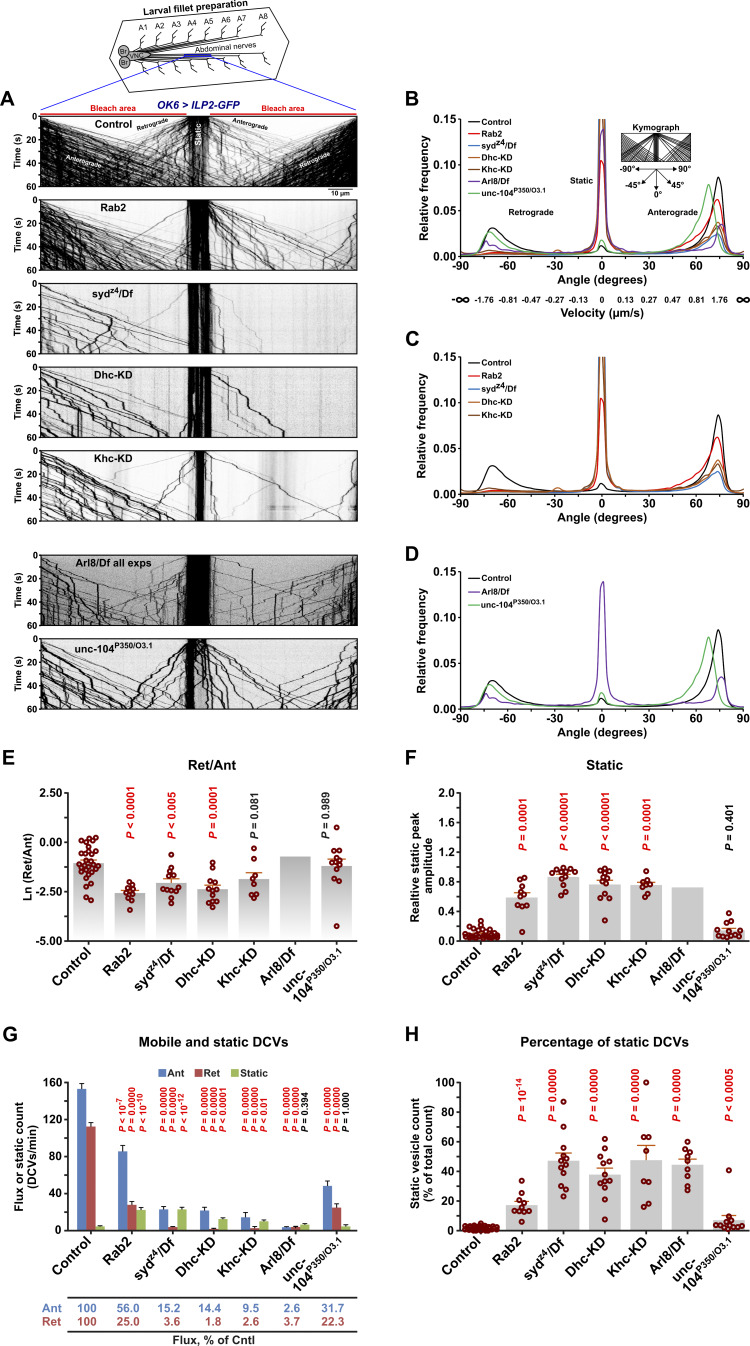
**Disruption of Rab2, Syd, and dynein result in similar DCV axonal transport phenotypes. (A)** Representative kymographs showing transport of ILP2-GFP–positive DCVs in motor axons in the A7 nerve of third instar larvae with the indicated genotypes. KD, motor neuron-specific knockdown (driven by *OK6*-Gal4). Time-lapse confocal imaging was performed immediately after bleaching the areas indicated with red bars. Each kymograph depicts a single recording, except for *Arl8/Df*, where 20 superimposed recordings from nine larvae are shown (see Materials and methods). Scale bar: 10 µm. **(B–D)** Directional distributions showing the relative frequency of DCV transport velocities, expressed as the angle between the DCV trajectories and the vertical axis in the kymographs in A (see inset in B). Actual DCV velocities converted from angles have been added to the x axis in B. For each genotype except *Arl8/Df*, the directional distribution was averaged from *n* larvae, where *n* is equal to the number of data points in E–H (specified below). The directional distribution of *Arl8/Df* was produced from the superimposed *Arl8/Df* recordings in A. **(E)** Logarithmic ratio of the retrograde to anterograde peak amplitude in the directional distributions in B–D (the retrograde and anterograde peak amplitude are the maximal relative frequency of angles lower than −46° and higher than 46°, respectively). **(F)** The static peak amplitude relative to the sum of the static, retrograde, and anterograde peak amplitudes (the static peak amplitude is the maximal relative frequency of angles between −13° and 13°, located centrally on the x axis). **(G)** Counts of DCVs entering from the sides into the field of view in the anterograde or retrograde directions and of static vesicles in the central unbleached area. Counts were done over 30 s and multiplied by two, converting the dynamic vesicle counts to DCV flux in vesicles per minute. **(H)** Percentage of static vesicle counts relative to total vesicle counts for each genotype in G. Results involving *Arl8/Df* represent reanalysis of data published earlier (Lund et al., 2021). ANOVA followed by Dunnett’s test (E, G, and H), Steel with control test (F). Number of larvae analyzed (*n*) in B–D and E–H: control 29, *Rab2* 10, *syd*^*z4*^*/Df* 12, *Dhc-KD* 12, *Khc-KD* 8, *Arl8/Df* 9, and *unc-104*^*P350*^*/*^*O3.1*^ 12.

Loss of Syd causes a strong defect in the axonal transport of SV proteins in *Drosophila* larvae (Bowman et al., 2000). If Syd constitutes the Rab2-dependent link between DCVs and molecular motors, one would expect disruption of Syd and Rab2 to produce similar effects on DCV transport. Using confocal imaging, we therefore examined mid-axon transport of ILP2-GFP–positive DCVs in L3 larval motor neurons targeted by the OK6-Gal4 driver (Lund et al., 2021). Specifically, during time-lapse imaging of a 130 µm long stretch of the A7 peripheral nerve in fillet-dissected larvae, we photobleached two 60 μm long flanking segments around a 10 μm central region of the nerve and then recorded the movement of fluorescent DCVs initially located in the unbleached center as well as those entering laterally from outside the field of view (Fig. 3 A). This approach allows the examination of both the transport in the anterograde and retrograde directions (distinguishable because motor neuron axons are uniformly oriented toward the periphery) and the abundance of static vesicles. After converting the time-lapse movies of DCV transport to kymographs, we plotted the frequency distribution of the angle between vesicle trajectories and the vertical axis in the kymographs, using a fast Fourier transform (see Materials and methods). The resulting “directional distributions” (Fig. 3, B–D) are amenable to high-throughput analysis and provide a convenient overview of the relative amounts of anterograde and retrograde transport (left- and rightmost peaks, respectively) and the relative amount of static cargo (middle peak at 0°). The relative amplitudes of the anterograde/retrograde and static peaks in the directional distributions (Fig. 3, B–F) aligned well with absolute vesicle flux and static vesicle counts across the genotypes examined (Fig. 3, G and H).

**Figure S3.**
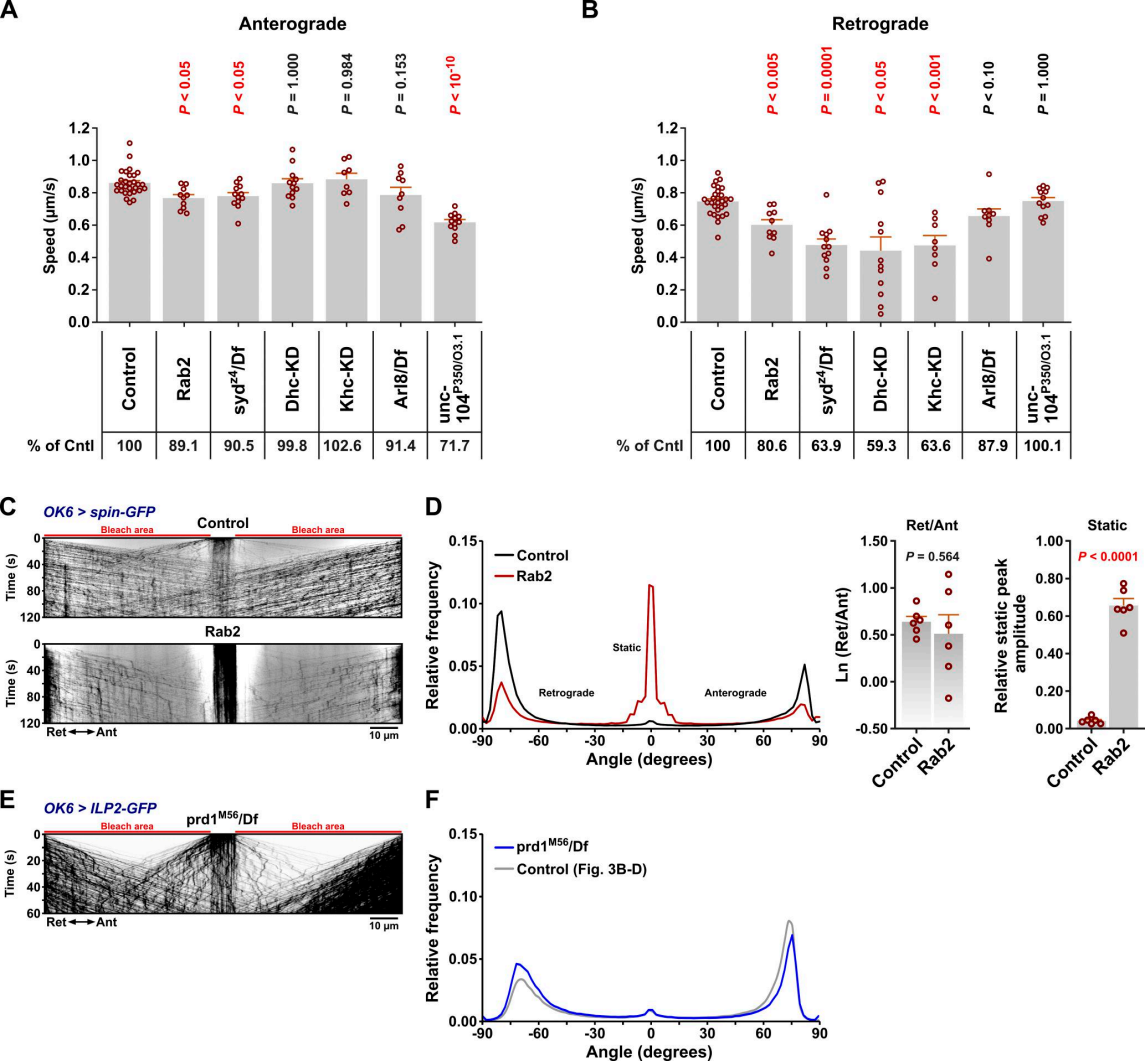
**Axonal transport speeds calculated from data in** Fig. 3 **, the defect in lysosomal transport of spin-GFP positive lysosomes in** ***Rab2*** **null larvae, and apparently normal axonal DCV transport in** ***prd1*** **null larvae.** **(A and B)** Anterograde (A) and retrograde (B) DCV movement speeds from the experiments in Fig. 3 (*OK6 > ILP2-GFP*). Number of larvae analyzed: control 29, *Rab2* 10, *syd*^*z4*^*/Df* 12, *Dhc-KD* 12, *Khc-KD* 8, *Arl8/Df* 9, and *unc-104P350/O3.1* 12. **(C)** Representative kymographs showing transport of Spinster-positive organelles in motor axons (*OK6 > Spinster-GFP*) in control and *Rab2* larvae. Scale bar: 10 µm. **(D)** Left, directional distributions derived from C, averaged from six control and six *Rab2* larvae. Right, the logarithmic ratio of retrograde to anterograde peak amplitude and the relative static peak amplitude for the directional distributions at the left. **(E)** Representative kymograph showing DCV transport in motor axons of *prd1*^*M56*^*/Df* larvae. Scale bar: 10 µm. **(F)** Directional distribution derived from E, averaged from four *prd1*^*M56*^*/Df* larvae (blue curve), shown together with a replica of the directional distribution of control larvae in Fig. 3, B–D (gray curve). *Arl8/Df* results in A and B, and the results in C and D represent reanalysis of data published earlier (Lund et al., 2021). Bar graphs in A, B, and D, right represent mean + SEM. ANOVA followed by Dunnett’s test (A), Steel with control test (B), Student’s *t* test (D, right).

**Figure 4.**
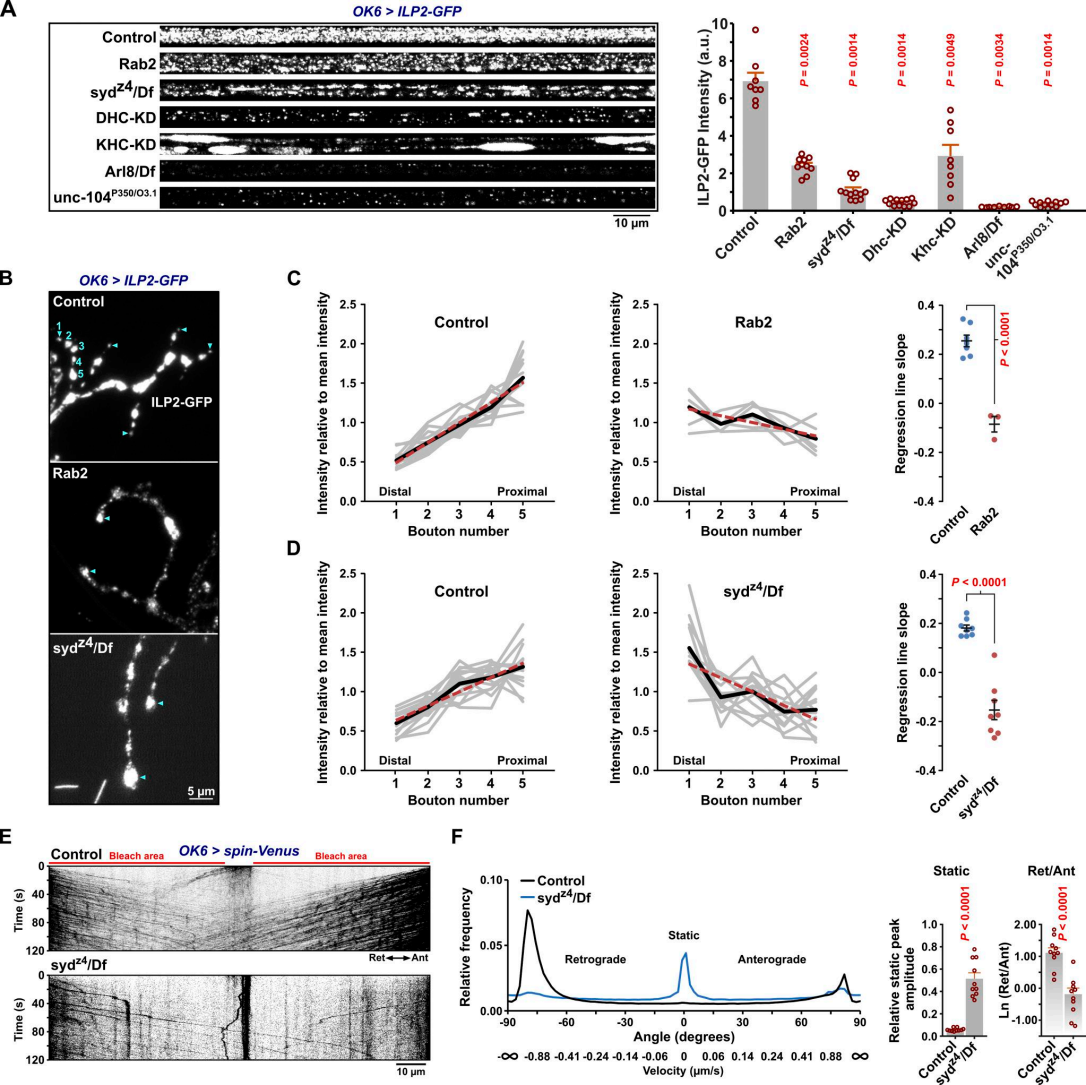
**Axonal levels and distribution of DCV cargo and disrupted transport of lysosomal organelles in *syd* mutants. (A)** Left, representative pre-bleach confocal micrographs of the A7 nerve in third instar larvae expressing ILP2-GFP in motor neurons. Right, quantification of the axonal ILP2-GFP signal intensity. a.u., arbitrary units. Scale bar: 10 µm. Number of larvae analyzed: control 8, *Rab2* 10, *syd*^*z4*^*/Df* 12, *Dhc-KD* 12, *Khc-KD* 8, *Arl8/Df* 9, and *unc-104*^*P350*^*/*^*O3.1*^ 12. **(B)** Neuromuscular junction of muscle fibers 6 and 7 in control, *Rab2*, and *syd*^*z4*^*/Df* larval fillets. Numbers in blue indicate the distal five boutons in a single branch of a motor neuron ending. Blue triangles indicate the most distal bouton in the same branch and other branches as well. Scale bar: 5 µm. **(C and D)** Left and middle, ILP2-GFP signal intensity in the distal five boutons of individual branches. Thick black lines represent the mean intensity for each bouton number. Dashed red lines were produced by linear regression. Right, regression line slopes. The mean ± SEM is indicated. Number of terminals (larvae) analyzed: control 13 (7), *Rab2* 6 (3) in C; control 16 (8), *syd*^*z4*^*/Df* 14 (8) in D. **(E)** Representative kymographs showing transport of Spinster-positive organelles in A7 motor axons of control and *syd*^*z4*^*/Df* third instar larvae. Scale bar: 10 µm. **(F)** Left, directional distributions of Spinster-positive organelle transport velocities, expressed as angles, cf. Fig. 3, B–D. Actual velocities converted from angles have been added to the x axis. For both control and *syd*^*z4*^*/Df*, the average directional distribution from 10 larvae is shown. Right, the relative static peak amplitude and the logarithmic ratio of retrograde to anterograde peak amplitude for the directional distributions at the left. Bar graphs in F represent the mean + SEM. Steel with control test (A, right), Student’s *t* test (right of C, D, and F).

In WT animals, apart from a smaller static component, we observed large anterograde and retrograde DCV fluxes with a moderate excess of anterograde transport (Fig. 3, A, B–E, and G), consistent with axonal DCV circulation. As reported previously (Lund et al., 2021), loss of Rab2 was associated with a pronounced DCV transport defect with a relative increase in the static vesicle signal, a moderate reduction in anterograde transport, and a disproportionately severe reduction in retrograde transport (Fig 3, A–C and E–H; and Video 1). The latter was evidenced by the almost complete disappearance of the sharp peak associated with retrograde transport in the directional distribution (Fig. 3, B and C). Importantly, a qualitatively similar but more severe phenotype was observed in larvae hemizygous for the *syd*^*z4*^ strong loss-of-function allele (Bowman et al., 2000) (*syd*^*z4*^*/Df*), or when dynein function was impaired in motor neurons by RNAi-mediated depletion of the dynein heavy chain (Dhc) (Fig 3, A–C and E–H; and Video 1). The speed of the remaining retrograde vesicles in *Rab2* null and *syd*^*z4*^*/Df* animals was also considerably slower compared with WT, similar to Dhc-depleted animals (Fig. S3 B). Suppression of the kinesin-1 motor by kinesin-1 heavy chain (Khc) depletion also resulted in a transport defect characterized by a selective deficit in retrograde DCV traffic (Fig. 3, A–C and E–H; Fig. S3, A and B; and Video 1) and featuring the appearance of prominent axonal DCV-filled focal accumulations (Fig. 4 A). The selective effect of kinesin-1 dysfunction on the retrograde DCV flux in flies has been reported previously (Lim et al., 2017) and may be due to progressive stalling during anterograde transport, although there are also indications of direct and indirect codependence between kinesin-1 and dynein (Arimoto et al., 2011; Twelvetrees et al., 2016). These findings suggest that Rab2 and Syd are involved in the function of dynein and/or kinesin-1 during DCV transport.

**Video 1.** **Montage showing time-lapse imaging of DCV transport in axons in the A7 nerve of live fillet preparations of third instar *OK6* > ILP2-GFP larvae with the indicated background genotypes.** The first video frame is a pre-bleach image, while the subsequent frames shows DCV transport after photobleaching of the nerve, sparing the ∼10 µm wide central region. Ant, anterograde; Ret, retrograde. Scale bar: 10 µm.

In contrast, larvae carrying a heteroallelic combination of the *unc-104*^*P350*^ null and *unc-104*^*O3.1*^ hypomorphic mutations of the fast anterograde kinesin-3 family Unc-104 motor (ortholog of mammalian KIF1A/B/C) displayed a qualitatively different axonal transport phenotype characterized by a severe reduction in axonal DCV content (Fig. 4 A) due to a failure of cell body exit (Barkus et al., 2008), combined with more symmetrical bidirectional fluxes of remaining axonal DCVs (Fig. 3, A, B, and D–H; and Video 1). *unc-104*^*P350*^*/unc-104*^*O3.1*^ animals also displayed a strong reduction in the mean anterograde vesicle speed (Fig. S3 A), reflected in a pronounced leftward shift (toward lower velocities) of the anterograde peak in the directional distribution (Fig. 3, B and D). This is consistent with Unc-104 being responsible for fast anterograde DCV movement (Barkus et al., 2008; Lim et al., 2017). Applying the same analysis to previously published (Lund et al., 2021) DCV axonal transport recordings in animals lacking Arl8, thought to be responsible for Unc-104 activation (Guardia et al., 2016; Niwa et al., 2016; Vukoja et al., 2018), also showed a similar phenotype with very strong but symmetrical reductions in the bidirectional DCV flux, but without the characteristic reduction in anterograde speed (Fig. 3, A, B, and D–H; and Fig. S3 A). Interestingly, unlike *unc-104* mutants, but resembling Rab2, Syd, Dhc, and kinesin-1–deficient animals, *Arl8* nulls also displayed a large relative increase in the static DCV component, suggesting that it may also be involved in regulation of kinesin-1 and dynein motors (Fig. 3, A, B, and D–H).

Disruption of dynein function causes a pronounced accumulation of excess DCVs in the distal-most boutons of larval motor terminals (Wong et al., 2012). Consistent with this observation, in type Ib motor terminals on larval muscles 6 and 7, both *syd* and *Rab2* mutants displayed a clear reversal of the usual trend of decreasing bouton content of ILP2-GFP in more distal boutons (Fig. 4, B–D), although the Syd phenotype was again more severe. This further indicates that Rab2 and Syd are required for retrograde dynein-dependent transport.

Besides DCV transport, Rab2 is required for axonal transport of lysosomes and early/late endosomes in flies (Lund et al., 2021). JIP3/4 are also well known to mediate lysosomal motility in mammals (Cason and Holzbaur, 2023; Drerup and Nechiporuk, 2013; Gowrishankar et al., 2021; Kluss et al., 2022). We found that transport of lysosomes labelled with Spinster-Venus was severely disrupted in motor axons of *syd*^*z4*^*/Df* mutant larvae, with less bidirectional transport and a relative increase in static organelles (Fig. 4, E and F). While direct comparisons with previously recorded data for *Rab2* nulls (Fig. S3, C and D) (Lund et al., 2021) are difficult due to the use of different markers (Spinster-Venus vs. Spinster-GFP, necessitated by the different chromosomal locations of *Rab2* and *syd*), the disruption of lysosomal transport appeared to be similar in *Rab2* and *syd* mutants, albeit with a stronger defect in *syd*^*z4*^*/Df*.

In conclusion, loss of Rab2 and Syd produces qualitatively similar axonal transport defects for DCVs and lysosomes, although the Syd mutant phenotype is more severe. Moreover, the Rab2 and Syd-related DCV transport defect is consistent with a disruption of dynein-mediated retrograde motility.

### The Arl8 effector RUFY cooperates with Syd to drive retrograde axonal transport of DCVs

**Figure S4.**
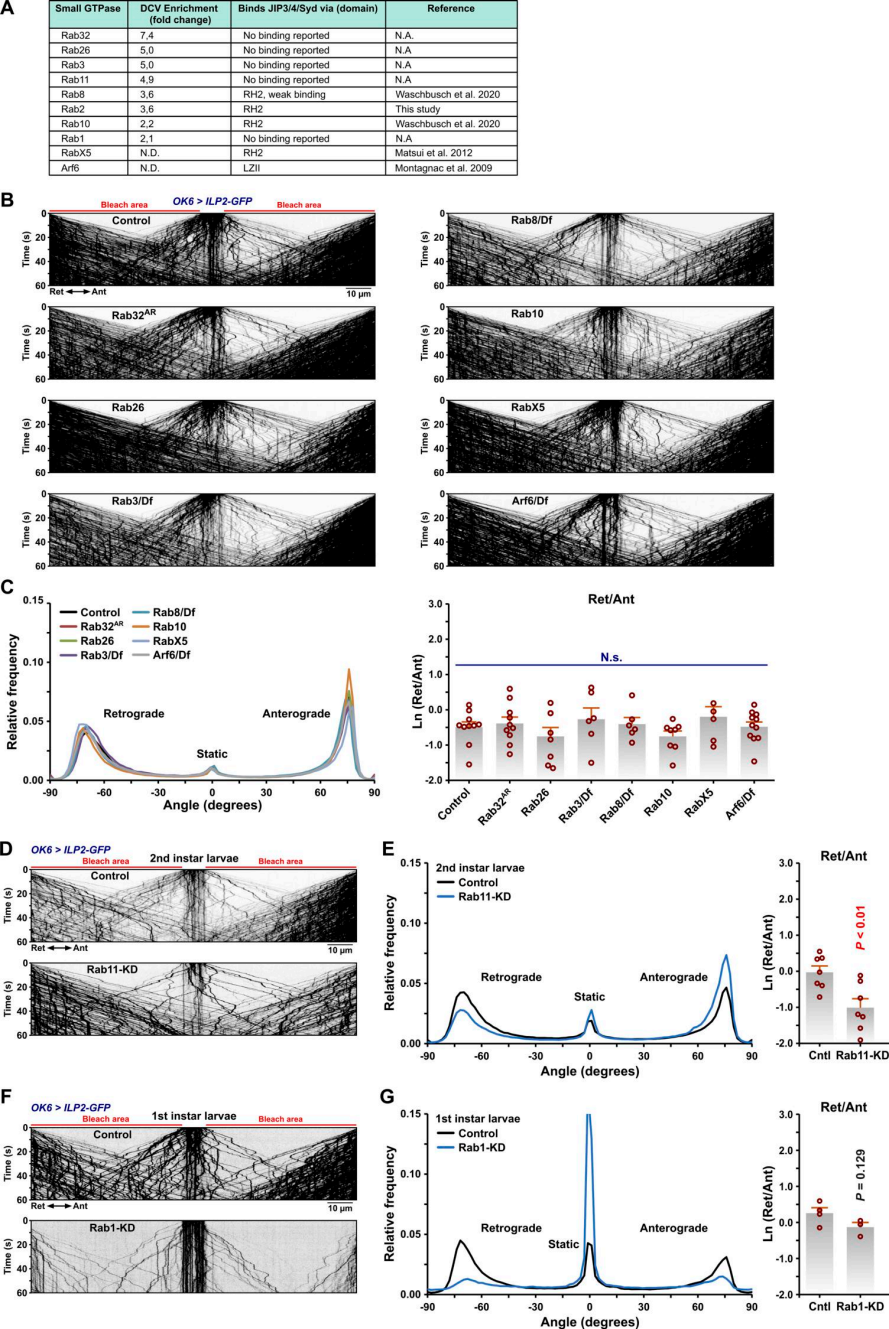
**Effect of disrupting different Rab and Arf GTPases on axonal DCV transport. (A)** Small GTPases enriched in the VMAT-specific PB dataset or known to bind JIP3/4/Syd. **(B)** Representative kymographs showing transport of ILP2-GFP–positive DCVs in third instar larval motor axons in controls, the indicated Rab GTPase mutants, and *Arf6/Df*. Scale bar: 10 µm. **(C)** Left, directional distributions derived from B, averaged from the following number of larvae: control 10, *Rab32*^*AR*^ 10, *Rab26* 8, *Rab3/Df* 6, *Rab8/Df* 6, *Rab10* 8, *RabX5* 6, and *Arf6/Df* 11. Right, the logarithmic ratio of retrograde to anterograde peak amplitude for the directional distributions at the left. N.s., not significant (ANOVA, P = 0.522). **(D)** Representative kymographs showing transport of ILP2-GFP–positive DCVs in motor axons in control larvae and larvae subjected to motor neuron-specific knockdown of *Rab11*. DCV transport was recorded in second instar larvae for both genotypes. Scale bar: 10 µm. **(E)** Left, directional distributions derived from D, averaged from seven control and seven *Rab11-KD* larvae. Right, the logarithmic ratio of retrograde to anterograde peak amplitude for the directional distributions at the left. **(F)** Representative kymographs showing transport of ILP2-GFP–positive DCVs in motor axons in control larvae and larvae subjected to motor neuron-specific knockdown of *Rab1*. DCV transport was recorded in first instar larvae for both genotypes. Scale bar: 10 µm. **(G)** Left, directional distributions derived from F, averaged from four control and three *Rab1-KD* larvae*.* Right, the logarithmic ratio of retrograde to anterograde peak amplitude for the directional distributions at the left. Bar graphs in C, E, and G represent mean + SEM. ANOVA (C, right), Student’s *t* test (E, right; G, right).

**Figure S5.**
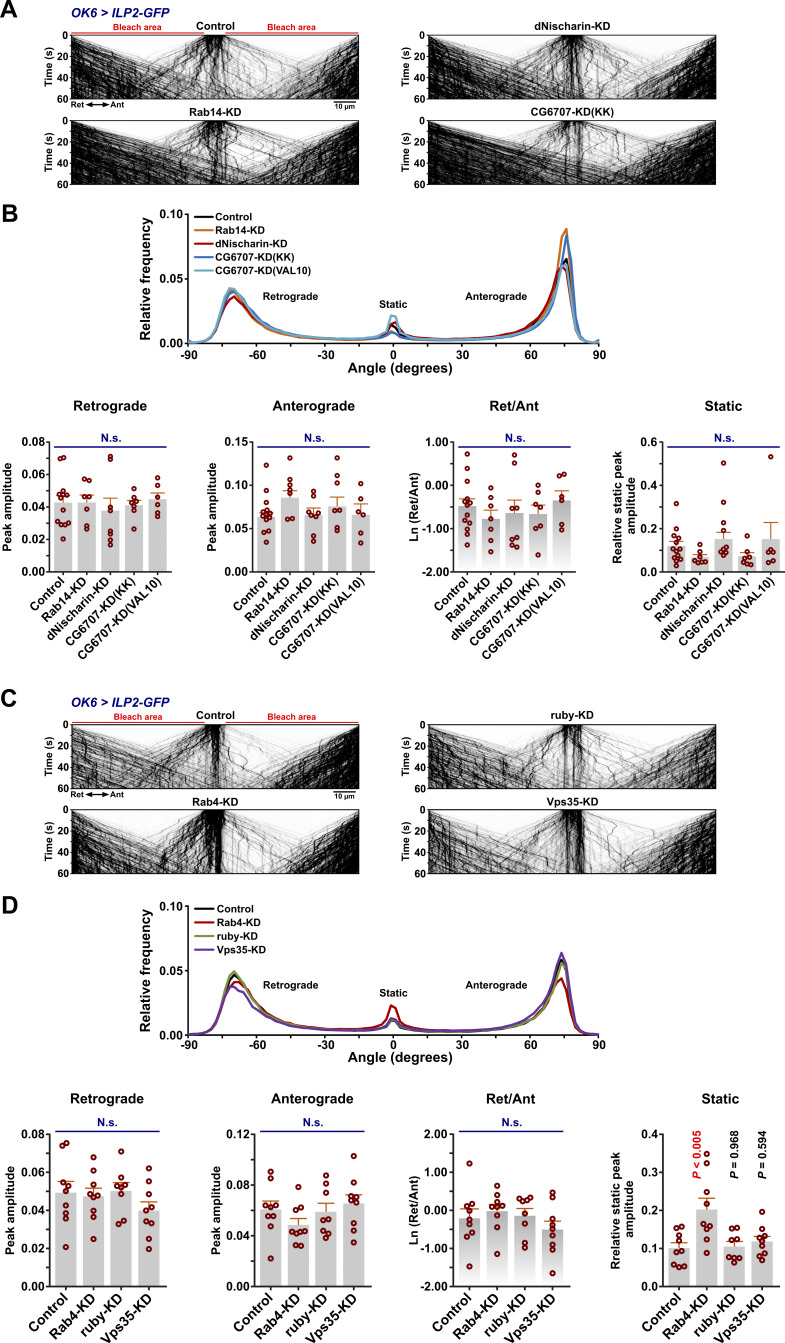
**Effect on axonal DCV transport of RNAi knockdown of different genes involved in trafficking of DCV membrane proteins or suspected to play a role in motor adaptor recruitment. (A)** Representative kymographs showing transport of ILP2-GFP–positive DCVs in motor axons of control larvae and larvae subjected to motor neuron-targeted knockdown of *Rab14*, *dNischarin*, or *CG6707*. Scale bar: 10 µm. **(B)** Top, directional distributions derived from A, averaged from the following number of larvae: control 13, *Rab14-KD* 7, *dNischarin-KD* 8, *CG6707(KK)-KD* 7, and *CG6707(VAL10)-KD* 6. Bottom, the retrograde peak amplitude (ANOVA, P = 0.917), anterograde peak amplitude (ANOVA, P = 0.150), logarithmic ratio of retrograde to anterograde peak amplitude (ANOVA, P = 0.738), and relative static peak amplitude (ANOVA, P = 0.089) for the directional distributions at the top. In A and B, KK and VAL20 refer to the use of *UAS-RNAi* lines from the KK collection and the VALIUM10 vector-based collection, respectively. For simplicity, only KK line data are illustrated in A. **(C)** Representative kymographs showing transport of ILP2-GFP–positive DCVs in motor axons of control larvae and larvae subjected to motor neuron-targeted knockdown of *Rab4*, *ruby*, or *Vps35*. Scale bar: 10 µm. **(D)** Top, directional distributions derived from C, averaged from the following number of larvae: control 9, *Rab4-KD* 9, *ruby-KD* 8, and *Vps35-KD* 9. Bottom, the retrograde peak amplitude (ANOVA, P = 0.431), anterograde peak amplitude (ANOVA, P = 0.298), logarithmic ratio of retrograde to anterograde peak amplitude (ANOVA, P = 0.415), and relative static peak amplitude (ANOVA, P <0.005, followed by Dunnett’s test obtaining the indicated P values). Data in all panels are from third instar larvae. Bar graphs in B and D represent mean + SEM. N.s., not significant.

The less severe *Rab2* null DCV transport phenotype compared with the *syd* mutant phenotype strongly suggests the presence of additional vesicular Syd-recruitment factors. Syd/JIP3/4 proteins bind small GTPases Arf6 (Montagnac et al., 2009), Rab36, Rab8, and LRRK-phosphorylated Rab10 (Matsui et al., 2012; Waschbusch et al., 2020), of which Rab8 and Rab10 were enriched in our VMAT-specific PB dataset (Fig. S4 A and Data S3). Moreover, Arf6 and Rab10 control JIP3/4-mediated axonal transport of mammalian autolysosomes (Boecker et al., 2021; Cason and Holzbaur, 2023). We tested larvae with null mutations in Arf6, Rab8, and Rab10 or homozygous for a transposon insertion allele for the fly Rab36 ortholog, RabX5, but found no obvious disruption of axonal DCV transport (Fig. S4, B and C). Of multiple Rabs (besides Rab2) enriched in VMAT-proximity proteomics (Fig. S4 A), only Rab1 or Rab11 produced any effect on axonal DCV transport when disrupted by mutation or motor neuron-specific depletion (Fig. S4, B–G). However, since no physical interactions between Rab1 or Rab11 and Syd family proteins have been reported, and the Rab1- and Rab11-depleted animals did not develop beyond late first or early second instar, we did not pursue this line of inquiry further. We also tested the ortholog of the mammalian TMEM55A/B transmembrane proteins (CG6707), which ranked high in both Rab2- and VMAT-PB datasets (Fig. 1 G). TMEM55B mediates the recruitment of JIP4 for dynein-mediated lysosomal motility in mammals (Willett et al., 2017). However, depletion of CG6707 with two independent RNAi transgenes did not affect axonal transport of DCVs (Fig. S5, A and B).

**Figure 5.**
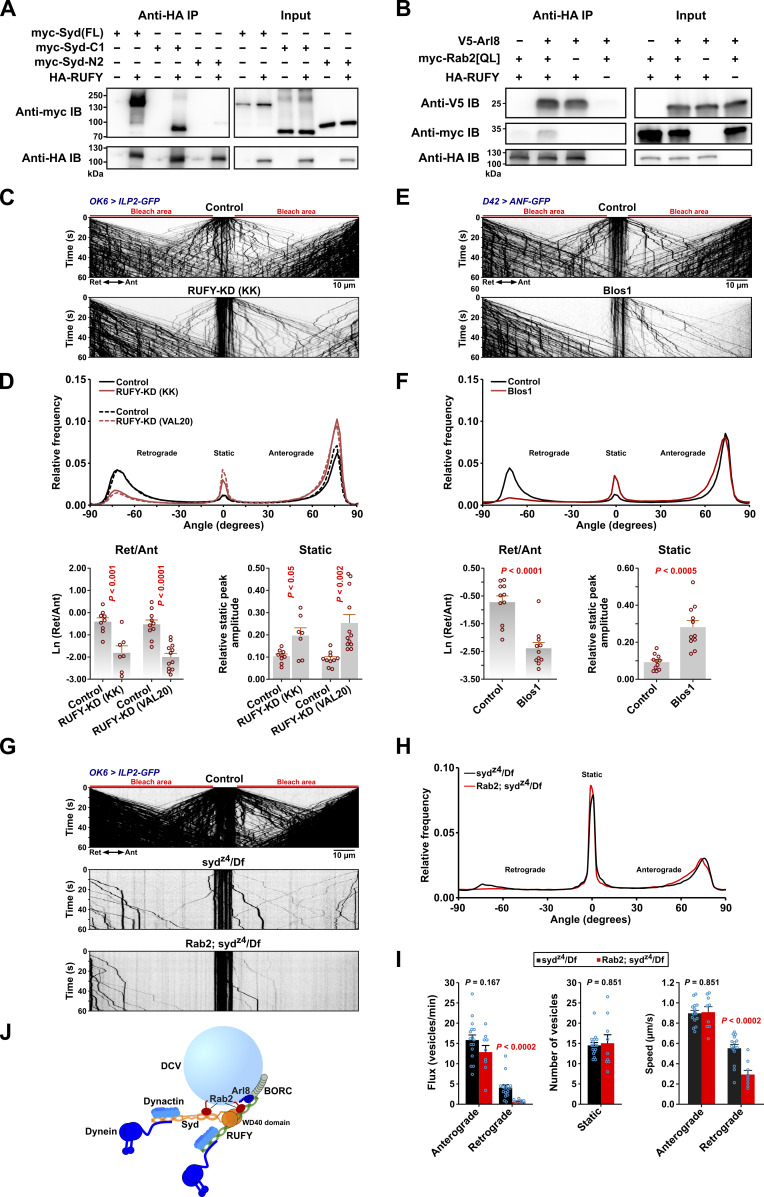
**RUFY interacts with Syd, Arl8, and Rab2 and is required for axonal DCV transport. (A and B)** Western blots of the indicated Co-IP eluates (∼40% eluate volume) and HEK cell lysates (∼1% reaction volume). A, HA-tagged RUFY co-immunoprecipitates myc-tagged full-length Syd and truncated Syd-C1 (Syd^530-1226^) containing only the C-terminal WD40 domain but not truncated Syd-N2 (Syd^1-529^) that lacks the WD40 domain. B, HA-RUFY co-immunoprecipitates V5-tagged Arl8, and co-expression of V5-Arl8 enhances Co-IP of myc-Rab2^Q65L^ with HA-RUFY. **(C)** Representative kymographs showing transport of ILP2-GFP–positive DCVs in motor axons of control larvae and larvae subjected to motor neuron-specific knockdown of *RUFY*, driven by *OK6*-Gal4. Scale bar: 10 µm. **(D)** Top, directional distributions derived from C, averaged from the following number of larvae: control for *RUFY-KD(KK)* 9, *RUFY-KD(KK)* 7; control for *RUFY-KD(VAL20)* 10, *RUFY-KD(VAL20)* 12. Bottom, the logarithmic ratio of retrograde to anterograde peak amplitude and the relative static peak amplitude for the directional distributions at the top. In C and D, “KK” and “VAL20” refer to *UAS-RNAi* lines from the KK collection and the VALIUM20 vector-based collection, respectively. For simplicity, only KK line data are illustrated in C. **(E)** Representative kymographs showing transport of ANF-GFP–positive DCVs in motor axons of control and *Blos1* larvae. Scale bar: 10 µm. **(F)** Top, directional distributions derived from E. Averages from 11 larvae are shown for both Control and *Blos1*. Bottom, the logarithmic ratio of retrograde to anterograde peak amplitude and the relative static peak amplitude for the directional distributions at the top. **(G)** Kymographs of transport of ILP2-GFP–positive DCVs in motor axons of control, *syd*^*z4*^*/Df* single mutant, and *Rab2; syd*^*z4*^*/Df* double mutant larvae. Scale bar: 10 µm. **(H)** Directional distributions derived from G, averaged from 16 *syd*^*z4*^*/Df* and 9 *Rab2; syd*^*z4*^*/Df* larvae. **(I)** Flux of dynamic vesicles, counts of static vesicles in the central unbleached region, and speed of dynamic vesicles in the *syd*^*z4*^*/Df* and *Rab2; syd*^*z4*^*/Df* mutants also shown in H. **(J)** Hypothetical model of the DCV dynein–dynactin recruitment complex, consisting of Syd and RUFY anchored to the vesicle by Rab2 and Arl8. Kinesin-1 bound by Syd and kinesin-3 regulated by Arl8-BORC are omitted. It is uncertain if RUFY can recruit and activate dynein–dynactin. Bar graphs in D, F, and I represent the mean + SEM and were analyzed with Student’s *t* test. For conversion of angles in D, F, and H to DCV velocities, see Fig. 3 B. All data are from third instar larvae. Results in E and F represent reanalysis of data published earlier (Lund et al., 2021). Source data are available for this figure: SourceData F5.

In our search for more components of the retrograde motor complex, we next focused our attention on dNischarin and RUFY, which also ranked high in the Rab2-PB dataset but did not interact strongly with Rab2 (Fig. 1 B and Fig. 2 A). Interestingly, while motor neuron-specific dNischarin depletion produced no effect (Fig. S5, A and B), depletion of RUFY with two independent RNAi transgenes caused a pronounced DCV transport defect characterized by a selective reduction in retrograde movement and a relative increase in static DCV cargo (Fig. 5, C and D). As almost all retrograde DCV transport is already lost in *syd*^*z4*^*/Df* animals (Fig. 3, A–C, and G), we reasoned that Syd and RUFY function as part of the same mechanism to recruit/activate dynein. Although mammalian RUFY proteins have been proposed to function as dynein-activating adaptors in their own right (Keren-Kaplan et al., 2022; Rawat et al., 2023), at least one, RUFY3, also binds JIP4 (Kumar et al., 2022). Consistent with this observation, we found that fly RUFY immunoprecipitates Syd when the two proteins are expressed together in HEK cells. This interaction appears to depend on the Syd C-terminal WD40 domain, as the Syd-C1 (Syd^530-1226^) region containing it is both required and sufficient for a strong binding to RUFY (Fig. 5 A). Similar to its mammalian orthologs, which are ARL8A/B effectors (Keren-Kaplan et al., 2022; Kumar et al., 2022; Rawat et al., 2023), RUFY also bound fly Arl8 in Co-IP experiments (Fig. 5 B). The presence of Arl8 also increased the interaction between RUFY and Rab2^Q65L^, similar to the reported effect of Arl8b on the interaction between RUFY1 and the Rab2-related (Haga and Fukuda, 2025) Rab14 GTPase in mammals (Rawat et al., 2023) (Fig. 5 B). This suggests that Syd may be recruited to the DCV surface by a combination of interactions, including Rab2-binding via the RH2 domain and WD40 domain-dependent binding to RUFY, which itself is recruited to the vesicles via Arl8 and possibly Rab2 (Fig. 5 J).

This model fits with our observations that *Arl8* nulls, besides showing a relatively symmetrical reduction in the extent of anterograde and retrograde transport due to a failure of cell body exit (Lund et al., 2021), also display a strong increase in the proportion of static DCVs (Fig. 3, A, B, and D–H) similar to the syd/dynein/kinesin-1 phenotypic group. An involvement of Arl8 in dynein regulation via RUFY can also explain our previously published data (Lund et al., 2021) showing that knockout of the critical BORC subunit, Blos1 (Boda et al., 2019; Pu et al., 2015), produces a strikingly selective loss of retrograde axonal transport of ANF-positive DCVs (reanalyzed in Fig. 5, E and F).

In addition to binding Arl8, RUFY proteins are also known as Rab effectors; fly RUFY binds active Rab4 (Gillingham et al., 2014), and mammalian RUFY1 requires Rab14 for recruitment to endosomes (Rawat et al., 2023). We observed that whereas Rab14 depletion did not affect DCV axonal transport, a small but significant increase in the static component resulted upon Rab4 depletion, suggesting that Rab4 may have a minor role in RUFY recruitment to DCVs (Fig. S5, C and D).

A requirement for the Syd:RUFY interaction potentially explains the strong phenotype of the *syd*^*z4*^ allele, which introduces a premature stop codon (at amino acid position 514 in isoform A), leading to the truncation of the entire Syd WD40 domain but leaving a protein roughly corresponding to our Syd-N2 construct (Bowman et al., 2000; Schulman et al., 2014) (Fig. 2 C), containing both the Rab2-binding RH2 domain and the dynein–dynactin-activating region as defined by Singh et al. (2024) for JIP3 (Singh et al., 2024). Interestingly, although almost all retrograde (and most anterograde) transport is lost in *syd*^*z4*^*/Df* hemizygous larvae (Fig. 3, A–C, and G), a small retrograde DCV flux remained (Fig. 5, G–I). This residual retrograde flux was almost entirely eliminated in *Rab2; syd*^*z4*^*/Df* double mutants, with the few remaining retrograde vesicles moving significantly slower, as would be expected if it was driven by lower affinity recruitment of the remaining Syd fragment by Rab2 alone (Fig. 5, G–I). However, we cannot exclude an alternative model where RUFY functions directly as the activating adaptor for dynein and is recruited to DCVs by a combination of Syd (via the WD40 domain), Rab2, and Arl8 (Fig. 5 J).

In conclusion, Syd and the Arl8 effector RUFY interact via the Syd C-terminal WD40 domain and together are required for retrograde axonal transport of DCVs. This implies that Arl8 and its activator, BORC, are also involved in retrograde dynein-mediated DCV transport, in addition to axonal entry and anterograde transport mediated by kinesin-3.

### JNK promotes axonal DCV motility

**Figure 6.**
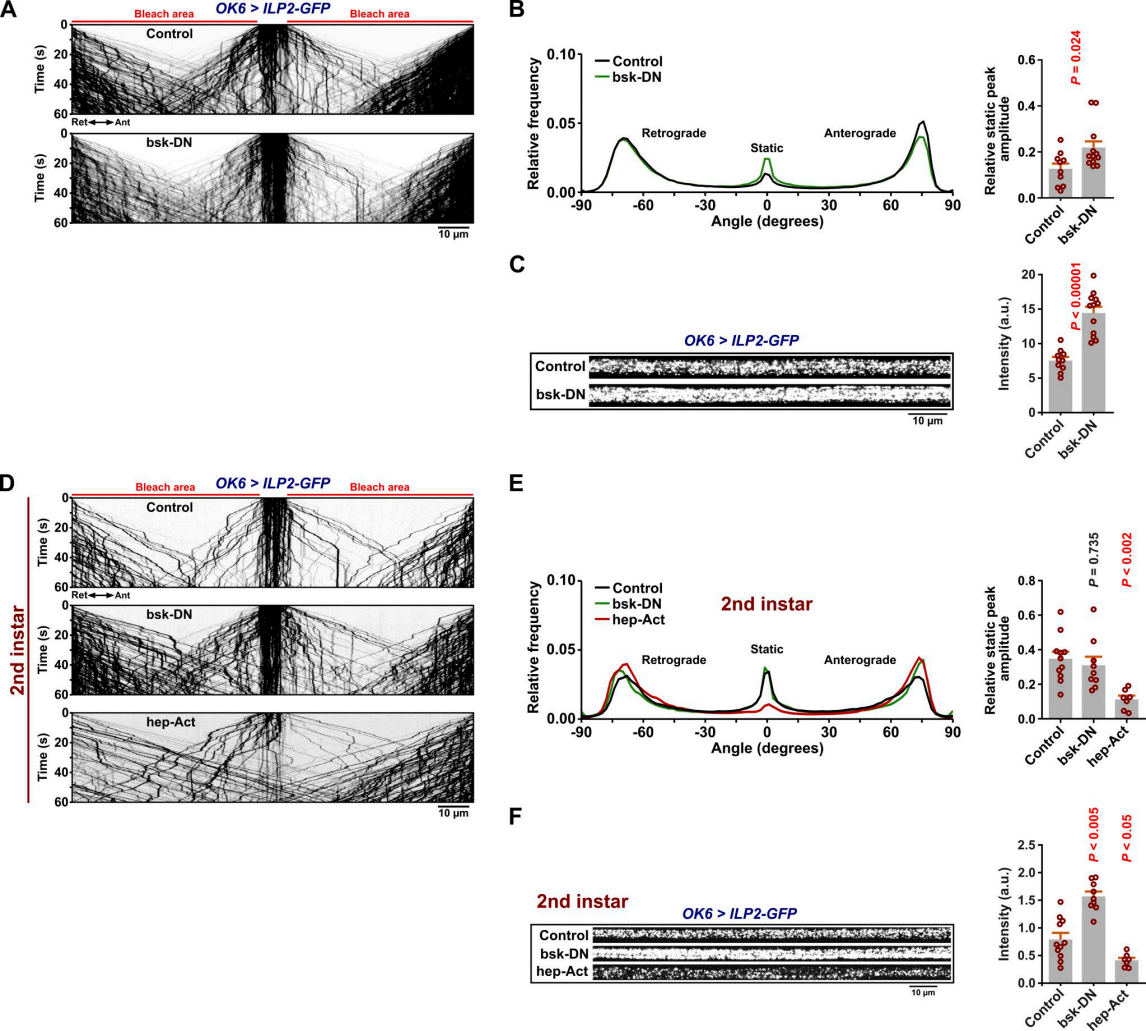
**Effects of altered JNK activity on DCV transport. (A)** Kymographs of DCV transport in motor axons of third instar control and *bsk-DN* larvae. Scale bar: 10 µm. **(B)** Left, directional distributions derived from A. Right, the relative static peak amplitude for the directional distributions at the left. **(C)** Axonal pre-bleach intensity of control and *bsk-DN* third instar larvae. Scale bar: 10 µm. Number of analyzed larvae in B and C: control 10, *bsk-DN* 12. **(D)** Kymographs of DCV transport in motor axons of second instar control, *bsk-DN*, and *hep-Act* larvae. Scale bar: 10 µm. **(E)** Directional distributions derived from D. Right, the relative static peak amplitude for the directional distributions at the left. **(F)** Axonal pre-bleach intensity of control, *bsk-DN*, and *hep-Act* second instar larvae. Scale bar: 10 µm. Number of analyzed larvae in E and F: control 11, *bsk-DN* 9, and *hep-Act* 7. Student’s *t* test (right of B, C, and E), Steel with control test (F, right).

Besides the vesicular motor adaptor role, Syd/JIP3/4 proteins function as important scaffolds and transport adaptors for the JNK MAP kinase (Byrd et al., 2001; Ito et al., 1999; Kelkar et al., 2000). Active JNK is important for axonogenesis and is enriched in mature axons (Oliva et al., 2006). Moreover, there are strong indications that JNK regulates Syd-linked motor activity or Syd-cargo binding (Byrd et al., 2001; Schulman et al., 2014). We therefore tested if the activity level of the sole fly JNK ortholog, basket (bsk), affects axonal DCV transport. Overexpression of dominant-negative bsk (*bsk-DN*) in larval motor neurons increased the relative amount of static axonal DCV signal, accompanied by a doubling of total axonal DCV marker content (Fig. 6, A–C), implying an increase in the total mid-axonal DCV population due to DCV stalling. As motor neuron overexpression of the constitutively active form of the upstream bsk activator hemipterous/MKK7 (hep-Act) blocked development before the L3 stage, we also examined DCV transport in hep-Act– and bsk-DN–expressing L2 larvae. Though at this early developmental stage bsk-DN did not result in an appreciable relative increase in static vesicles, hep-Act expression both reduced the relative amount of static vesicles and the total axonal ILP2 content (Fig. 6, D–F). These results suggest that a baseline level of JNK activity is required to ensure normal axonal DCV circulation.

**Figure S6.**
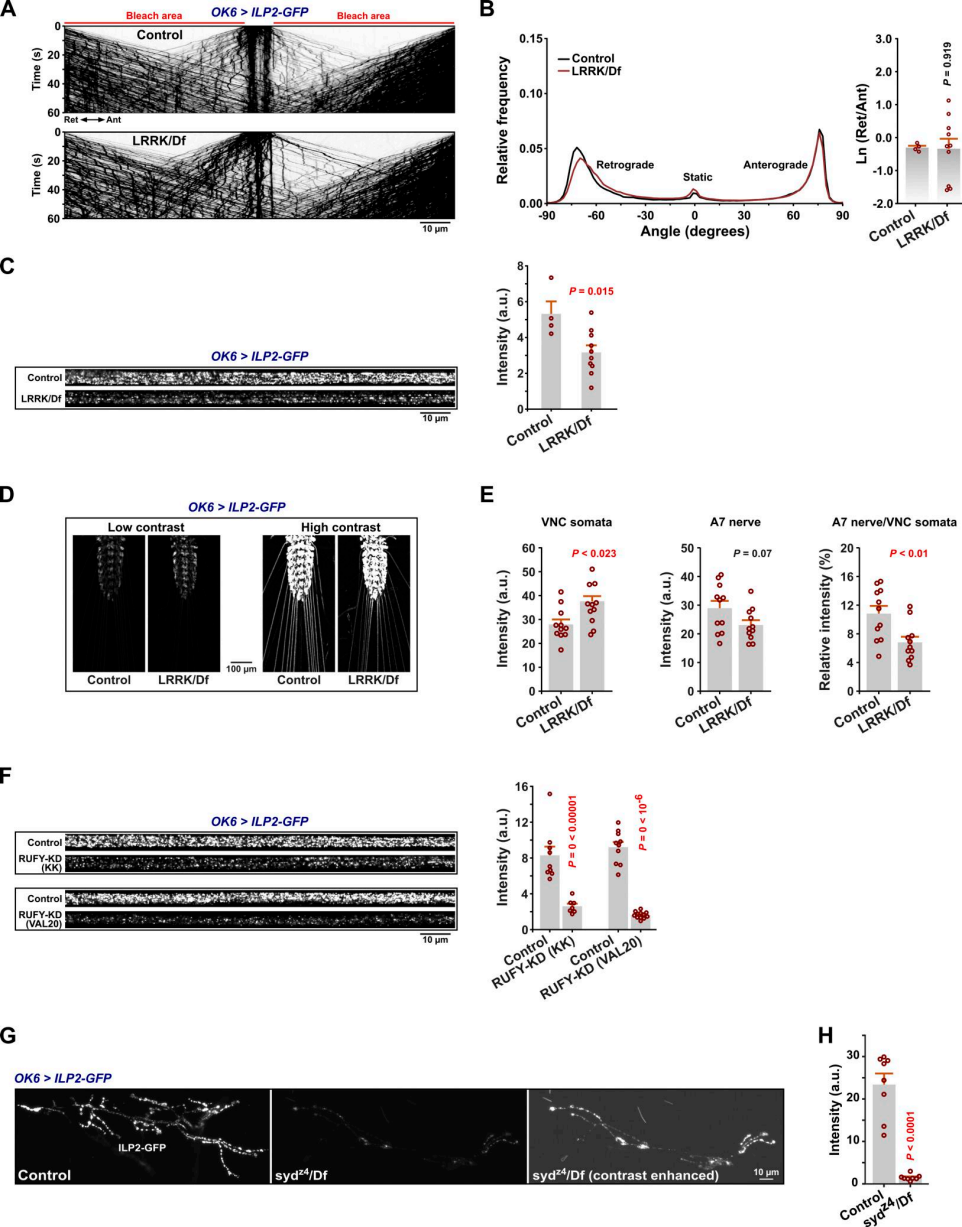
**Axonal transport and neuronal distribution of DCVs in *LRRK* null larvae, decreased peripheral nerve DCV content under *RUFY* RNAi knockdown, and decreased DCV content in *syd* mutant NMJs. (A)** Representative kymographs showing transport of ILP2-GFP–positive DCVs in motor axons of control and *LRRK/Df* larvae. Scale bar: 10 µm. **(B)** Left, directional distributions derived from A. Right, the logarithmic ratio of retrograde to anterograde peak amplitude for the directional distributions at the left. **(C)** Left: Representative confocal images showing the pre-bleach ILP2-GFP intensity in A7 nerves of control and *LRRK/Df* larvae. Scale bar: 10 µm. Right: Quantification of the ILP2-GFP signal intensity. Number of larvae analyzed in B and C: control 4, *LRRK/Df* 10. **(D)** Confocal images (sum projection of z-stacks) showing the ILP2-GFP signal in VNCs and peripheral nerves of third instar control and *LRRK/Df* larvae. Images are shown at both low contrast settings at which the VNC cell body signal is not saturated (left) and high contrast settings at which the axonal signal in peripheral nerves is visible (right). Scale bar: 100 µm. **(E)** Quantification of ILP2-GFP fluorescence in D from VNC cell bodies (left) and A7 peripheral nerves (middle), as well as the ratio between nerve and cell body fluorescence (right). Number of larvae analyzed: control 11, *LRRK/Df* 11. **(F)** Left: Pre-bleach ILP2-GFP intensity in A7 nerves of control larvae and larvae subjected to motor neuron-targeted knockdown of *RUFY* (KK or VALIUM20 *UAS-RNAi* lines, cf. Fig. 5). Scale bar: 10 µm. Right: Quantification of the ILP2-GFP signal intensity. Number of larvae analyzed: control for *RUFY-KD (KK)* 9, *RUFY-KD (KK)* 7; control for *RUFY-KD (VAL20)* 10, *RUFY-KD (VAL20)* 12. **(G)** Confocal images showing the intensity of the presynaptic ILP2-GFP signal in the neuromuscular junction of muscle fiber 6/7 in control and *syd*^*z4*^*/Df* larvae. The *syd*^*z4*^*/Df* micrograph is shown with brightness and contrast settings matching those of the control (*middle*), as well as with the contrast digitally enhanced for better visibility (right). Scale bar: 10 µm. **(H)** Quantification of the presynaptic ILP2-GFP signal intensity in G. Number of larvae analyzed: control 8, *syd*^*z4*^*/Df* 8. Data in all panels are from third instar larvae. Bar graphs in B, C, E, F, G, and H represent mean + SEM and were analyzed using Student’s *t* test.

The LRRK2 kinase and its orthologs also bind to Syd family proteins (Choudhary et al., 2017; Hsu et al., 2010), and LRRK-phosphorylation of a subset of Rabs (including Rab8 and Rab10) on a conserved switch II residue promotes Rab binding to RH2 domains of RILP/RILPL and JIP3/4 (Bonet-Ponce et al., 2020; Waschbusch et al., 2020). However, when tested, animals lacking the sole fly LRRK ortholog did not show the DCV transport defects typically associated with Syd, RUFY, Rab2, or dynein dysfunction, although they did display a moderate decrease in the total axonal DCV cargo content (Fig. S6, A–C). The latter could be due to a partial kinesin-3–related cell body exit defect, as we also observed a mild accumulation of DCV cargo in ventral nerve cord (VNC) motor somata relative to peripheral nerves in *LRRK* null animals (Fig. S6, D and E). The apparent lack of retrograde transport defects in *LRRK* nulls is consistent with Rab10 being dispensable for DCV transport (Fig. S4, B and C) and the absence of any obvious effect on the Rab2^Q65L^:Syd-N2 Co-IP yield during pharmacological inhibition of LRRK2 activity or overexpression of mutant gain-of-function LRRK2 in HEK cells (Fig. S2 E).

### DCV membrane proteins are missorted to ectopic phase-separated vesicle aggregates in *Rab2* null cell bodies

Apart from the lysosome- and transport-related functions of Rab2, it is involved in DCV biogenesis in *C. elegans* (Ailion et al., 2014; Edwards et al., 2009; Sumakovic et al., 2009). Recent work in flies has further revealed that Rab2 plays a crucial role in generating specialized transport organelles that carry presynaptic components from somata to synapses (Götz et al., 2021). We therefore examined in more detail the distribution of lumenal and transmembrane DCV cargo in the form of ILP2-GFP and HA-VMAT, respectively, in *Drosophila* motor neurons in WT and *Rab2* null backgrounds.

**Figure 7.**
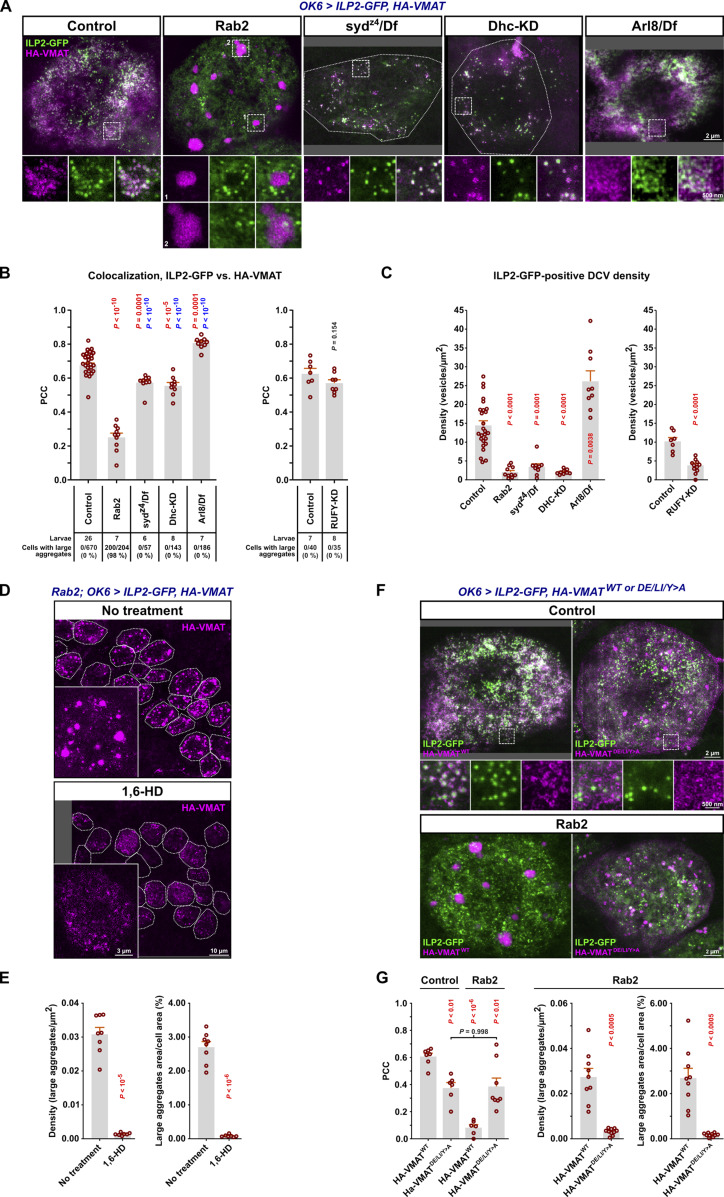
**Missorting of HA-VMAT to ectopic phase-separated vesicle aggregates and reduction of DCV numbers in cell bodies of *Rab2* mutants. (A)** Representative STED images showing the distribution of ILP2-GFP and HA-tagged WT VMAT in motor neuron cell bodies in VNCs of third instar larvae. KD, motor neuron-specific knockdown. Scale bar: 2 µm, 500 nm (insets). **(B)** Left, the co-localization of ILP2-GFP and HA-VMAT in A quantified using Pearson’s correlation coefficient (PCC). P values in red and blue result from comparison with the control and *Rab2* group, respectively. Right, the correlation between ILP2-GFP and HA-VMAT in motor neuron cell bodies of *RUFY-KD* and control larvae. Counts of VMAT aggregates made in confocal images are also shown (numbers below the graphs). **(C)** Left, ILP2-GFP-positive vesicle densities in cell bodies for the larval genotypes in A, and in *RUFY-KD* larvae with controls (right). Number of larvae analyzed in B and C: control 26, *Rab2* 10, *syd*^*z4*^*/Df* 9, *Dhc-KD* 9, *Arl8/Df*; control for *RUFY-KD* 7, *RUFY-KD* 8. **(D)** Representative confocal and STED (insets) images of WT HA-VMAT in motor neuron cell bodies from untreated *Rab2* third instar VNCs (top) or *Rab2* VNCs treated with 10% 1,6 hexanediol (1,6-HD) prior to fixation (bottom). Scale bar: 10 µm. **(E)** Density and relative area of large droplet-shaped VMAT-positive aggregates in motor neuron cell bodies in D. Number of larvae analyzed: control 8, 1,6-HD 7. **(F)** Representative STED images showing the distribution of ILP2-GFP and WT HA-VMAT or HA-VMAT^DE/LI/Y>A^ (HA-VMAT^D584A, E585A, L589A, I590A, Y600A^) in motor neuron cell bodies from control (top) and *Rab2* larvae (bottom). Scale bar: 2 µm, 500 nm (insets). **(G)** Left*,* quantification using PCC of the co-localization of ILP2-GFP with HA-VMAT or HA-VMAT^DE/LI/Y>A^ in control and *Rab2* larvae. Right, density and relative area of large droplet-shaped VMAT-positive aggregates in *Rab2* motor neuron cell bodies. Number of larvae analyzed in G, left: control*, HA-VMAT*^*WT*^ 7; control*, HA-VMAT*^*DE/LI/Y>A*^ 6; *Rab2, HA-VMAT*^*WT*^ 6; *Rab2, HA-VMAT*^*DE/LI/Y>A*^ 8. Number of larvae analyzed in G, *right*: *Rab2, HA-VMAT*^*WT*^ 9; *Rab2, HA-VMAT*^*DE/LI/Y>A*^ 10. Bar graphs in B, C, E, G represent mean + SEM. ANOVA followed by Tukey’s test (B, left; G, left), Steel with control test (C, left), Student's *t* test (B, right; C, right; E; G, right).

**Figure S7.**
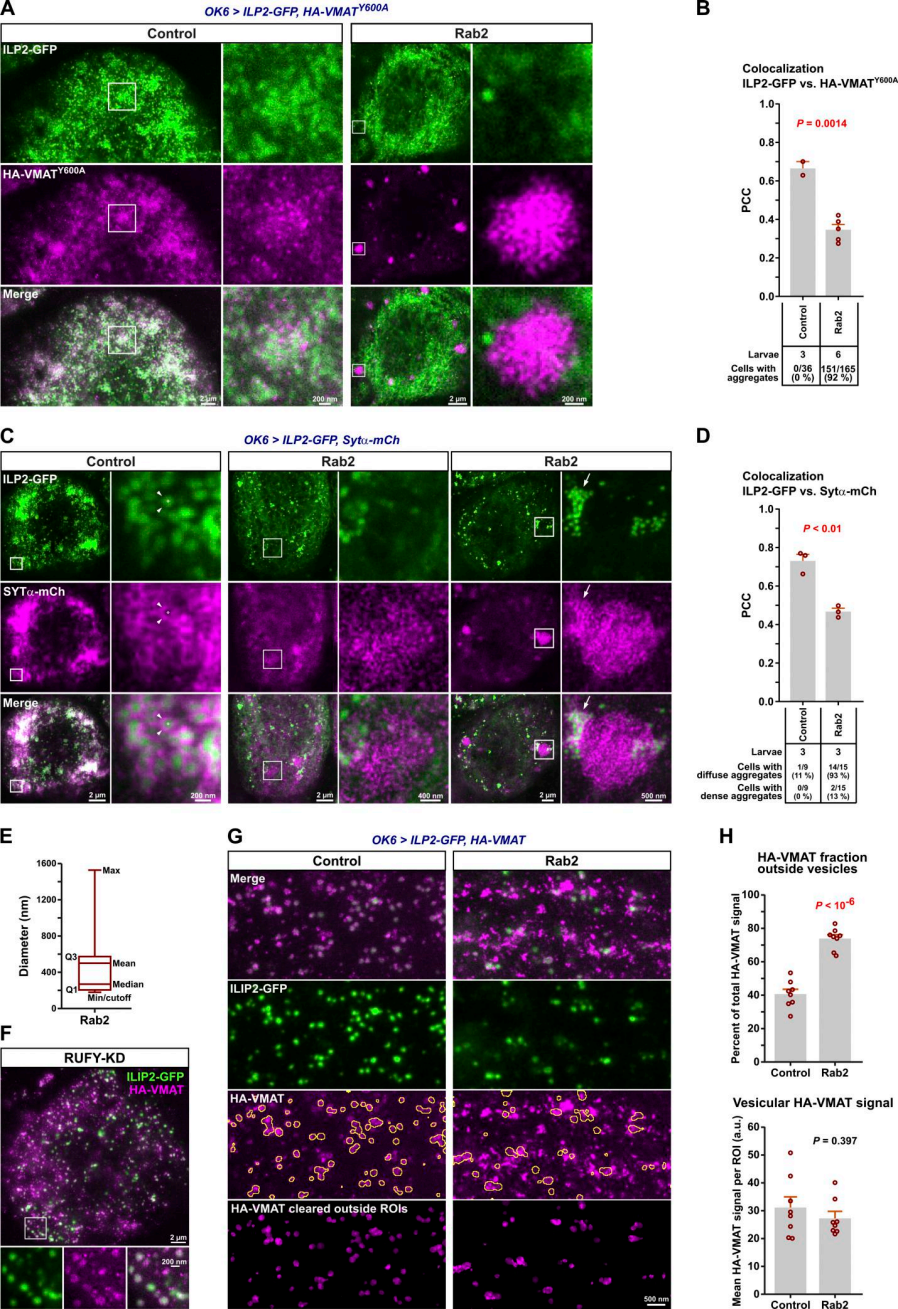
**Trafficking defects of HA-VMAT and SYTα-mCherry in motor neuron somata and axons of** ***Rab2*** **null larvae evaluated by STED microscopy.** **(A)** Spatial relationship between ILP2-GFP and HA-VMAT^Y600A^ in cell bodies. Representative STED images showing the distribution of ILP2-GFP and HA-tagged VMAT^Y600A^ in motor neuron cell bodies in VNCs of control and *Rab2* third instar larvae. Scale bars (left to right): 2 µm, 200 nm (inset), 2 µm, and 200 nm (inset). **(B)** Quantification of A. Pearson’s correlation coefficient (PCC) between the ILP2-GFP and HA-VMAT^Y600A^ signals is shown for two control and five *Rab2* larvae. Counts of HA-VMAT^Y600A^ aggregates are also given (numbers below graph). **(C)** Spatial relationship between ILP2-GFP and Sytα-mCherry in cell bodies. Representative STED images showing the distribution of ILP2-GFP and mCherry-tagged Sytα in motor neuron cell bodies in VNCs of control and *Rab2* third instar larvae. Examples of diffuse (middle) and dense (right) Sytα-mCherry aggregates in *Rab2* mutants are shown. Scale bars (left to right): 2 µm, 200 nm (inset), 2 µm, 400 nm (inset), 2 µm, and 500 nm (inset). **(D)** The PCC between ILP2-GFP and Sytα-mCherry is shown for three control and three *Rab2* larvae. Counts of diffuse and dense Sytα-mCherry aggregates are also given (numbers below graph). **(E)** Size distribution of VMAT aggregates (*n* = 262) in *Rab2* cell bodies (Fig. 7 A). Q1 and Q3, first and third quartile. Diameters were calculated from the areas of the aggregates, assuming a circular shape. A lower diameter cutoff of 178.4 nm (area 0.025 µ^2^) was applied. **(F)** Representative STED image showing the distribution of ILP2-GFP and HA-VMAT in a VNC motor neuron cell body from third instar larva subjected to motor neuron-targeted knockdown of *RUFY* (*OK6 > ILP2-GFP, HA-VMAT, RUFY-RNAi*^*VAL20*^*)*. Scale bars: 2 µm, 200 nm (inset). **(G)** Spatial relationship between ILP2-GFP and HA-tagged WT VMAT in motor axons. The multiple ROIs defined by yellow outlines mark the DCV-associated ILP2-GFP2 signal superimposed on the VMAT image. In the bottom row, the VMAT signal outside the DCV-associated ROIs has been digitally erased. Scale bar: 500 nm. **(H)** Quantification of E, showing the percentage of VMAT signal located outside the DCV-associated ROIs (top) and the VMAT signal intensity inside the ROIs (bottom) for eight control and eight *Rab2* larvae. Bar graphs in B, D, and H represent mean + SEM. Student’s *t* test (B, D, and H).

As described above (Fig. 1 D), VMAT mostly co-localized with abundant ILP2-positive DCVs when observed using STED microscopy in WT larval motor neuron cell bodies in the VNC (Fig. 7, A and B). Strikingly, in *Rab2* cell bodies, the number of DCVs was severely reduced, and most VMAT signal was no longer associated with ILP2 but instead segregated into large, dense, droplet-like aggregates averaging ∼0.5 µm in diameter (Fig. 7, A and B; and Fig. S7 E). These VMAT aggregates had a granular internal structure, characterized by particles with a diameter of ∼50 nm (see Materials and methods). This suggests that they are composed of smaller vesicles and are likely identical or similar to SV protein-containing tubulo-vesicular clusters previously described in *Rab2* larval neurons (Götz et al., 2021). These clusters, located in the vicinity of the TGN, were proposed to be the result of a failure to fuse small elongated (40 × 60 nm) Golgi-derived transport vesicles containing SV-proteins with morphologically similar vesicles containing presynaptic active zone scaffolds (Götz et al., 2021).

The dense, droplet-like appearance of the VMAT vesicle aggregates (Fig. 7, A and D) suggests that they form by liquid–liquid phase separation (LLPS), a chemical process whereby biopolymers and vesicles reversibly demix from the surrounding cytosol to form “membrane-less” organelles or biological condensates (Banani et al., 2017). Consistent with this notion, the VMAT aggregates were completely dispersed by treatment with 10% 1,6 hexanediol (Fig. 7, D and E), an aliphatic alcohol known to dissolve LLPS condensates (Guzikowski and Kavalali, 2024; Kroschwald et al., 2017).

The VMAT^Y600A^ mutant that is not efficiently targeted to SVs due to the disruption of a tyrosine-based (^600^YxxY^603^) AP-2 clathrin adaptor complex binding site but remains associated with DCVs (Grygoruk et al., 2014) still formed large, dense aggregates in *Rab2* nulls (Fig. S7, A and B). VMAT also contains a conserved acidic dileucine-like sorting signal (^584^DExxxLI^590^) that in mammals interacts with both AP-3 and retromer (Xu et al., 2022) and is responsible for DCV sorting of mammalian VMAT2 (Li et al., 2005; Waites et al., 2001). In contrast to the Y600A mutation alone, additional alanine substitution of the acidic dileucine-like motif (VMAT^DE/LI/Y>A^) both strongly reduced the association of VMAT with ILP2-positive DCVs in WT larvae and blocked the accumulation of VMAT in the large droplet-like aggregates in *Rab2* nulls (Fig. 7, F and G). Similar to WT VMAT, DCV-specific synaptotagmin-α tagged with mCherry (Park et al., 2014) redistributed away from somatal DCVs in *Rab2* mutants, although it was less prone to form dense droplet-like aggregates but instead mostly accumulated in more loosely organized vesicle clusters (Fig. S7, C and D).

These findings show that loss of Rab2 causes DCV-specific membrane proteins to accumulate in ectopic phase-separated vesicle condensates devoid of lumenal cargo. Formation of these vesicle condensates appears to occur downstream of an AP-3/retromer-dependent sorting event that directs VMAT into DCVs. We speculate that the condensates form because their constituent transport vesicles are unable to fuse to immature DCVs or the TGN in the absence of Rab2.

### Syd, RUFY, and dynein are not responsible for Rab2-dependent sorting of DCV membrane proteins but may play a role in biosynthetic transport of lumenal cargo

The results described above raised the question of whether Syd and RUFY and their associated motors contribute to Rab2-dependent functions during DCV biogenesis in the cell body. Alternatively, it is possible that the *Rab2* axonal transport phenotype is caused by a failure of Rab2-dependent sorting of critical DCV membrane proteins that serve as vesicular anchors for Syd and/or RUFY.

Neither *syd*^*z4*^*/Df* larvae nor larvae where RUFY or dynein were depleted by motor-neuron–specific RNAi displayed the strong separation of the VMAT and ILP2 signals or the large VMAT aggregates characteristic of *Rab2* nulls (Fig. 7, A and B; and Fig. S7 F). This shows that Syd, RUFY, and dynein do not mediate the Rab2-dependent sorting of SV/DCV membrane proteins and that this process must rely on one or more different Rab2 effectors.

Apart from the proximity and physical interactions between Rab2 and Syd (Fig. 1 B and Fig. 2), two lines of evidence suggest that the Rab2 axonal transport phenotype is not primarily due to the missorting of DCV membrane proteins. First, when we examined the axonal distribution of VMAT and ILP2, we found that axons in *Rab2* nulls exhibited a dramatic accumulation of VMAT-containing vesicles and smaller irregularly shaped vesicle clusters that were not associated with ILP2-positive DCVs (Fig. S7, G and H), partially mirroring the cell body phenotype. However, the mean VMAT signal associated with individual axonal DCVs was not significantly reduced in *Rab2* nulls compared with WT (Fig. S7 H). This suggests that remaining DCVs present in axons of Rab2-deficient animals are not strongly depleted of DCV-specific membrane proteins. Second, suppression of AP-3, which is required for sorting of DCV membrane proteins (including VMAT (Asensio et al., 2010)), by depletion of the critical AP-3 β3-adaptin/ruby subunit did not noticeably impact axonal DCV transport (Fig. S5, C and D). In light of the high enrichment scores for the Snx3–retromer and the Golgin245–TBC1D23–FAM91A1 tethering/fusion complex in Rab2- and VMAT-specific proximity proteomics (Fig. 1 G), we also considered the possibility that Rab2 functions in the retrieval of VMAT (and by extension other DCV membrane proteins) from endosomes to the TGN. However, depletion of the core retromer subunit Vps35 did not affect axonal transport of DCVs (Fig. S5, C and D), suggesting that defective endosomal retrieval is not the cause of the axonal transport defects in *Rab2* mutants.

In contrast to the Rab2-specific effect on VMAT sorting, larvae deficient in Syd, RUFY, or dynein all displayed dramatic reductions in the cell body density of ILP2-positive DCVs, as was also seen in *Rab2* mutants (Fig. 7, A and C). This effect can likely be partially explained by the trapping of DCVs in the axono-synaptic compartment by the loss of retrograde axonal transport, consistent with the observed increases in both proportion and absolute numbers of static axonal DCVs in these animals (Fig. 3, A and F–H). In comparison, loss of Arl8, which together with unc-104 is required for DCV cell body exit (Barkus et al., 2008; Lund et al., 2021), resulted in a large increase in the cell body DCV density (Fig. 7, A and C). However, axonal DCV trapping cannot fully explain the decrease in somatal DCV numbers in Rab2-, Syd-, RUFY-, and dynein-disrupted motor neurons, as these genotypes also display severe reductions in total axonal DCV cargo content (Fig. 4 A and Fig. S6 F). Furthermore, despite the relative DCV cargo enrichment in the most distal synaptic boutons (Fig. 4, B–D), overall synaptic DCV cargo content was also reduced in *Rab2* (Lund et al., 2021) and *syd* mutants (Fig. S6, G and H) compared with WT controls. Because our previous work indicates that DCV lumenal cargo loading is close to normal in *Rab2* nulls (Lund et al., 2021), this suggests that the total DCV number is substantially reduced in Rab2- and Syd-deficient neurons and likely also in RUFY- and dynein-depleted neurons. Since Rab2, dynein, and Syd are important for the function of the Golgi apparatus and/or ER-Golgi transport (Choudhary et al., 2017; Götz et al., 2021; Homma et al., 2019; Jaarsma and Hoogenraad, 2015; Tisdale et al., 2009), this may reflect a role for these proteins in trafficking of lumenal DCV cargo at early stages of the secretory pathway in a process that is not related to DCV membrane protein sorting.

Overall, we find that Rab2-dependent sorting of DCV membrane proteins is not mediated by Syd, RUFY, or dynein and is also unlikely to be responsible for the DCV axonal transport defects observed upon loss of Rab2. However, loss of Rab2, Syd, or dynein led to similar reductions in neuronal DCV numbers, possibly due to a dysfunction of lumenal DCV cargo trafficking at earlier stations of the secretory pathway.

## Discussion

We have investigated the machinery responsible for axonal circulation of DCVs in *Drosophila*, using its dependence on the small GTPase Rab2 as a starting point. We find that retrograde axonal DCV motility is mediated by the coiled-coil dynein and kinesin-1 adaptor Syd/dJIP3/4 and the coiled-coil dynein adaptor RUFY, which interact with the Rab2 and Arl8 GTPases. Given the severity of both the Syd loss-of-function phenotype and of the RUFY depletion phenotype, which is likely an underestimation of a full RUFY null phenotype, it seems likely that these proteins function as part of the same mechanism rather than in parallel to activate retrograde movement. Furthermore, the two adaptors bind to each other through the Syd C-terminal WD40 domain, and truncation of this region eliminates most or all Syd activity. These observations suggest a model where dynein–dynactin and kinesin-1 are activated and recruited by Syd anchored to the DCV membrane by a combination of Rab2 (potentially assisted by an unknown Rab2 effector) and RUFY, which itself is recruited by Arl8 and its activator BORC, and possibly Rab2 (Fig. 5 J). However, RUFY also has the long coiled-coil structure characteristic of dynein-activating adaptors, and there is evidence that mammalian RUFY1/3 interacts with dynein directly (Keren-Kaplan et al., 2022; Rawat et al., 2023). Fly RUFY has the RUN domain and central coiled-coil region 2 that are required for the interaction of RUFY1/3 with dynein (Keren-Kaplan et al., 2022; Rawat et al., 2023) and can also coprecipitate some fly DLIC (Fig. S2, F and G). We can therefore not exclude alternative scenarios where RUFY acts as the main activating adaptor for dynein, while Syd helps anchor RUFY to the vesicle membrane (and at the same time interacts with kinesin-1), or where the active transport complex contains two or more dynein dimers bound to a Syd:RUFY heterotetramer. The latter scenario would mirror recent findings showing that two dynein dimers form a complex with two BICDR1-activating adaptor dimers during transport (Chaaban and Carter, 2022). Further research is required to understand how Syd–RUFY-dependent motility functions and whether RUFY proteins can activate dynein directly.

The involvement of Arl8 in retrograde transport of DCVs via RUFY, in addition to its previously known role in anterograde transport via unc-104/kinesin-3, parallels recent results elucidating the role of mammalian ARL8 in regulation of lysosomal motility (Kendrick and Christensen, 2022; Keren-Kaplan et al., 2022; Kumar et al., 2022; Rawat et al., 2023). ARL8 also recruits kinesin-1 through the SKIP/PLEKHM2 adaptor (Farias et al., 2017; Keren-Kaplan and Bonifacino, 2021), but the fly SKIP ortholog, prd1, lacks the Arl8-binding RUN domain, and prd1 knockout larvae did not exhibit any obvious DCV transport defects (Fig. S3, E and F). Our observed *Arl8* null phenotype (Lund et al., 2021) has features in common with both *unc-104* and *syd/dynein/kinesin-1* mutants, in that it both exhibited a cell body exit defect and a strong increase in the proportion of static vesicles among those remaining in the axons (Fig. 3). In comparison, the few remaining axonal DCVs in *unc-104*^*P350*^*/unc-104*^*O3.1*^ hypomorphs were mostly motile but much slower when moving in the anterograde direction (Fig. 3), as reported previously (Lim et al., 2017). Meanwhile, depletion of kinesin-1 or dynein, apart from selective reductions in retrograde transport flux and movement speed (Fig. S3, A and B), produced large increases in the proportion of static axonal DCVs (Fig. 3). As also noted by others (Gavrilova et al., 2024), this suggests that the main role of kinesin-3 is to move DCVs out of the soma and to enhance the anterograde axonal DCV velocity, while kinesin-1 and dynein are critical for maintaining bidirectional motility among the axonal DCVs, and their absence leads to axonal vesicle stalling. This difference likely relates in large part to distinct interactions of kinesin-1 and -3 with differentially distributed MT-binding proteins (Gumy et al., 2017; Monroy et al., 2018). Moreover, the kinesin-1- and dynein-mediated axonal DCV motility appears to be partially maintained by JNK signalling (Fig. 6). Surprisingly, the knockout phenotype for the BORC subunit Blos1 (Fig. 5, E and F) was closer to that exhibited by Syd/RUFY/kinesin-1/dynein than was the Arl8 knockout phenotype. This difference may reflect graded phenotypic effects of different levels of remaining Arl8 activity or that BORC only activates Arl8 in the axonal compartment, while a different Arl8 activator facilitates DCV sorting from the soma to the axon. However, it is possible that BORC could directly interact with RUFY or Syd. Generally, axonal DCV transport appears to use much of the same adaptor-motor components as lysosomes (including Syd/dJIP3/4, RUFY, Arl8, and Rab2 [Cason and Holzbaur, 2023; Drerup and Nechiporuk, 2013; Farias et al., 2017; Gowrishankar et al., 2021; Hofmann and Munro, 2006; Keren-Kaplan et al., 2022; Kluss et al., 2022; Kumar et al., 2022; Vukoja et al., 2018]), possibly reflecting an evolutionary and/or biosynthetic connection between these organelles.

Interestingly, in our PB experiments we observe strong enrichment for lysosomal/autophagic and Golgi-related proteins in the Rab2^Q65L^-specific dataset, but less so for ER proteins (Fig. 1 C), as might have been expected from early studies that indicated that Rab2 is responsible for retrograde transport from Golgi-ER intermediates to the ER (Tisdale, 1999; Tisdale and Jackson, 1998). These findings are, however, consistent with newer work that places Rab2 as a critical factor in lysosome function and biogenesis (Lorincz et al., 2017; Lund et al., 2018), autophagy initiation and clearance (Ding et al., 2019; Fujita et al., 2017), and tethering at the Golgi apparatus (Gillingham et al., 2014; Short et al., 2001; Sinka et al., 2008). Apart from most previously known Rab2 effectors, the Rab2^Q65L^-PB data set also contained several proteins detected by *Drosophila* Rab2 affinity-purification MS or human Rab2A MitoID but not validated as effectors (Gillingham et al., 2014, 2019), including RUFY and gartenzwerg/GBF1. GBF1 is a Golgi-localized Arf1 activator promoting COPI vesicle budding (Garcia-Mata et al., 2003) and could thus provide a link between Rab2 and retrograde Golgi-to-ER trafficking. Curiously, both the Rab2^Q65L^- and VMAT-PB datasets showed high enrichment for the transmembrane autophagy factor Atg9 (Fig. 1 G), which may reflect the newly uncovered ties between the synaptic exo-endocytic cycle and synaptic autophagy (Karpova et al., 2025) and/or that Atg9 follows the same Rab2-dependent sorting pathway in the soma as VMAT (see below).

In line with *C. elegans* work demonstrating a role for Rab2 and its effectors in DCV biogenesis (Ailion et al., 2014; Edwards et al., 2009; Sumakovic et al., 2009; Topalidou et al., 2016), we observed that Rab2 loss causes a severe DCV membrane protein sorting defect in flies. In *Rab2* null fly neurons, VMAT accumulated in the soma in phase-separated vesicle aggregates closely resembling the TGN-proximate SV-component–containing vesicle clusters previously observed in *Rab2* mutants (Götz et al., 2021). This suggests that DCV and SV membrane protein cargo is sorted into small transport vesicles during DCV/SV-transport organelle biogenesis, which fail to fuse to their target compartment and accumulate in the absence of Rab2. The phase separation from the surrounding cytosol likely results from a high abundance of LLPS-prone synaptic proteins in the transport vesicles and might therefore represent an ectopic form of the normal LLPS-driven SV clustering at the presynapse (Guzikowski and Kavalali, 2024; Milovanovic and De Camilli, 2017). Moreover, VMAT sorting into these transport vesicles depends on a conserved acidic dileucine-like signal, which is also required for DCV targeting. The vesicle target compartment may be immature DCVs, SV precursors, or the TGN. In fact, substantial enrichment for both retromer and Rab2-binding TGN tethering factors (Golgin245 and Golgin-104) in the Rab2^Q65L^- and VMAT-PB datasets suggests that these transport vesicles may originate in endosomes and carry their cargo to the TGN. This correlates with recent work showing that DCV membrane cargo (including VMAT) initially exits the Golgi apparatus separately from lumenal cargo (Hummer et al., 2020) and that endosomal recycling plays a critical role in DCV biogenesis (Laurent et al., 2018; Xu et al., 2022).

In summary, we find that coupling of kinesin-1 and dynein to DCVs for axonal transport happens via Syd/dJIP3/4 and RUFY that are controlled by Rab2 and Arl8/BORC, but that these adaptors are not required for Rab2-dependent DCV membrane cargo sorting.

## Materials and methods

### Fly husbandry and genetics

Fly stocks were maintained on Nutri-Fly Bloomington Formulation medium (Genesee Scientific) at 25°C. For experiments involving live imaging of axonal transport or immunostaining of larval tissues, all larvae were reared on apple juice (AJ) plates (27 g/L agar, 12 g/L sucrose, and 1.875 g/L nipagin [methyl 4-hydroxybenzoate]) supplemented with yeast paste. After an overnight lay, plates were incubated for ∼48 h at 25°C to allow all eggs to hatch. L1–2 larvae of the desired genotype (determined by presence or absence of appropriate fluorescence) were then transferred to fresh yeasted AJ plates for another ∼48 h (∼96 h after end of egg laying) before being dissected. For RNAi experiments, flies were reared at 29°C and picked for dissection ∼72 h after the end of egg laying to account for the faster pace of development at higher temperature. To increase knockdown efficiency, UAS-Dicer-2 was co-overexpressed with the RNAi transgenes, except when short hairpin RNAs were used. In matching RNAi controls, UAS-Dicer-2 was overexpressed alone under control of the appropriate driver. Fly lines and the source from which they were obtained are listed in Table S1. Genotypes used in figures and Video 1 are listed in Table S4.

### *Drosophila* Phi31C transformation

Fly embryo DNA injections and selection of Phi31C transformants were performed by BestGene Inc. The UAS-HA-TurboID-VMAT, UAS-HA-TurboID, UAS-HA-VMAT, and UAS-HA-VMAT^D584A, E585A, L589A, I590A, Y600A^ transgenes were inserted into the M{3xP3-RFP.attP}ZH-86Fb attP site on the third chromosome using Phi31C transformation to ensure comparable levels of expression.

### Molecular biology

All constructs (listed in Table S2) were generated by GenScript Biotech (Netherlands) BV, except pCMV5-FLAG-LRRK2[G2019S], which was obtained from MRC PPU Reagents and Services (University of Dundee, Scotland). All constructs for protein expression in mammalian cell culture generated for this study were made in the pCDNA3.1(+) vector and were based on the following protein isoforms: Syd isoform A (UniProt Q9GQF1), RUFY/CG31064 isoform G (UniProt A0A0B4LHR8), dNischarin/CG11807 (UniProt Q7K490), DLIC isoform A (UniProt Q9VZ20), Klc isoform A (UniProt P46824), Rab2 (UniProt O18333), and Arl8 (UniProt Q9VHV5). Constructs for *Drosophila* Phi31C transformation were generated in the pUASTattB vector.

### In vivo proximity biotinylation and purification of biotinylated proteins

Flies expressing TurboID-Rab2^S20N^, TurboID-Rab2^Q65L^, TurboID-VMAT together with ILP2-GFP, or free TurboID together with ILP2-GFP under control of the pan-neuronal elav-Gal4 driver were reared at 25°C on Nutri-Fly Bloomington Formulation medium supplemented with 100 μM biotin. (ILP2-GFP was co-overexpressed with both TurboID-VMAT and free TurboID transgenes to stimulate DCV production). Adult flies were collected 0–3 days after eclosion, flash-frozen in liquid nitrogen, and stored at −80°C. For purification of biotinylated proteins, 0.5–1 ml of frozen flies was transferred to pre-cooled dounce homogenizers on ice, quickly dounced five times, then dounced 15 times in 3 mL RIPA lysis buffer (50 mM Tris, pH 7.5, 150 mM NaCl, 0.5% sodium deoxycholate, 1.0% NP-40, 0.1% SDS, 1 mM DTT, cOmplete Protease Inhibitor Cocktail [1 tablet/25 ml] [Roche, Ref: 11836145001], and 1 mM PMSF). Lysates were then incubated for 30 min on ice and again dounced 15 times. Hereafter, the lysates were centrifuged 3 times for 15 min at 50,000 *g* and passed through 40-µm Cell Strainers (Cat. no. 22363547; Fisherbrand) to remove insoluble debris. Protein concentrations in the resulting lysate supernatants were measured using the BCA assay (Pierce BCA Protein Assay Kit; Ref: 23225; Thermo Fisher Scientific) and adjusted to 2.0 mg/ml. 3.4 ml of each lysate was precleared for 1 h at 4°C under rotation with 300 μl Sepharose 4B beads (4B200-100ML, Lot# MKCJ6278; Sigma-Aldrich), previously equilibrated in RIPA buffer. Sepharose 4B beads were then removed by gentle centrifugation, and 1.5 ml of each supernatant was incubated overnight at 4°C under rotation with 50 μl Dynabeads MyOne StreptavidinT1 (Ref 65601, Lot: 00804134; Thermo Fisher Scientific). Dynabeads were then magnetically concentrated and washed one time with 1 ml high-SDS RIPA buffer (50 mM Tris, pH 7.5, 150 mM NaCl, 0.5% sodium deoxycholate, and 1.0% NP-40, 0.4% SDS) at RT, then transferred to clean sample tubes in a new wash of high-SDS RIPA. They were then washed two times with SDS wash buffer (50 mM Tris, pH 7.5; 2.0% SDS), one time with high-SDS RIPA buffer, one time with normal RIPA buffer, and finally two times with PBS and again transferred to clean tubes in a third wash of PBS.

### LC-MS analysis of biotinylated proteins

Washed beads were eluted by a 30-min incubation at 37°C in elution buffer 1 (2 M urea, 50 mM Tris-HCl, pH 7.5, 2 mM DTT, and 20 μg/ml trypsin) followed by a second elution step for 5 min in elution buffer 2 (2 M urea, 50 mM Tris-HCl, pH 7.5, and 10 mM chloroacetamide). Both eluates were combined and further incubated at RT overnight. Tryptic peptide mixtures were acidified to 1% TFA and loaded onto Evotips (Evosep). Peptides were separated on a Pepsep 15-cm, 150-μM ID column packed with C18 beads (1.5 μm) using an Evosep ONE HPLC system applying the default 30 samples per day method. The column temperature was maintained at 50°C. Peptides were injected via a CaptiveSpray source and 20 μm emitter into a timsTOF pro2 mass spectrometer (Bruker) operated in PASEF mode. MS data were collected over a range of 100–1,700 m/z with a TIMS mobility range of 0.6–1.6 1/K0. TIMS ramp and accumulation times were set to 100 milliseconds, with 10 PASEF ramps recorded for a total cycle time of 1.17 s. The MS/MS target intensity and intensity threshold were set to 20,000 and 2,500, respectively. An exclusion list of 0.4 min was activated for precursors within 0.015 m/z and 0.015 V cm^−2^ width.

### MS data analysis

Raw MS data were analyzed using MaxQuant (version 1.6.15.0). Peak lists were searched against the human UniProt FASTA database, combined with 262 common contaminants, using the integrated Andromeda search engine. A false discovery rate (FDR) of 1% was set for both peptides (minimum length of 7 amino acids) and proteins. Carbamidomethylation of cysteine was specified as a fixed modification, while oxidation of methionine; acetylation at the protein N terminus; acetylation of lysine; and phosphorylation of serine, threonine, and tyrosine were considered variable modifications. Additionally, “Match between runs” was enabled with a match time window of 0.7 min and a match ion mobility window of 0.05 min.

All statistical analysis was conducted using in-house developed Python code (Santos et al., 2022). LFQ intensity values were log2-transformed, and features with <70% of valid values in at least one group were eliminated. Remaining missing values were replaced by mixed imputation, where the kNN and MinProb (width = 0.3 and shift = 1.8) methods are used for values missing at random (MAR) and values missing not at random, respectively (Lazar et al., 2016). MAR is defined when a minimum of 60% of the samples within a given group have an existing value. Differentially expressed features were identified by unpaired Student’s *t* tests, followed by Benjamini–Hochberg correction for multiple hypothesis testing with a FDR threshold of 0.05 and a fold change of 2.

### HEK cell transfection and Co-IP

HEK293 (#CRL-1573; ATCC) cells were maintained in DMEM w. HEPES and NaHCO3 (University of Copenhagen, Substrat og SterilCentralen, Ref #: 12) supplemented with 10% Standard Fetal Bovine Serum (Ref: 10270-106; Gibco), 200 U/ml penicillin, and 50 μg/mg streptomycin.

For all Co-IP experiments, T75 cell culture flasks with ∼7*10^6^ cells per flask (corresponding to a cell density of ∼10^5^ cells/cm^2^) were transfected with a total of 1–7 µg of DNA using 3 μl of Lipofectamine 2000 (Ref: 11668-019; Invitrogen) per 1µg of DNA. Cells were grown for ∼48 h after transfection to allow for recombinant protein expression. In the experiment to test for dependence of Rab2:Syd complex formation on LRRK phosphorylation, cells were also exposed to 2 µM MLi-2 in the medium for 2 h immediately before being harvested.

The Co-IP protocol was adapted with modifications from the one used by Vukoja et al. (2018) to show the interaction between Arl8 and Unc-104 (Vukoja et al., 2018). At the end of the expression period, cells were washed twice in ice-cold PBS and harvested in 1.5 ml ice-cold Co-IP lysis buffer (20 mM HEPES, pH 7.4, 130 mM NaCl, 2 mM MgCl2, 0.1% wt/vol saponin, and cOmplete Protease Inhibitor Cocktail [1.5 tablet/50 ml] [Ref: 11836145001; Roche]) using a cell scraper. The Co-IP lysis buffer used to handle samples with active GTPases (Rab2(wt) and Rab2[Q65L], but not Rab2[S20N]) was supplemented with 60 μM GppNHp to lock the GTPases in their active conformation. In some experiments, the lysis buffer was also supplemented with 1:285 Phosophatase Inhibitor Cocktail 2 (P5726; Sigma-Aldrich), but this practice was discontinued after it was established that LRRK activity is not required for Rab2 binding to Syd. Cell suspensions were lysed by being forced through a 25-G needle six times. Resulting lysates were incubated on ice for 30 min, and insoluble debris was removed by a pair of consecutive 12k *g* centrifugation steps of 10 and 5 min, respectively. Supernatant protein concentrations were measured using the BCA assay (Pierce BCA Protein Assay Kit, Ref: 23225; Thermo Fisher Scientific) and adjusted to 1.0 mg/ml. 1.2 ml of each lysate supernatant was incubated with 22 μl Pierce Anti-HA Magnetic Beads (Ref: 88836; Thermo Fisher Scientific) or Pierce Anti–c-Myc Magnetic Beads (Ref: 88842; Thermo Fisher Scientific) for 2 h at 4°C under rotation. Magnetic beads were then washed four times with standard Co-IP lysis buffer with 0.1% saponin (anti-HA beads) or two times with Co-IP lysis buffer with 0.25% saponin followed by two times Co-IP lysis buffer with 0.1% saponin (anti–c-Myc beads). During each wash, beads were resuspended by pipetting and then separated from the supernatant using a DynaMag-2 magnetic rack (Ref: 12321D; Thermo Fisher Scientific). Beads were also transferred to new Eppendorf tubes in the second wash. Bound proteins were eluted from anti-HA beads by either incubation in 40 μl 100 mM NaOH for 10 min at RT or with 40 μl 2.5 mg/ml HA peptide (Cat #: 26184; Thermo Fisher Scientific) for 20 min at 37°C, and from anti–c-Myc beads by heating in SDS-PAGE loading buffer diluted 1:2 in wash buffer. Eluates and lysate aliquots were mixed with standard Laemmli SDS-PAGE loading buffer and heated to 99.9°C for 10 min in preparation for SDS-PAGE. All Co-IP experiments except the experiment in Fig. S2 A were performed at least twice.

### Western blotting

For SDS-PAGE, protein samples were run on AnyKD Mini-PROTEAN TGX Precast Protein Gels (Ref: 4569033; BioRad) clamped to 100V. Separated proteins were transferred to PVDF membranes using the Trans-Blot Turbo Transfer System (Ref: 1704150; BioRad) running a custom transfer program (1.3A, 25V, 20 min, current clamped). Membranes were blocked overnight at 4°C, then probed with primary antibody in blocking buffer (PBS, pH 7.4, 0.05% Tween-20 vol/vol, and 5% vol/wt Skim Milk Powder [Ref: 70166; Sigma-Aldrich]) for 1 h at RT. After three 10-min washes in wash buffer (PBS, pH 7.4; 0.05% Tween-20 vol/vol), membranes were incubated with HRP-conjugated secondary antibody in blocking buffer for 1 h at RT. Membranes were then again washed for three times 10 min in wash buffer and one time 5 min in PBS before being deposited in deionized water. Chemiluminescent signals were developed by incubating the membranes for 10 min in SuperSignal ELISA Femto Maximum Sensitivity Substrate (Ref: 37075; Thermo Fisher Scientific Scientific). Membrane imaging was performed on an Amersham ImageQuant 800 luminescence imager using the Signal-to-Noise Optimization Watch capture mode. Antibodies used for western blotting are listed in Table S3.

For Co-IP experiments, between 40% and 15% of the total eluate volume and a lysate volume corresponding to ∼1% of the binding reaction were loaded on each gel. In experiments shown in Fig. 2, B, D, E–G, I, and K; and Fig. S2, B, D, and E, membranes with lysate and eluate samples were developed separately as the Co-IP yields were relatively low. In Fig. 2 A; Fig. 5, A and B; and Fig. S2 F, lysate and eluate samples were developed together on the same membrane.

### Immunohistochemistry

For staining of neuronal somata in the larval VNC and of larval peripheral nerves, third instar larvae were dissected in PBS) containing 137 mM NaCl, 2.7 mM KCl, 1.5 mM KH2PO4, and 6.5 mM NaH2PO4, pH 7.4. Larval CNS (still attached to a piece of anterior cuticle for easier handling) were extracted and briefly stored in Schneider’s insect cell medium (A820; Life Technologies) supplemented with 5% heat-inactivated FBS at RT prior to fixation. For staining of larval NMJ synapses, third instar larvae were pinned down using 0.1-mm Minutien Pins (Fine Science Tools) on ∼1.3-cm Ø slabs made of SYLGARD (Dow). They were then fillet dissected in modified HL3 solution (70 mM NaCl, 5 mM KCl, 10 mM NaHCO3, 20 mM MgCl2, 5 mM trehalose, 115 mM sucrose, 0.5 mM EGTA, and 5 mM HEPES, pH 7.2). Isolated CNSs and larval fillets were fixed in 3.7% formaldehyde in PBS at RT for 50 min. Specimens were then washed six times 10 min in PBS with 0.3% Triton X-100 (PBX [Ref: T8787; Sigma-Aldrich]), blocked for 2 h at RT in blocking buffer (PBX with 10% goat serum [Ref: G9023; Sigma-Aldrich]), and incubated for 72 h at 4°C in primary antibodies in antibody incubation buffer (PBX with 5% goat serum). This was followed by six 10-min washes in PBX at RT and an overnight incubation at 4°C with secondary antibodies in antibody incubation buffer. Finally, specimens were subjected to another set of six 10-min PBX washes, followed by two 5-min washes in PBS, and mounted in ProLong Gold antifade reagent (P36934; Life Technologies). All incubations were done under gentle agitation.

For standard confocal microscopy, secondary or primary antibodies were labelled with Alexa 488, Alexa 647, or Rhodamine Red-X dyes. For STED microscopy, antibodies were labelled with Abberior STAR RED and Abberior STAR ORANGE. Antibodies used for immunohistochemistry, along with their working concentrations, are listed in Table S3.

### 1,6-hexanediol treatment

CNS and attached mouthparts were dissected out of third instar larvae and briefly stored in Schneider’s insect cell medium with 5% heat-inactivated FBS, then in PBS for ∼30 min. They were then incubated in either fresh PBS as the control condition or PBS with 10% 1,6 hexanediol for 9 min at RT. The specimens were then fixed in 3.7% formaldehyde in PBS at RT for 55 min (control condition) or for 15 min in a mixture of 3.7% formaldehyde and 5% 1,6 hexanediol in PBS, followed by 3.7% formaldehyde in PBS for the remaining 40 min (1,6 hexanediol condition). After fixation, the specimens were processed for immunohistochemistry as described above.

### Conjugation of fluorophore to nanobody

In preparation for STED imaging, alpaca anti-GFP V_H_H single-domain antibody/nanobody (gt-250, Lot: 71017001U; Chromotech) was conjugated to Abberior STAR ORANGE NHS ester (Ref: STORANGE-0002-1MG, Lot: 10319RK-1; Abberior), then isolated through Zeba Spin Desalting Columns, 7K MWCO (Ref: 89883; Thermo Fisher Scientific). This was completed by first washing the column three times with 300 μl of 100 mM NaHCO_3_ in PBS. Following the washes, 100 μg of nanobody in 200 μl of PBS was added and spun at 1,500 *g* for 2 min. The flow-through was collected and combined with a fivefold molar excess of NHS ester fluorophore. This solution was incubated in the dark at RT, shaking, for 2.5 h. A new spin column was washed three times with 0.02% NaN_3_ in PBS. The antibody sample with the dye was then added to the column and centrifuged for 2 min at 1,500 *g*. Protein and label concentrations were measured on an Eppendorf BioPhotometer Plus spectrophotometer. The resulting STAR ORANGE-conjugated nanobody had a labelling rate of >0.4 fluorophores/molecule.

### STED microscopy

STED microscopy was performed at the Core Facility for Integrated Microscopy (CFIM, Department of Biomedical Sciences, University of Copenhagen, København, Denmark) using an Abberior STEDYCON system mounted on a Zeiss AxioImager Z1 wide-field microscope with an alpha Plan-Apochromat 100×/1.46 Oil DIC VIS M27 objective. The Abberior STEDYCON smart control browser-based software was used for image acquisition, with pixel sizes being 20 or 15 nm.

### Confocal microscopy

Confocal microscopy was carried out at CFIM using an LSM 700 confocal microscope (Carl Zeiss Microscopy GmbH, Jena, Germany) or an Abberior STEDYCON system mounted on a Zeiss AxioImager Z1 microscope and the following objectives: Plan-Apochromat 63×/1.4 Oil DIC (for IHC samples), alpha Plan-APOCHROMAT 100×/1.46 Oil DIC VIS M27, W N-Achroplan 10×/0.3 M27 #420947-9900 water dipping (for live imaging), or W Apochromat 40×/1.0 DIC VIS-IR #421462-9900 water dipping (for live imaging). Live confocal microscopy was performed as follows (Lund et al., 2021). Fillet-dissected third instar larvae pinned down in SYLGARD dishes were imaged directly in modified HL3 using the LSM700 microscope equipped with a water dipping objective. For assessment of axonal transport of DCVs and lysosomes, A7 peripheral nerves were imaged in a 128 µm long segment 0.5–1.0 mm from the nerve egress from the VNC using the W Apochromat 40×/1.0 DIC VIS-IR #421462-9900 objective. After recording a pre-bleach image, the 60 µm flanking sections were photobleached using a 405 nm laser, and subsequent time-lapse imaging was performed for 499 frames (corresponding to ∼106 s for DCVs and ∼212 s for lysosomes due to higher averaging). In a few cases, imaging was performed in younger larvae, as specified in the text and figures. For assessment of DCV cargo distribution between motor neuron cell bodies in the VNC and proximal peripheral nerve axons (Fig. S6, D and E), confocal z-stacks were recorded from living third instar fillet preparations using a W N-Achroplan 10×/0.3 M27 #420947-9900 water dipping objective.

During confocal imaging, the LSM700 microscope was controlled using the ZEN 2012 SP5 FP3 (black) 14.0.29.201 software, and the STEDYCON system was controlled using the STEDYCON smart control browser-based software. Confocal and STED imaging were performed at RT (∼20°C).

### Image analysis

Confocal and STED images were analyzed using the Fiji/ImageJ package (Schindelin et al., 2012). Pre-bleach nerve fluorescence was quantified as the total integrated density of the A7 mid-nerve segment subsequently undergoing time-lapse imaging of vesicle transport.

### Axonal vesicle transport

A custom algorithm was used to generate kymographs from time-lapse recordings. For presentation in figures, kymographs were digitally inverted. To produce directional distributions, kymographs were rotated 90° counterclockwise and subjected to the Directionality plugin in Fiji, selecting Fourier spectrum analysis. For statistical analysis, the peak relative frequency of directional distribution angles was determined within the following intervals: between −87.98° and−45.51° (retrograde peak, *P*_*ret*_), between 45.51° and 87.98° (anterograde peak, *P*_*ant*_), and between −13.14° and 13.14° (static peak, *P*_*stat*_). The static relative peak amplitude, *P*_*stat(rel)*_, was calculated using the expression:

$$P_{stat\left(rel\right)}=P_{stat}\;/\;\left(P_{ret}+P_{stat}+P_{ant}\right).$$ (1)

Directional distribution angles were converted to transport velocities using the expression:

$$V=tan\left(A\right)\;*\;{CF}_{s}\;/\;{CF}_{t}\text{.}$$ (2)

where *V* is transport velocity in µm/s*, A* is the angle, and *CF*_*s*_ and *CF*_*t*_ are conversion factors for the space and time axes of the kymograph, respectively. For DCV experiments, *CF*_*s*_ was 0.1 µm/pixel and *CF*_*t*_ was 0.2125 s/pixel. For all other genotypes than *Arl8/Df*, a directional distribution was generated from each kymograph. The paucity of DCVs in *Arl8* mutant nerves made this approach less attractive. Instead, one single directional distribution was produced from a maximum intensity projection of 20 stacked kymographs (reanalysis of data from nine *Arl8/Df* larvae, published previously [Lund et al., 2021]).

Anterograde and retrograde DCV flux in axons during the initial 30 s of the recording session was quantified by counting unbleached DCVs that entered the bleached areas from the left and right sides, respectively, and travelled at least 1.8 µm further along the axon. To facilitate DCV tracking, images were Gaussian blurred, and DCV centers were marked with a black dot using the Find Maxima plugin in Fiji to locate fluorescence peak intensities. DCV flux in *syd* and *Rab2, syd* double mutants were quantified using kymographs. Static (i.e., not moving in 30 s) vesicles were counted by inspecting “kymostacks” of the central unbleached region (Lund et al., 2021); kymostacks were generated by producing a separate kymograph for each of the 56 lines (y coordinates, represented as pixels) in the time-lapse recordings.

To quantify DCV speed, a segmented line was manually fitted to the trajectory of individual vesicles in the kymographs, with each line segment representing an anterograde run, a retrograde run, or a pause if DCV speed was <0.015 μm/s (Lund et al., 2021). Pauses were excluded before calculating the average run speed per vesicle. For each trajectory, anterograde and retrograde runs were analyzed separately. Due to the high density of DCVs in axons from the *OK6 > ILP2-GFP* control larvae, speed measurements in control axons were facilitated by producing “partial kymographs” derived from regions of interest (ROIs) having about 5–10% of the axon width, rather than from standard full-width ROIs that were used for other genotypes with smaller DCV density. Moreover, to ensure unbiased speed estimates, an average of 60 (range 44–94) representative DCV trajectories, equally dispersed across the kymographs, were sampled from each control axon.

As for DCVs, directional distributions were produced to quantify axonal transport of lysosomal organelles labelled with Spinster-Venus in motor axons of *syd* larvae and matching controls. To relate these results to Rab2’s role in lysosomal transport, directional distributions were also generated of Spinster-GFP–positive lysosome transport in *Rab2* larvae and their controls (reanalysis of data published earlier [Lund et al., 2021]). Eq. 2 was used to convert the directional angles to lysosome transport velocities, with *CF*_*s*_ equal to 0.1 µm/pixel and *CF*_*t*_ equal to 0.4251 s/pixel.

### ILP2-GFP intensity in nerve terminals

The overall ILP2-GFP immunosignal was measured as the integrated density on background-subtracted sum projections of z-stacks traversing the entire nerve terminal. To quantify the intensity gradient of the ILP2-GFP signal in distal boutons of individual end branches, images were Gaussian blurred before drawing segmented lines through the boutons in each branch, starting with the end bouton. The corresponding intensity profiles (where the x axis represents distance along the line and the y axis pixel intensity) were obtained using the Fiji plot profile plugin. The amplitudes of the first five peaks in the profile were taken as the intensity of the distalmost five boutons. Intensities were standardized by dividing by their mean. Slopes of linear regression lines in plots of the five peak amplitudes against their x positions were calculated using Excel software.

### ILP2-GFP intensity in VNC somata and A7 nerves

Sum projections of confocal z-stack micrographs of third instar VNCs, including the proximal aspect of the peripheral nerves, were background-subtracted and thresholded to quantify the VNC ILP2-GFP signal. To quantify the ILP2-GFP signal in nerves, images were Gaussian blurred before obtaining the intensity profile across the A7 nerves 250 µm caudal to the VNC. The average of the peak ILP2-GFP intensities of the left and right A7 nerves was used.

### HA-VMAT aggregate frequency, density and relative area

To determine the abundance of large HA-VMAT aggregates, motor neuron somata found to contain rounded, sharply demarcated “drop-like” aggregates of HA-VMAT–positive vesicles were counted using confocal micrographs of the dorsal surface of the larval VNC.

To determine the density and relative area of large drop-like HA-VMAT aggregates, confocal images of the HA-VMAT signal in the dorso-medial aspect of larval VNCs were subjected to Gaussian blur (σ = 30 nm) and thresholded to the 2% most intense pixels before using Fiji’s particle analysis plugin to obtain the number and area of HA-positive aggregates larger than 0.45 µm^2^ and with a circularity above 0.80. These numbers were then divided by the number and area of HA-VMAT–expressing cell bodies determined from the same image.

### STED micrograph analysis

To quantify the extravesicular percentage of the HA-VMAT immunosignal in axons, images were background-subtracted and the ILP2-GFP channel thresholded to include the 10% most intense pixels. This threshold was converted to a ROI set that was restored on the HA-VMAT channel. The HA-VMAT intensity (integrated density) of the ILP2-GFP–associated ROI set was divided by the total HA-VMAT intensity obtained after also thresholding the HA-VMAT channel to 10%. The resulting intravesicular HA-VMAT signal percentage was subtracted from 100 to obtain the extravesicular percentage. The mean vesicular HA-VMAT intensity was measured in individual ROIs from the ILP2-GFP–associated ROI set.

To estimate the density of ILP2-GFP-containing vesicles in cell bodies, the intensity of the total ILP2-GFP immunosignal was divided by the intensity of individual vesicles, calculated as an average of generally 5–10 isolated vesicles. The resulting vesicle count was finally divided by the cell area. Calculation of Pearson’s correlation coefficient to quantify ILP2-GFP vs. HA-VMAT and ILP2-GFP vs. Sytα-mCherry colocalization was restricted to cell body ROIs.

The size of ILP2-GFP–positive DCVs and small HA-TurboID-VMAT–positive vesicles in presynaptic boutons was quantified using background-subtracted images of presynaptic type Ib and II boutons. To obtain DCV size, the ILP2-GFP channel was subjected to Gaussian blur (σ = 20 nm) and thresholded before using Fiji’s particle analysis plugin to obtain the area of individual particles, from which, assuming a circular shape, the diameter was calculated. The analysis was restricted to particles with a circularity above 0.80. To obtain the size of small VMAT-positive vesicles, representative isolated vesicles were selected in the VMAT channel in areas devoid of ILP2-GFP signal. The vesicle diameter was measured as the full width at half maximum (FWHM) on a Gaussian fit of the intensity profile.

To determine the size of the vesicles constituting the HA-VMAT aggregates in cell bodies of *Rab2* larvae, 50 representative, isolated vesicles on STED images of 10 cell bodies in four larvae, located either in the rim of densely packed aggregates or in aggregates with moderate vesicle density, were selected for measuring the FWHM of Gaussian fitted intensity profiles.

### Candidate protein analysis

Documentation concerning the subcellular localization and functional annotation of biotinylated candidate proteins was obtained from open-source bioinformatics databases (https://flybase.org/, https://www.uniprot.org/), updated through searches of the recent literature. The information summarized in Table S2 is based on studies of both the *Drosophila* proteins and their closest human orthologs.

Cutoff criteria for significantly biotinylated candidate proteins in volcano plots were an adjusted P value <0.05 and a fold change exceeding 2. When calculating the rank correlation between Rab2-related and VMAT-related candidate proteins, the highest rank was used in cases where the same protein was detected more than once in the same set.

### AlphaFold modelling

Protein structure modelling of the *Drosophila* Rab2:Syd (2:2) complex was performed with ColabFold (Mirdita et al., 2022) version 1.5.5 (AlphaFold2 [Jumper et al., 2021]) using MMSeq2) using the AlphaFold2_Multimer setting and relaxation. The crystal structure of active *Drosophila* Rab2 bound to GppNHp (PDB: 4rke, (Lardong et al., 2015)) was used as a template.

Modelling of the RUFY dimer was performed with AlphaFold 3 (Abramson et al., 2024) (https://alphafoldserver.com/).

### MD simulations

The AlphaFold structure was used for the Syd dimers. A Rab2 structure with bound Mg and GNP (PDB: 4rke) (Lardong et al., 2015) (without N-terminal residues GAMG and bound water) was aligned with the Rab2 from the AlphaFold structure, which was then removed, except for the C terminus, which was not resolved in the crystal structure. GNP was replaced with GTP. The construct was truncated to reduce computational time. The Syd dimers were truncated to residues L450–G526, and only one truncated Rab2 (M1-G188) was maintained in the complex. Mutant R468A, double mutant L465/R468A, and truncated construct ΔHelix-2 (Syd truncated after V504) were generated in PyMOL (The PyMOL Molecular Graphics System, Version 3.0 Schrödinger, LLC). The complex was solvated in a 20 × 9 × 9 nm box with TIP3P water and 100 mM NaCl. The simulations were run with GROMACS 2021.4 (Páll et al., 2020) and the CHARMM36m force field (Huang et al., 2017). The system was minimized, then equilibrated in 10 ps with a constant number of particles, volume, and temperature and 100 ps with a constant number of particles, pressure, and temperature (NPT). Both equilibrations were run with a 2 fs step size, v-rescale temperature coupling (time constant 0.1 ps) to keep temperature at 300 K, and the NPT equilibration was run with Berendsen isotropic pressure coupling (time constant 2 ps) to keep pressure at 1 bar. The proteins were restrained with position restraints during equilibration. The Particle Mesh Ewald algorithm (Wennberg et al., 2015) was used for long range, and LINCS algorithm was used to constrain hydrogens (Hess et al., 1997). The restraints were relieved, and a simulation was run for 2 ns with isotropic Parinello-Rahmen pressure coupling. The Rab2 (including Mg and GTP) was then pulled away from the Syd dimer using GROMACS built-in umbrella biasing potential with a rate of 0.01 nm/ps. Frames were taken from this pull simulation with Syd-Rab2 center-of-mass distances up to 7.5 nm in steps of 0.15 nm. Each of these frames was used for 10 ns simulations, with COM distance fixed using an umbrella biasing force of 1,000 kJ/mol/nm^2^. Potential of mean force (free energy of binding) was calculated from the umbrella simulations using the weighted histogram average method (Hub et al., 2010). The whole process (including solvation and minimization) was repeated 10 times for each construct, and the mean values and SEM were calculated.

### Sequence handling and alignment

Protein sequence alignment was performed in BioEdit (Hall T.A. 1999, BioEdit: a user-friendly biological sequence alignment editor and analysis program for Windows 95/98/NT) using the ClustalW multiple alignment function.

### Helical propensity estimation

Alpha helical propensity of the different regions of the Syd RH2 domain was evaluated using the NetSurfP -3.0 online tool from the Technical University of Denmark (Hoie et al., 2022). https://services.healthtech.dtu.dk/services/NetSurfP-3.0/

### Statistical analysis

Data visualization in graphs was performed with Excel software (Microsoft), which was also used for *t* tests. ANOVA and Dunnett’s test were executed with JMP sofware (JMP Statistical Discovery). Before analysis, datasets were assessed for homogeneity of variances with a test battery, including Bartlett’s test, and the residuals were checked for normal distribution with Shapiro–Wilk’s test. Data failing to conform to normality or homoscedasticity were logarithmically transformed, or the nonparametric Steel with control test was applied, as appropriate. P values <0.05 were considered significant, indicated with red text color in the figures. All performed Dunnett’s tests and *t* tests were two-sided. The experimental unit was larva (i.e., one larva was represented with one value, usually the average of repeated measurements). In tests with a nonsignificant outcome when comparing group means, the sample size was at least four third instar larvae per group. Details of individual statistical tests, including sample size, are provided in the Source File. The rank correlation between Rab2-related and VMAT-related candidate proteins was calculated using Excel, including only proteins present in both datasets with fold change >2 and *P*_adj_ < 0.05, and using the highest rank for proteins represented more than once in the same set.

### Online supplemental material

Fig. S1 elaborates on Rab2- and VMAT-specific PB-MS results and shows colocalization of ILP2-GFP and TurboID-VMAT in the motor neuron soma and synaptic bouton. Fig. S2 shows additional details of the Rab2–Syd Co-IP interaction, the predicted structure of RUFY, and the RUFY-DLIC Co-IP. Fig. S3 shows DCV axonal transport speeds calculated from the data in Fig. 3, the defect in lysosomal transport of spin-GFP–positive lysosomes in *Rab2* null larvae, and the apparently normal axonal DCV transport in *prd1* null larvae. Fig. S4 shows the effect of disruption of different Rab and Arf GTPases on DCV axonal transport. Fig. S5 shows the effect on axonal DCV transport of the RNAi knockdown of different proteins involved in trafficking of DCV membrane proteins or suspected to play a role in motor adaptor recruitment. Fig. S6 shows axonal transport and neuronal distribution of DCV in LRRK null larvae, decreased peripheral nerve DCV content during RUFY RNAi knockdown, and decreased DCV content in *syd* mutant NMJs. Fig. S7 shows trafficking defects of HA-VMAT and SYTα-mCherry in motor neuron somata and axons of *Rab2* null larvae, evaluated by STED microscopy. Video 1 displays DCV axonal transport in peripheral nerves of larvae of different genotypes; the same data are shown as kymographs and analyzed in Figs. 3 and S3. Table S1 lists fly strains used in this study and their origin. Table S2 lists recombinant DNAs used in this study. Table S3 lists antibodies, chemicals, and reagents used in this study. Table S4 lists the genotypes of flies used in all experiments in this study. Data S1 provides the full results of the active Rab2-specific PB-MS. Data S2 provides the results of active Rab2-specific PB-MS with annotation of hits with FC > 4. Data S3 provides the full results of VMAT-specific PB-MS. Data S4 provides the source data for all figures.

## Supplementary Material

Table S1 shows *Drosophila* lines.

Table S2 shows recombinant DNAs.

Table S3 shows antibodies and reagents.

Table S4 shows *Drosophila* genotypes used in figures and Video 1.

Data S1 is a table containing fold change and Student’s *t* test statistics of biotinylated protein label-free quantification (LFQ) intensities from flies with pan-neuronal expression of TurboID-Rab2^Q65L^ (*elav > 2xHA-TurboID-Rab2*^*Q65L*^) relative to TurboID-Rab2^S20N^ (*elav > 2xHA-TurboID-Rab2*^*S20N*^).

Data S2 is a table containing annotation of the candidate proteins (hits) from Supplementary Table 1 that exhibit a fold change in biotinylation intensity of at least four for TurboID-Rab2Q65L relative to TurboID-Rab2^S20N^.

Data S3 is a table containing fold change and Student’s *t* test statistics of biotinylated protein LFQ intensities from flies with pan-neuronal expression of TurboID-VMAT (*elav > ILP2-GFP, TurboID-HA-VMAT*) relative to free cytosolic TurboID (*elav > ILP2-GFP, TurboID*).

Data S4 provides the source data for all figures.

SourceData F2 is the source file for Fig. 2.

SourceData F5 is the source file for Fig. 5.

SourceData FS2 is the source file for Fig. S2.

## Data Availability

The MS proteomics data have been deposited to the ProteomeXchange Consortium (http://proteomecentral.proteomexchange.org) through the PRIDE partner repository. The data set identifiers are PXD063196 (https://www.ebi.ac.uk/pride/archive/projects/PXD063196) and PXD063200 (https://www.ebi.ac.uk/pride/archive/projects/PXD063200).
