# Supplementary material for: A Syd and RUFY dynein adaptor complex mediates axonal circulation of dense core vesicles
Source: J Cell Biol. 2026 Jan 6;225(3):e202507071. doi: 10.1083/jcb.202507071 (PMC12772503; doi:10.1083/jcb.202507071)
Supplement: Table S1 — shows Drosophila lines. [file jcb_202507071_tables1.docx]

**Supplementary Table 1.**

Drosophila lines.

| **Line** | **Source** | **Identifier** |
| --- | --- | --- |
| *elav-Gal4 (X)* | BDSC | BDSC #458 |
| *OK6-Gal4 (II)* | BDSC | BDSC #64199 |
| *UAS-ILP2-Gal4 (II)* | Dr. Edwin S Levitan (Wong et al., 2012) | N/A |
| *UAS-spinster-Venus* | Dr. Alyssa Johnson | N/A |
| *UAS-DCR2 (X)* | BDSC | BDSC #24645 |
| *UAS-TurboID-2xHA-Rab2[Q65L] (ZH-86Fb)* | (Lund et al., 2018) | N/A |
| *UAS-TurboID-2xHA-Rab2[S20N] (ZH-86Fb)* | (Lund et al., 2018) | N/A |
| *UAS-TurboID-HA-VMAT (ZH-86Fb)* | this paper | N/A |
| *UAS-TurboID (ZH-86Fb)* | this paper | N/A |
| *UAS-HA-VMAT (ZH-86Fb)* | this paper | N/A |
| *UAS-HA-VMAT[D584A, E585A, L589A, I590A, Y600A] (ZH-86Fb)* | this paper | N/A |
| *UAS-HA-VMAT (II)* | Dr. David Krantz (Grygoruk et al., 2014) | N/A |
| *UAS-HA-VMAT (III)* | Dr. David Krantz (Grygoruk et al., 2014) | N/A |
| *UAS-HA-VMAT[Y600A] (III)* | Dr. David Krantz (Grygoruk et al., 2014) | N/A |
| *UAS-syt-α-mCherry (III)* | Dr. Paul Taghert (Park et al., 2014) | N/A |
| *Rab2[Δ1]* | (Lund et al., 2018) | N/A |
| *syd[z4] Diap1[th-1] st[1] cu[1] sr[1] e[s] ca[1]* | BDSC | BDSC #32016 |
| *unc-104[P350]* | Dr. William Saxton (Barkus et al., 2008) | N/A |
| *unc-104[O3.1]* | Dr. William Saxton (Barkus et al., 2008) | N/A |
| *PBac{w[+mC]=RB}Lrrk[e03680]* | BDSC | BDSC #85160 |
| *Rab3[rup]* | Dr. Robin Hiesinger (Graf et al., 2009) | N/A |
| *Arf6[GX16w-]* | BDSC | BDSC #60585 |
| *Rab8[1]* | Dr. Robin Hiesinger (Giagtzoglou et al., 2012) | N/A |
| *Rab26[exon1-2delta]* | Dr. Robin Hiesinger (Kohrs et al., 2021) | N/A |
| *Rab32[AR]* | Dr. Robin Hiesinger (Reaume et al., 1991) | N/A |
| *Rab10[KO]* | Dr. Robin Hiesinger (Kohrs et al., 2021) | N/A |
| *Prd1[M56]* | BDSC | BDSC #37744 |
| *RabX5[e04143]* | BDSC | BDSC #18228 |
| *Df(3L)BSC795* | BDSC | BDSC #27367 |
| *Df(3R)BSC141* | BDSC | BDSC #9501 |
| *Df(2R)BSC639* | BDSC | BDSC #25729 |
| *Df(2R)BSC346* | BDSC | BDSC #24370 |
| *Df(3L)BSC445* | BDSC | BDSC #24949 |
| *Df(2R)BSC279* | BDSC | BDSC #23664 |
| *Df(3R)Exel7310* | BDSC | BDSC #7965 |
| *UAS-Dhc64C-RNAi[TRiP.HMS01587]* | BDSC | BDSC #36698 |
| *UAS-Khc-RNAi[TRiP.GL00330]* | BDSC | BDSC #35409 |
| *UAS-Rab11-RNAi[KK108297]* | VDRC | VDRC id 108382 |
| *UAS-Rab11-RNAi[GD11761]* | VDRC | VDRC id 22198 |
| *UAS-Rab1-RNAi[VSH330620]* | VDRC | VDRC id 330620 |
| *UAS-RUFY/CG31064-RNAi[TRiP.HMC03246] VAL20* | BDSC | BDSC #51494 |
| *UAS-RUFY/CG31064-RNAi[KK100333]* | VDRC | VDRC id 103379 |
| *UAS-CG6707-RNAi[TRiP.JF02947] VAL10* | BDSC | BDSC #28316 |
| *UAS-CG6707-RNAi[KK108707]* | VDRC | VDRC id 110291 |
| *UAS-Vps35-RNAi[TRiP.HMS01858] VAL20* | BDSC | BDSC #38944 |
| *UAS-ruby-RNAi[TRiP.HMS00479] VAL20* | BDSC | BDSC #32477 |
| *UAS-Rab4-RNAi[TRiP.HMS01100] VAL20* | BDSC | BDSC #33757 |
| *UAS-Rab14-RNAi[TRiP.JF03135] VAL10* | BDSC | BDSC #28708 |
| *UAS-dNischarin/CG11807-RNAi[GD7390]* | VDRC | VDRC id 38566 |
| *UAS-Hep.Act (II)* | BDSC | BDSC #9306 |
| *UAS-bsk.DN (X)* | BDSC | BDSC #6409 |
