# Supplementary material for: A Syd and RUFY dynein adaptor complex mediates axonal circulation of dense core vesicles
Source: J Cell Biol. 2026 Jan 6;225(3):e202507071. doi: 10.1083/jcb.202507071 (PMC12772503; doi:10.1083/jcb.202507071)
Supplement: Table S2 — shows recombinant DNAs. [file jcb_202507071_tables2.docx]

**Supplementary Table 2.**

Recombinant DNAs.

| **Recombinant DNA** | **Source** | **Identifier** |
| --- | --- | --- |
| pcDNA3.1(+)_3xHA-Rab2 | (Lund et al., 2021) | N/A |
| pcDNA3.1(+)_3xHA-Rab2[S20N] | (Lund et al., 2021) | N/A |
| pcDNA3.1(+)_3xHA-Rab2[Q65L] | (Lund et al., 2021) | N/A |
| pcDNA3.1(+)_3xmyc-Rab2[Q65L] | This paper (Genscript) | N/A |
| pcDNA3.1(+)_2xmyc-2xpc-Syd | This paper (Genscript) | N/A |
| pcDNA3.1(+)_2xmyc-2xpc-Syd-N1 | This paper (Genscript) | N/A |
| pcDNA3.1(+)_2xmyc-2xpc-Syd-N2 | This paper (Genscript) | N/A |
| pcDNA3.1(+)_2xmyc-2xpc-Syd-N3 | This paper (Genscript) | N/A |
| pcDNA3.1(+)_2xmyc-2xpc-Syd-N4 | This paper (Genscript) | N/A |
| pcDNA3.1(+)_2xmyc-2xpc-Syd-N5 | This paper (Genscript) | N/A |
| pcDNA3.1(+)_2xmyc-2xpc-Syd-C1 | This paper (Genscript) | N/A |
| pcDNA3.1(+)_2xmyc-2xpc-Syd-N2[L465A,R468A] | This paper (Genscript) | N/A |
| pcDNA3.1(+)_2xmyc-2xpc-Syd-N2[4A] | This paper (Genscript) | N/A |
| pcDNA3.1(+)_2xmyc-2xpc-Syd-N2[9A] | This paper (Genscript) | N/A |
| pcDNA3.1(+)_2xmyc-2xpc-Syd-N2[7A] | This paper (Genscript) | N/A |
| pcDNA3.1(+)_3xHA-Syd-N2 | This paper (Genscript) | N/A |
| pcDNA3.1(+)_3xHA-RUFY | This paper (Genscript) | N/A |
| pcDNA3.1(+)_3xmyc-RUFY | This paper (Genscript) | N/A |
| pcDNA3.1(+)_3xV5-RUFY | This paper (Genscript) | N/A |
| pcDNA3.1(+)_3xmyc-dNischarin | This paper (Genscript) | N/A |
| pcDNA3.1(+)_Arl8-3xV5 | This paper (Genscript) | N/A |
| pcDNA3.1(+)_3xFLAG-Klc | This paper (Genscript) | N/A |
| pcDNA3.1(+)_3xV5-DLIC | This paper (Genscript) | N/A |
| pCMV5-FLAG-LRRK2[G2019S] | MRC PPU Reagents and Services | DU10129 |
| pUASTattB-TurboID-HA-VMAT | This paper (Genscript) | N/A |
| pUASTattB-TurboID | This paper (Genscript) | N/A |
| pUASTattB-HA-VMAT | This paper (Genscript) | N/A |
| pUASTattB-HA-VMAT[D584A, E585A, L589A, I590A, Y600A] | This paper (Genscript) | N/A |
