# Supplementary material for: A Syd and RUFY dynein adaptor complex mediates axonal circulation of dense core vesicles
Source: J Cell Biol. 2026 Jan 6;225(3):e202507071. doi: 10.1083/jcb.202507071 (PMC12772503; doi:10.1083/jcb.202507071)
Supplement: Table S3 — shows antibodies and reagents. [file jcb_202507071_tables3.docx]

**Supplementary Table 3.**

Antibodies and reagents.

| **Antibodies** | **Source** | **Identifier** |
| --- | --- | --- |
| Rat anti-HA 3F10 (WB and IHC, 100 ng/ml) | Sigma-Aldrich | Ref: 11867423001, Lot: 47877600 |
| Mouse anti-HA 6E2 HRP (WB, 1:1000-5000) | Cell Signaling Technology | Ref: 2999S, Lot: 4 |
| Rabbit anti-myc (WB, 1:1000) | Sigma-Aldrich, EMD Millipore Corp. | Ref: 06-549, Lot: 3445807 |
| Rabbit anti-V5 (WB, 1:500) | GenScript | Cat: A00623-40, Lot: 19K002008 |
| Mouse anti-FLAG (WB, 1:1000) | Invitrogen | Ref: MA1-91878, Lot: WF324531 |
| Rabbit anti-mCherry (IHC, 1:100) | Invitrogen | Ref: PA5-34974, Lot: WI3386681C |
| Alpaca anti-GFP VHH single domain antibody/nanobody, custom conjugated to abberior STAR ORNANGE (IHC: 1:100 from 0.5 mg/ml stock) | Chromotech, this paper | Ref: gt-250, Lot: 71017001U |
| Goat anti-rat HRP (WB, 1:8300) | Cell Signaling Technology | Ref: #7077S 01/2012, Lot: 8 |
| Goat anti-rabbit HRP (WB, 1:8300) | Cell Signaling Technology | Ref: #7074S 12/2019, Lot: 28 |
| Goat anti-mouse HRP (WB, 1:2000) | ThermoFisher Scientific | Ref: 31430 |
| Goat anti-rat Abberior STAR RED (IHC, 1:500) | Abberior | Ref: STRED-1007-500UG, Lot: 00928PK-2 |
| Goat anti-rabbit Abberior STAR RED (IHC, 1:500) | Abberior | Ref: STRED-1002-500UG |
| **Chemicals, peptides, and recombinant proteins** | **Source** | **Identifier** |
| Flystuff Nutri-Fly Bloomington Formulation medium | Scientific Laboratory Supplies | FLY1034 |
| GppNHp/GMP-PNP | Sigma-Aldrich | Ref: 0635-25MG, Lot: SLCC2098, SLCN9946 |
| Sylgard 184 Elastomer Kit | Farnell | Ref: 101697 |
| Mli-2 | Abcam | Ref: ab147471, Lot: GR3406958 |
| 1,6 Hexanediol | Sigma-Aldrich | Ref: 240117-50G |
| abberior STAR ORANGE, NHS ester | abberior | Ref: STORANGE-0002-1MG, Lot: 10319RK-1 |
| Pierce™ Anti-HA Magnetic Beads | Thermo Fisher Scientific | Ref: 88836, Lot: VJ307840, WF325730, UL291451, XD335925 |
| Dynabeads MyOne StreptavidinT1 | Thermo Fisher Scientific | Ref: 65601, Lot: 00804134 |
| SuperSignal ELISA Femto Maximum Sensitivity Substrate | Thermo Fisher Scientific | Ref: 37075 |
| Pierce™ Anti-c-Myc Magnetic Beads | Thermo Fisher Scientific | Ref: 88842, Lot: YL384976 |
| **Cell lines** | **Source** | **Identifier** |
| Human: HEK293 cells | ATCC | CRL-1573 |
