# Supplementary material for: A Syd and RUFY dynein adaptor complex mediates axonal circulation of dense core vesicles
Source: J Cell Biol. 2026 Jan 6;225(3):e202507071. doi: 10.1083/jcb.202507071 (PMC12772503; doi:10.1083/jcb.202507071)
Supplement: Table S4 — shows Drosophila genotypes used in figures and Video 1. [file jcb_202507071_tables4.docx]

**Supplementary Table 4.**

Drosophila genotypes used in Figures and Video S1.

| **Figure or Video** | **Panel** | **Genotype** |
| --- | --- | --- |
| Fig. 1 | B, C | *elav-Gal4/+(/Y);; UAS-2xHA-TurboID-Rab2^Q65L^/+* |
|  |  | *elav-Gal4/+(/Y);; UAS-2xHA-TurboID-Rab2^S20N^/+* |
|  | D | *elav-Gal4/+(/Y); UAS-Ilp2-GFP/+; UAS-TurboID-HA-VMAT/+* |
|  | E | *elav-Gal4/+(/Y); UAS-Ilp2-GFP/+; UAS-TurboID-HA-VMAT/+* |
|  |  | *elav-Gal4/+(/Y); UAS-Ilp2-GFP/+; UAS-TurboID/+* |
|  | F, G | *elav-Gal4/+(/Y);; UAS-2xHA-TurboID-Rab2^Q65L^/+* |
|  |  | *elav-Gal4/+(/Y);; UAS-2xHA-TurboID-Rab2^S20N^/+* |
|  |  | *elav-Gal4/+(/Y); UAS-Ilp2-GFP/+; UAS-TurboID-HA-VMAT/+* |
|  |  | *elav-Gal4/+(/Y); UAS-Ilp2-GFP/+; UAS-TurboID/+* |
| Fig. 3 | A-H | *OK6-Gal4 UAS-Ilp2-GFP/+* |
|  |  | *Rab2^Δ1^ OK6-Gal4/Rab2 ^Δ1^ UAS-Ilp2-GFP* |
|  |  | *OK6-Gal4 UAS-Ilp2-GFP/+; syd^z4^ Diap1^th-1^ st^1^ cu^1^ sr^1^ e^s^ ca^1^/Df(3L)BSC795* |
|  |  | *OK6-Gal4 UAS-Ilp2-GFP/+; UAS-Dhc64C-RNAi^TRiP.HMS01587^/+* |
|  |  | *OK6-Gal4 UAS-Ilp2-GFP/+; UAS-Khc-RNAi^TRiP.GL00330^/+* |
|  |  | *OK6-Gal4 UAS-Ilp2-GFP/+; Arl8^e00336^/Df(3R)BSC222* |
|  |  | *unc-104^P350^/unc104^O3.1^ OK6-Gal4 UAS-Ilp2-GFP* |
| Fig. 4 | A | *OK6-Gal4 UAS-Ilp2-GFP/+* |
|  |  | *Rab2^Δ1^ OK6-Gal4/ Rab2^Δ1^ UAS-Ilp2-GFP* |
|  |  | *OK6-Gal4 UAS-Ilp2-GFP/+; syd^z4^ Diap1^th-1^ st^1^ cu^1^ sr^1^ e^s^ ca^1^/Df(3L)BSC795* |
|  |  | *OK6-Gal4 UAS-Ilp2-GFP/+; UAS-Dhc64C-RNAi^TRiP.HMS01587^/+* |
|  |  | *OK6-Gal4 UAS-Ilp2-GFP/+; UAS-Khc-RNAi^TRiP.GL00330^/+* |
|  |  | *OK6-Gal4 UAS-Ilp2-GFP/+; Arl8^e00336^/Df(3R)BSC222* |
|  |  | *unc-104^P350^/unc104^O3.1^ OK6-Gal4 UAS-Ilp2-GFP* |
|  | B-D | *OK6-Gal4 UAS-Ilp2-GFP/+* |
|  |  | *Rab2^Δ1^ OK6-Gal4/Rab2^Δ1^ UAS-Ilp2-GFP* |
|  |  | *OK6-Gal4 UAS-Ilp2-GFP/+; syd^z4^ Diap1^th-1^ st^1^ cu^1^ sr^1^ e^s^ ca^1^/Df(3L)BSC795* |
|  | E, F | *OK6-Gal4/UAS-spinster-Venus* |
|  |  | *OK6-Gal4/UAS-spinster-Venus; syd^z4^ Diap1^th-1^ st^1^ cu^1^ sr^1^ e^s^ ca^1^/Df(3L)BSC795* |
| Fig. 5 | C | *UAS-DCR2/+(/Y); OK6-Gal4 UAS-Ilp2-GFP/+* |
|  |  | *UAS-DCR2/+(/Y); OK6-Gal4 UAS-Ilp2-GFP/UAS-CG31064-RNAi^KK100333^* |
|  | D | *UAS-DCR2/+(/Y); OK6-Gal4 UAS-Ilp2-GFP/+* |
|  |  | *UAS-DCR2/+(/Y); OK6-Gal4 UAS-Ilp2-GFP/UAS-CG31064-RNAi^KK100333^* |
|  |  | *OK6-Gal4 UAS-Ilp2-GFP/+* |
|  |  | *OK6-Gal4 UAS-Ilp2-GFP/+; UAS-CG31064-RNAi^TRiP.HMC03246^/+* |
|  | E, F | *D42-Gal4 UAS-ANF-GFP/+* |
|  |  | *Blos1^ex2^; D42-Gal4 UAS-ANF-GFP/+* |
|  | G-I | *OK6-Gal4 UAS-Ilp2-GFP/+* |
|  |  | *OK6-Gal4 UAS-Ilp2-GFP/+; syd^z4^ Diap1^th-1^ st^1^ cu^1^ sr^1^ e^s^ ca^1^/Df(3L)BSC795* |
|  |  | *Rab2^Δ1^ OK6-Gal4/Rab2^Δ1^ UAS-Ilp2-GFP; syd^z4^ Diap1^th-1^ st^1^ cu^1^ sr^1^ e^s^ ca^1^/Df(3L)BSC795* |
| Fig. 6 | A-C | *OK6-Gal4 UAS-Ilp2-GFP/+* |
|  |  | *UAS-Bsk^DN^/+(/Y); OK6-Gal4 UAS-Ilp2-GFP/+* |
|  | D-F | *OK6-Gal4 UAS-Ilp2-GFP/+* |
|  |  | *UAS-Bsk^DN^/+(/Y); OK6-Gal4 UAS-Ilp2-GFP/+* |
|  |  | *OK6-Gal4 UAS-Ilp2-GFP/UAS-Hep^Act^* |
| Fig. 7 | A | *OK6-Gal4 UAS-Ilp2-GFP/+; UAS-HA-VMAT/+* |
|  |  | *Rab2^Δ1^ OK6-Gal4/Rab2^Δ1^ UAS-Ilp2-GFP; UAS-HA-VMAT/+* |
|  |  | *OK6-Gal4 UAS-Ilp2-GFP/UAS-HA-VMAT; syd^z4^ Diap1^th-1^ st^1^ cu^1^ sr^1^ e^s^ ca^1^/Df(3L)BSC795* |
|  |  | *OK6-Gal4 UAS-Ilp2-GFP/+; UAS-Dhc64C-RNAi^TRiP.HMS01587^/UAS-HA-VMAT* |
|  |  | *OK6-Gal4 UAS-Ilp2-GFP/UAS-HA-VMAT; Arl8^e00336^/Df(3R)BSC222* |
|  | B, C | *OK6-Gal4 UAS-Ilp2-GFP/+; UAS-HA-VMAT/+* |
|  |  | *Rab2^Δ1^ OK6-Gal4/Rab2^Δ1^ UAS-Ilp2-GFP; UAS-HA-VMAT/+* |
|  |  | *OK6-Gal4 UAS-Ilp2-GFP/UAS-HA-VMAT; syd^z4^ Diap1^th-1^ st^1^ cu^1^ sr^1^ e^s^ ca^1^/Df(3L)BSC795* |
|  |  | *OK6-Gal4 UAS-Ilp2-GFP/+; UAS-Dhc64C-RNAi^TRiP.HMS01587^/UAS-HA-VMAT* |
|  |  | *OK6-Gal4 UAS-Ilp2-GFP/UAS-HA-VMAT; Arl8^e00336^/Df(3R)BSC222* |
|  |  | *OK6-Gal4 UAS-Ilp2-GFP/+; UAS-RUFY/UAS-CG31064-RNAi^TRiP.HMC03246^/UAS-HA-VMAT* |
|  | D, E | *Rab2^Δ1^ OK6-Gal4/Rab2^Δ1^ UAS-Ilp2-GFP; UAS-HA-VMAT/+* |
|  | F, G | *OK6-Gal4 UAS-Ilp2-GFP/+; UAS-HA-VMAT/+* |
|  |  | *OK6-Gal4 UAS-Ilp2-GFP/+; UAS-HA-VMAT[DE/LI/Y>A]/+* |
|  |  | *Rab2^Δ1^ OK6-Gal4/Rab2^Δ1^ UAS-Ilp2-GFP; UAS-HA-VMAT/+* |
|  |  | *Rab2^Δ1^ OK6-Gal4/Rab2^Δ1^ UAS-Ilp2-GFP; UAS-HA-VMAT[DE/LI/Y>A]/+* |
| Fig. S1 | A | *elav-Gal4/+(/Y);; UAS-2xHA-TurboID-Rab2^Q65L^/+* |
|  |  | *elav-Gal4/+(/Y);; UAS-2xHA-TurboID-Rab2^S20N^/+* |
|  | B | *elav-Gal4/+(/Y);; UAS-2xHA-TurboID-Rab2^Q65L^/+* |
|  |  | *elav-Gal4/+(/Y);; UAS-2xHA-TurboID-Rab2^S20N^/+* |
|  |  | *elav-Gal4/+(/Y); UAS-Ilp2-GFP/+; UAS-TurboID-HA-VMAT/+* |
|  |  | *elav-Gal4/+(/Y); UAS-Ilp2-GFP/+; UAS-TurboID/+* |
|  | C | *elav-Gal4/+(/Y); UAS-Ilp2-GFP/+; UAS-TurboID-HA-VMAT/+* |
| Fig. S3 | A, B | *OK6-Gal4 UAS-Ilp2-GFP/+* |
|  |  | *Rab2^Δ1^ OK6-Gal4/Rab2^Δ1^ UAS-Ilp2-GFP* |
|  |  | *OK6-Gal4 UAS-Ilp2-GFP/+; syd^z4^ Diap1^th-1^ st^1^ cu^1^ sr^1^ e^s^ ca^1^/Df(3L)BSC795* |
|  |  | *OK6-Gal4 UAS-Ilp2-GFP/+; UAS-Dhc64C-RNAi^TRiP.HMS01587^/+* |
|  |  | *OK6-Gal4 UAS-Ilp2-GFP/+; UAS-Khc-RNAi^TRiP.GL00330^/+* |
|  |  | *OK6-Gal4 UAS-Ilp2-GFP/+; Arl8^e00336^/Df(3R)BSC222* |
|  |  | *unc-104^P350^/unc104^O3.1^ OK6-Gal4 UAS-Ilp2-GFP* |
|  | C, D | *OK6-Gal4/+; UAS-spin.myc-GFP/+* |
|  |  | *Rab2^Δ1^ OK6-Gal4/Rab2^Δ1^ ; UAS-spin.myc-GFP/+* |
|  | E, F | *OK6-Gal4 UAS-Ilp2-GFP/+; prd1^m56^/Df(3R)Exel7310* |
|  |  | *OK6-Gal4 UAS-Ilp2-GFP/+* |
| Fig. S4 | A | *elav-Gal4/+(/Y); UAS-Ilp2-GFP/+; UAS-TurboID-HA-VMAT/+* |
|  |  | *elav-Gal4/+(/Y); UAS-Ilp2-GFP/+; UAS-TurboID/+* |
|  | B, C | *OK6-Gal4 UAS-Ilp2-GFP/+* |
|  |  | *Df(2R)BSC279 OK6-Gal4 UAS-Ilp2-GFP/Rab32^AR^* |
|  |  | *OK6-Gal4 UAS-Ilp2-GFP/+; Rab26^exon1-2D^* |
|  |  | *Df(2R)BSC639 OK6-Gal4 UAS-Ilp2-GFP/Rab3^rup^* |
|  |  | *OK6-Gal4 UAS-Ilp2-GFP/+; Rab8^1^/Df(3L)BSC445* |
|  |  | *Rab10^KO^(/Y); OK6-Gal4 UAS-Ilp2-GFP/+* |
|  |  | *OK6-Gal4 UAS-Ilp2-GFP/+; RabX5^e04143^* |
|  |  | *Arf6^GX16w-^/Df(2R)BSC346/OK6-Gal4 UAS-Ilp2-GFP* |
|  | D, E | *UAS-DCR2/+(/Y); OK6-Gal4 UAS-Ilp2-GFP/UAS-Rab11-RNAi^KK108297^* |
|  |  | *UAS-DCR2/+(/Y); OK6-Gal4 UAS-Ilp2-GFP/+; UAS-Rab11-RNAi^GD11761^/+* |
|  |  | *UAS-DCR2/+(/Y); OK6-Gal4 UAS-Ilp2-GFP/+* |
|  | F, G | *OK6-Gal4 UAS-Ilp2-GFP/+* |
|  |  | *OK6-Gal4 UAS-Ilp2-GFP/UAS-Rab1-RNAiVSH330620* |
| Fig. S5 | A | *UAS-DCR2/+(/Y); OK6-Gal4 UAS-Ilp2-GFP/+* |
|  |  | *UAS-DCR2/+(/Y); OK6-Gal4 UAS-Ilp2-GFP/+; UAS-Rab14-RNAi^TRiP.JF03135^/+* |
|  |  | *UAS-DCR2/+(/Y); OK6-Gal4 UAS-Ilp2-GFP/+; UAS-CG11807-RNAi^GD7390^/+* |
|  |  | *UAS-DCR2/+(/Y); OK6-Gal4 UAS-Ilp2-GFP/UAS-CG6707-RNAi^KK108707^* |
|  | B | *UAS-DCR2/+(/Y); OK6-Gal4 UAS-Ilp2-GFP/+* |
|  |  | *UAS-DCR2/+(/Y); OK6-Gal4 UAS-Ilp2-GFP/+; UAS-Rab14-RNAi^TRiP.JF03135^/+* |
|  |  | *UAS-DCR2/+(/Y); OK6-Gal4 UAS-Ilp2-GFP/+; UAS-CG11807-RNAi^GD7390^/+* |
|  |  | *UAS-DCR2/+(/Y); OK6-Gal4 UAS-Ilp2-GFP/UAS-CG6707-RNAi^KK108707^* |
|  |  | *UAS-DCR2/+(/Y); OK6-Gal4 UAS-Ilp2-GFP/+; UAS-CG6707-RNAi^TRiP.JF02947^/+* |
|  | C, D | *OK6-Gal4 UAS-Ilp2-GFP/+* |
|  |  | *OK6-Gal4 UAS-Ilp2-GFP/+; UAS-Rab4-RNAi^TRiP.HMS01100^/+* |
|  |  | *OK6-Gal4 UAS-Ilp2-GFP/+; UAS-ruby-RNAi^TRiP.HMS00479^/+* |
|  |  | *OK6-Gal4 UAS-Ilp2-GFP/UAS-Vps35-RNAi^TRiP.HMS01858^* |
| Fig. S6 | A-E | *OK6-Gal4 UAS-Ilp2-GFP/+* |
|  |  | *OK6-Gal4 UAS-Ilp2-GFP/+; Lrrk^e03680^/Df(3R)BSC141* |
|  | F | *UAS-DCR2/+(/Y); OK6-Gal4 UAS-Ilp2-GFP/+* |
|  |  | *UAS-DCR2/+(/Y); OK6-Gal4 UAS-Ilp2-GFP/UAS-CG31064-RNAi^KK100333^* |
|  |  | *OK6-Gal4 UAS-Ilp2-GFP/+* |
|  |  | *OK6-Gal4 UAS-Ilp2-GFP/+; UAS-CG31064-RNAi^TRiP.HMC03246^/+* |
|  | G, H | *OK6-Gal4 UAS-Ilp2-GFP/+* |
|  |  | *OK6-Gal4 UAS-Ilp2-GFP/+; syd^z4^ Diap1^th-1^ st^1^ cu^1^ sr^1^ e^s^ ca^1^/Df(3L)BSC795* |
| Fig. S7 | A, B | *OK6-Gal4 UAS-Ilp2-GFP/+; UAS-HA-VMAT^Y600A^/+* |
|  |  | *Rab2^Δ1^ OK6-Gal4/Rab2^Δ1^ UAS-Ilp2-GFP; UAS-HA-VMAT^Y600A^/+* |
|  | C, D | *OK6-Gal4 UAS-Ilp2-GFP/+; UAS-mCh-Syta/+* |
|  |  | *Rab2^Δ1^ OK6-Gal4/Rab2^Δ1^ UAS-Ilp2-GFP; UAS-mCh-Syta/+* |
|  | E | *Rab2^Δ1^ OK6-Gal4/Rab2^Δ1^ UAS-Ilp2-GFP; UAS-HA-VMAT/+* |
|  | F | *OK6-Gal4 UAS-Ilp2-GFP/+; UAS-HA-VMAT/UAS-CG31064-RNAi^TRiP.HMC03246^* |
|  | G, H | *OK6-Gal4 UAS-Ilp2-GFP/+; UAS-HA-VMAT/+* |
|  |  | *Rab2^Δ1^ OK6-Gal4/Rab2^Δ1^ UAS-Ilp2-GFP; UAS-HA-VMAT/+* |
| Video S1 | N/A | *OK6-Gal4 UAS-Ilp2-GFP/+* |
|  |  | *Rab2^Δ1^ OK6-Gal4/Rab2^Δ1^ UAS-Ilp2-GFP* |
|  |  | *OK6-Gal4 UAS-Ilp2-GFP/+; syd^z4^ Diap1^th-1^ st^1^ cu^1^ sr^1^ e^s^ ca^1^/Df(3L)BSC795* |
|  |  | *OK6-Gal4 UAS-Ilp2-GFP/+; UAS-Dhc64C-RNAi^TRiP.HMS01587^/+* |
|  |  | *OK6-Gal4 UAS-Ilp2-GFP/+; UAS-Khc-RNAi^TRiP.GL00330^/+* |
|  |  | *unc-104^P350^/unc104^O3.1^ OK6-Gal4 UAS-Ilp2-GFP* |
