# Supplementary figures and images for: A Syd and RUFY dynein adaptor complex mediates axonal circulation of dense core vesicles
Source: J Cell Biol. 2026 Jan 6;225(3):e202507071. doi: 10.1083/jcb.202507071 (PMC12772503; doi:10.1083/jcb.202507071)

2A

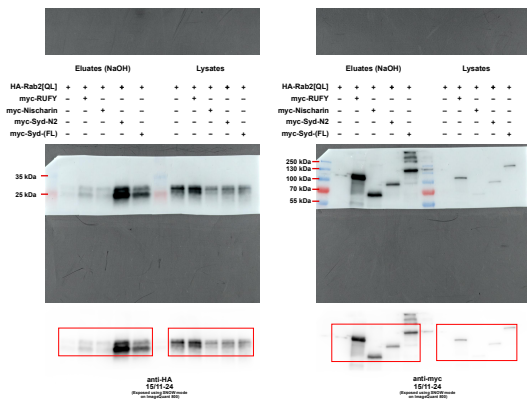

2B

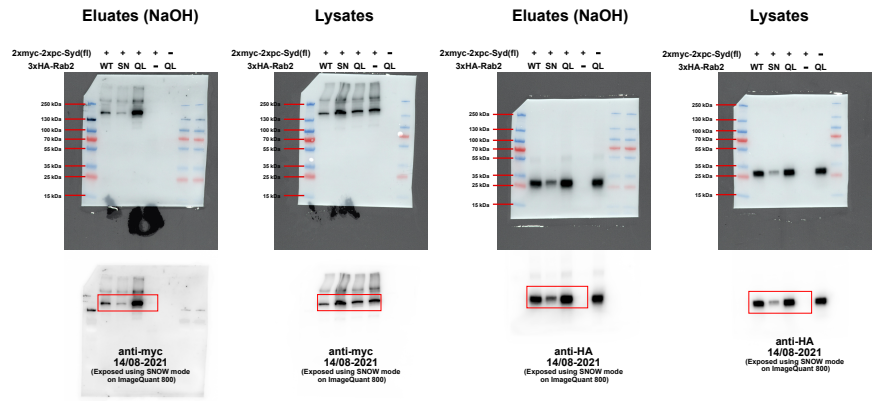

2D

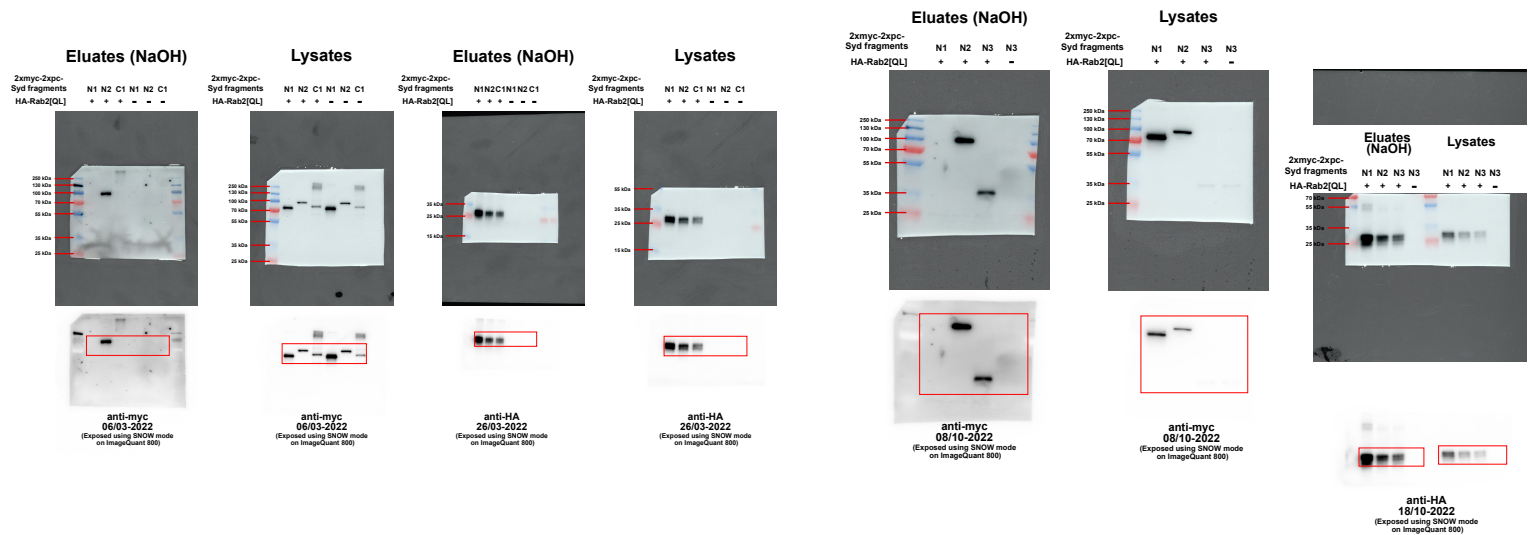

2F

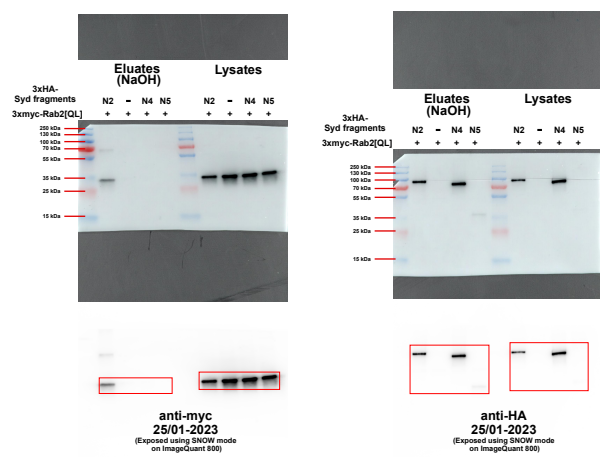

2G

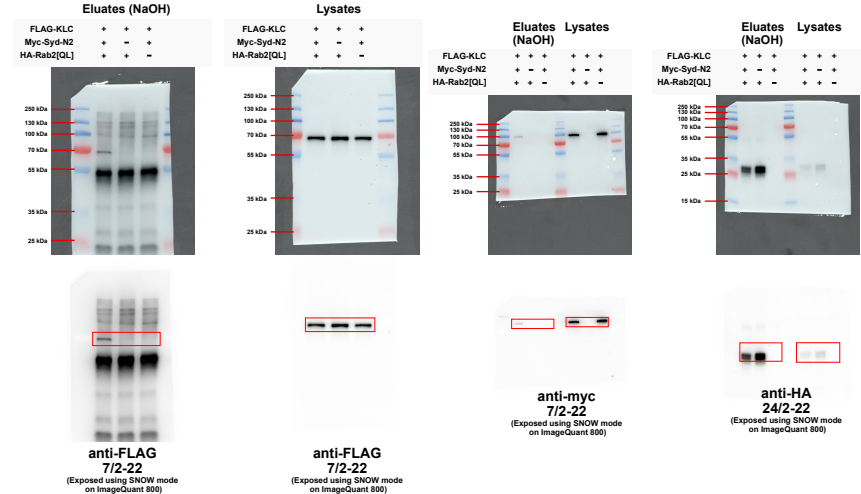

2I

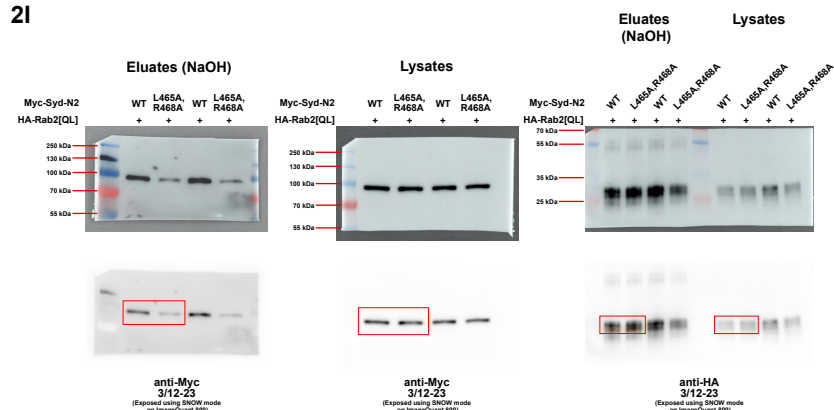

2K

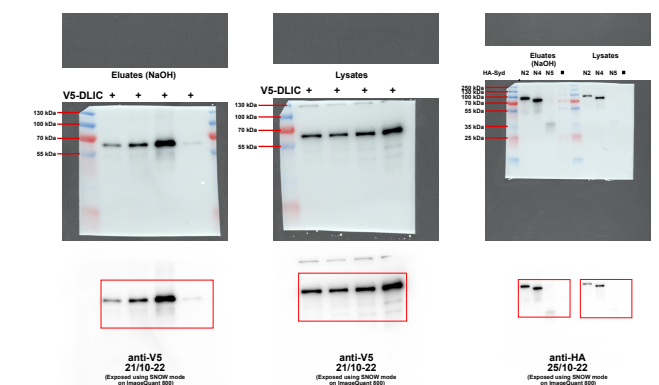

Supplement: SourceData F2 — is the source file for Fig. 2. [file jcb_202507071_sourcedataf2.pdf]

5A

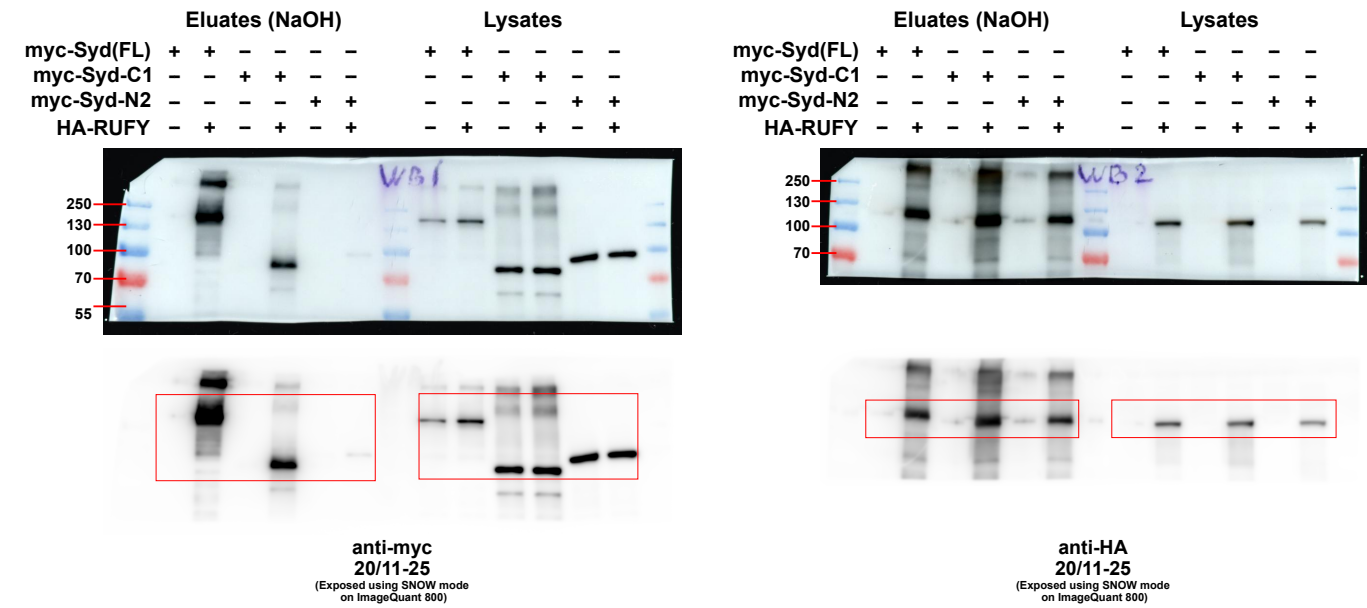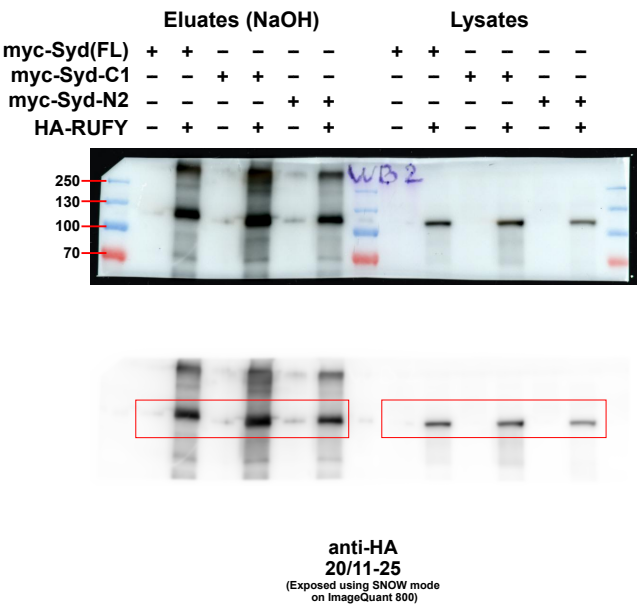

5B

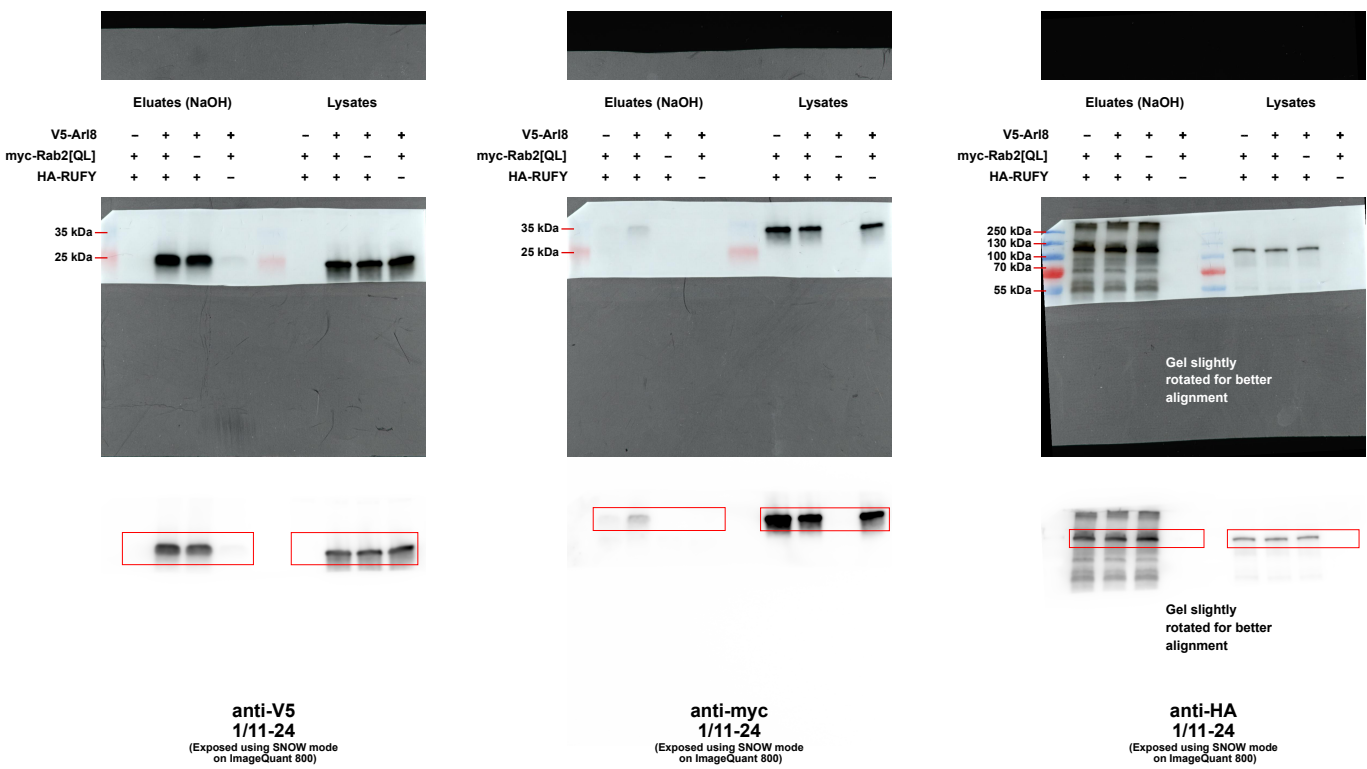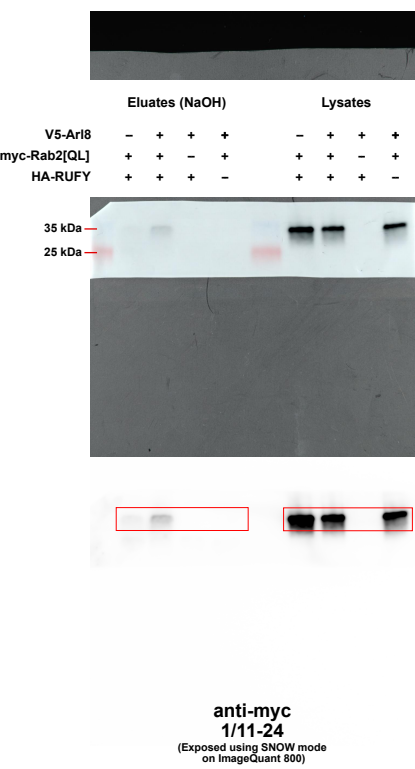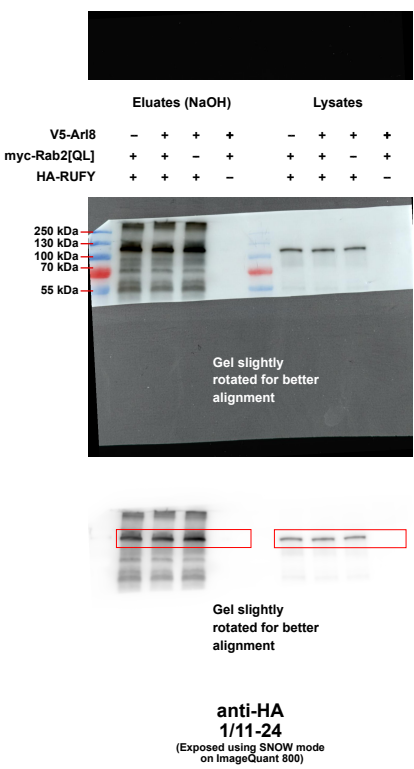

Supplement: SourceData F5 — is the source file for Fig. 5. [file jcb_202507071_sourcedataf5.pdf]

S2A

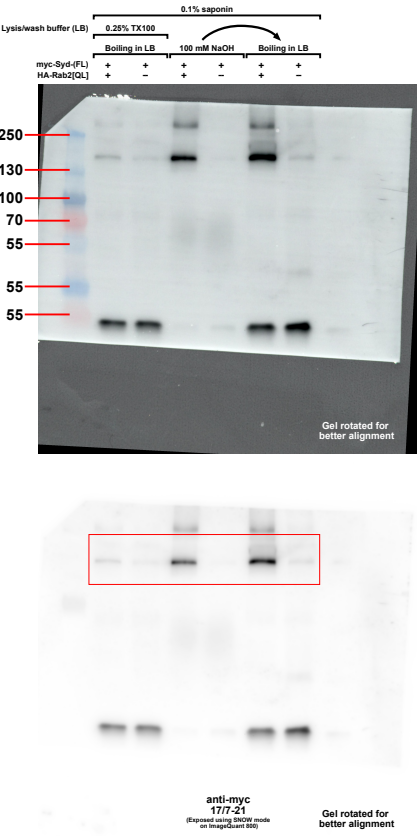

S2D

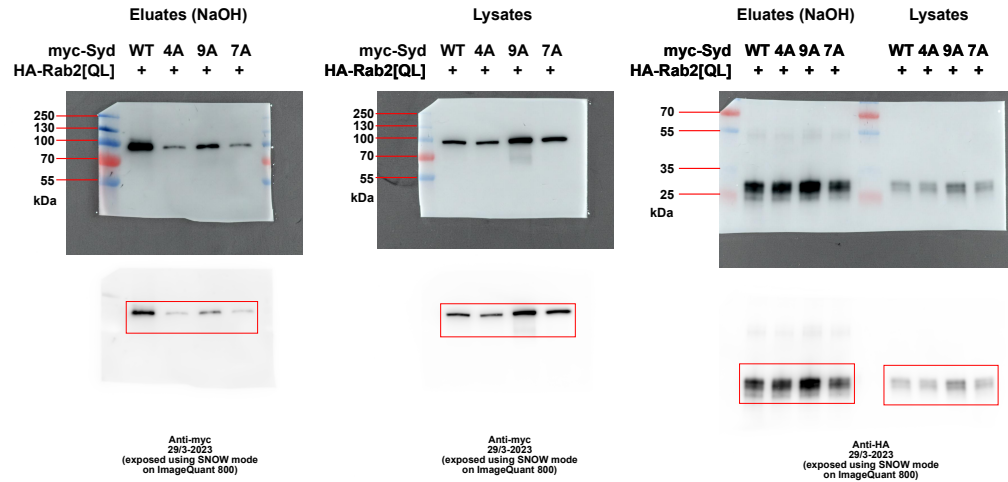

S2E

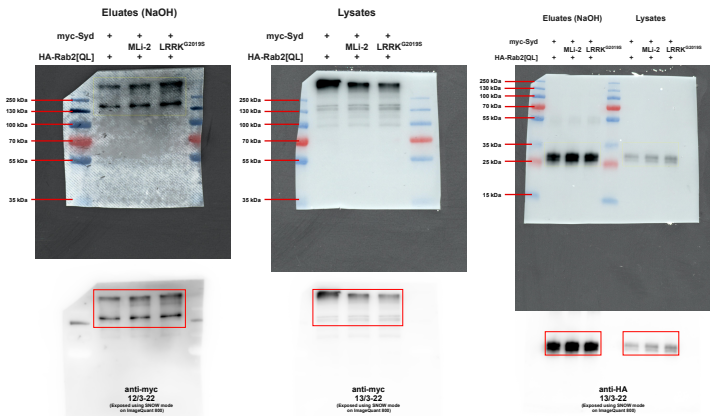

S2G

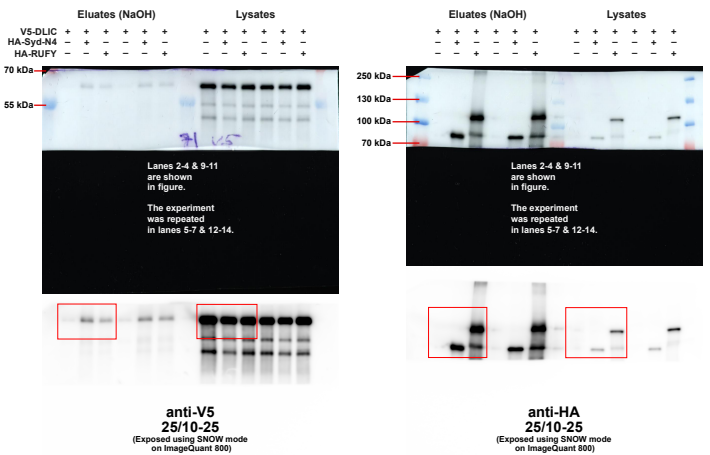

S2B

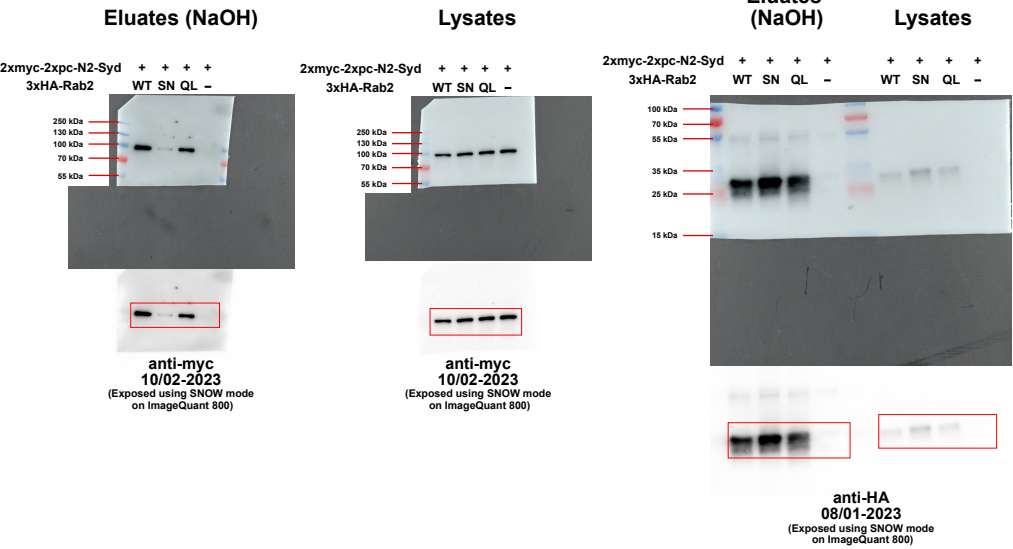

Supplement: SourceData FS2 — is the source file for Fig. S2. [file jcb_202507071_sourcedatafs2.pdf]
